# Beyond person‐centered approaches: Integrating individual, sociodemographic and socio‐spatial factors to better understand active and sustainable mobility

**DOI:** 10.1111/aphw.70136

**Published:** 2026-03-06

**Authors:** Claudia Teran‐Escobar, Kamila Tabaka, Sonia Chardonnel, Sarah Duché, Aïna Chalabaev

**Affiliations:** ^1^ Univ. Grenoble Alpes, SENS F‐38000 Grenoble France; ^2^ School of Political Studies Univ. Grenoble Alpes, CNRS, Sciences Po Grenoble ‐ UGA*, Pacte Grenoble 38000 France; ^3^ Department of Psychology University Paris Nanterre Nanterre France

**Keywords:** active and sustainable mobility, biking, car shift, multiple‐level factors, walking

## Abstract

Although several determinants of travel mode choices have been identified, most research has adopted a mono‐disciplinary perspective. This study simultaneously investigated individual, socio‐spatial (at the national and local level), and sociodemographic factors of active and sustainable mobility (walking, biking, and public transportation) to better understand their relationships. A mixed‐methods was used, combining an online quantitative survey (N = 538, 54.83% women), three focus groups (n = 9, three women), and eight individual interviews (five women). The survey examined the facilitators and barriers of active and sustainable mobility (ASM) during a typical week using multiple linear regressions. Interviews and focus groups, with mobility experts and car users wishing to reduce their car use in Grenoble, were analyzed through reflexive thematic analysis. Quantitative results highlighted socio‐spatial (e.g., having a transport pass, trip chain complexity), sociodemographic (having children aged 12+), and individual factors (e.g., car attitudes, ASM habits, perceived health) as independent predictors of ASM. Moreover, intention toward ASM interacted with bike and transport pass ownership. Qualitative findings revealed local‐specific factors, such as living in mountainous areas. These results emphasize the importance of studying behavior as part of a complex system with multi‐level factors and using both national and local samples to better identify facilitators and barriers.

## INTRODUCTION

Promoting active and sustainable mobility (ASM) while reducing car use has the potential to improve personal health and mitigate climate change. ASM refers to transport modes that rely on human physical activity (e.g., walking, cycling, kick scootering, using public transport) embedded within a mobility system, minimizing environmental impacts, promoting social equity, and ensuring long‐term accessibility and health benefits (Banister, 2008; WHO, 2018a). On the one hand, ASM is associated with increased physical activity (Chaix et al., 2019; Pucher et al., 2010; Rojas‐Rueda et al., 2011; Wanner et al., 2012), life expectancy (Cepeda et al., 2017), and well‐being (Martin et al., 2014). It is also a key public strategy for reducing air pollution and addressing climate change (Bernard et al., 2021; Brand et al., 2021). On the other hand, motorized transport presents both public health and environmental concerns. Indeed, regular car use is linked to physical inactivity, sedentary behavior, and a heightened risk of obesity and overweight (Chakrabarti & Shin, 2017; Sugiyama et al., 2012). It is also a major contributor to greenhouse gas emissions and fine particulate pollution, both of which are known to exacerbate cardiovascular and respiratory diseases (WHO, 2018b).

Despite the co‐benefits of ASM, and the co‐damages of motorized individual transport, car use remains the dominant mode of transport. A study conducted across 800 cities and 61 countries found that, in both Northern and Southern Europe, 50% to 75% of daily mobility is car‐based (Prieto‐Curiel & Ospina, 2024). In addition, ASM remains underutilized for daily travel. In France, for example, only 37% of daily trips involve ASM (SDES, 2020). Accordingly, there is an urgent need to identify the barriers and levers of travel mode choices to design interventions that both reduce car use and promote ASM.

### Individual Factors of Daily Mobility

At the individual level (person‐specific), several psychological and behavioral factors of travel mode choices have been identified. Socio‐cognitive models of behavior change in psychology consider that intervention strategies should primarily target psychological variables, and more particularly perceptions and beliefs, regarded as the most proximal and modifiable factors of behaviors (Bartholomew et al., 2016; Michie et al., 2011). The Theory of Planned Behavior (Ajzen, 1985, 1991) and the Value‐Belief‐Norm Model (Stern et al., 1999) are among the most widely applied socio‐cognitive models in the psychology of travel modes literature (Javaid et al., 2020).

The Theory of Planned Behavior assumes that behavioral adoption depends on intention to do so, which itself relies on three core factors: perceptions of one's capability to adopt the behavior (*perceived behavioral control* or *self‐efficacy*), perceptions about the benefits and costs of the behavior (*attitudes*), and social influences, encompassing perceptions of others' behaviors (*descriptive norms*) and normative beliefs (*subjective norms*). The review of reviews by Javaid et al. (2020) provides support to this approach by highlighting the consistent role of these variables in shaping travel mode choices. Empirical research notably shows that individuals with higher self‐efficacy and greater knowledge of transport systems (or *mobility capital*, Kaufmann et al., 2004) are more likely to engage in ASM (Hoffmann et al., 2017; Lanzini & Khan, 2017). Attitudes may also play a role in travel mode choices (McCarthy et al., 2021; Steg, 2005; Vincent‐Geslin, 2010): car use is motivated not only by instrumental functions (e.g., comfort, speed) but also by its symbolic and affective meanings such as autonomy or freedom (Steg, 2005). In the same vein, Vincent‐Geslin (2010) found that individuals who shifted from car use to ASM often experienced a “break‐up” phase, marked by discomfort or constraints (e.g., traffic jams, limited driving ability), which—combined with external factors like increased costs, new transport options, or environmental engagement—triggered behavior change.

The Value‐Belief‐Norm Model explains pro‐environmental behavior as a causal chain linking values, beliefs, and personal norms. Applied to travel mode choices, it posits that awareness of the environmental consequences of travel modes, combined with a sense of personal responsibility, can activate a moral sense of obligation to act (*personal norms*) that promote, in turn, ASM choices. This model, too, has received empirical support, several studies highlighting the central role of moral and environmental norms in shaping travel mode choices (Huang et al., 2024; Javaid et al., 2020).

Although socio‐cognitive models are dominant in the psychology of travel modes literature, other research based on dual‐process models (Strack & Deutsch, 2004) goes beyond these rational choice models, by underscoring the role of automatic processes, such as habits, in shaping travel mode choices (Schoenau & Müller, 2017). Habits—defined here as automatic behaviors triggered by environmental cues—play a critical role in travel mode choices (Rejeb et al., 2023). Given the repetitive nature of daily mobility (e.g., commuting to work at the same time via the same route), habits are likely to be both formed and reinforced over time (Klöckner & Matthies, 2004). Javaid et al.’s (2020) synthesis of reviews confirms the importance of habits as a predictor of travel mode choices.

Finally, other variables at the individual level seem to shape travel mode choices. Notably, people who are physically active on a regular basis seem to be more prone to adopt ASM than usually inactive people (Makkonen et al., 2024), as well as people with better health outcomes, such as health behaviors and perceived health (Bopp et al., 2013).

### Socio‐spatial and Sociodemographic Factors of Daily Mobility

Although several psychological and behavioral factors of travel mode choices have been identified, mobility is a complex phenomenon shaped not only by individual factors but also by a wide range of contextual influences (Huang et al., 2024; Javaid et al., 2020). Research in geography and urban planning notably shows that travel mode choices may be particularly sensitive to socio‐spatial factors (i.e., social and spatial characteristics of the environment) (Van Acker et al., 2010). For example, daily travel mode choices correlate with population urban density and workplace concentration (Oakes et al., 2007), destination accessibility (Nielsen & Skov‐Petersen, 2018), travel distances, land‐use diversity, and urban design (De Witte et al., 2013; Ewing & Cervero, 2010; Leslie & Cerin, 2008; Panter et al., 2013; Wang et al., 2016). Individuals in high‐density, well‐connected areas tend to rely less on cars and more on ASM (Clark et al., 2016). Further determinants include trip purposes (e.g., commuting, leisure), trip chaining complexity, access to transport resources (e.g., bike ownership, transit pass), and mobility biographies (i.e., transport experiences and habits formed during early life stages that may shape later behaviors) (Clark et al., 2016; De Witte et al., 2013; Kim & Gulfarsson, 2008; Martin et al., 2012; Müggenburg et al., 2015). Specifically, individuals with prior active mobility experience, simplified trip chaining, and access to sustainable transport options exhibit lower car dependency (De Witte et al., 2013; Van Acker et al., 2019). Factors related to transport accessibility and mobility are defined in this article as “mobility‐related.”

Sociodemographic factors (i.e., social and demographic characteristics of individuals) such as age, gender, education, employment status, household size, and the presence of young children may also affect travel mode choices (Bouscasse et al., 2018; de Geus et al., 2019; Pucher et al., 2010; Rubin et al., 2014). However, the evidence base remains limited, as these variables are most often treated as control variables rather than examined in depth (Javaid et al., 2020). Moreover, findings remain inconsistent (Huang et al., 2024; Javaid et al., 2020): while some studies suggest that older individuals exhibit greater car reliance than younger ones, others report no significant age patterns (De Witte et al., 2013). Similarly, the effects of socioeconomic status on travel mode choices are heterogeneous across studies (Huang et al., 2024). Gender differences in travel mode choices are also debated: some studies report that women display higher levels of multimodality than men (i.e., using a broader range of transport modes) due to more complex activity patterns and family responsibilities (e.g., Heinen & Chatterjee, 2015), whereas other research indicates that these constraints act as barriers to multimodality (An et al., 2021).

### Toward a better integration of multiple‐level factors

In sum, studies from different disciplines highlight that determinants of travel mode choices operate at different levels, underscoring the complexity of the phenomenon. However, despite growing interest in multidisciplinary approaches to travel mode choices (Van Acker et al., 2010), most research has adopted a mono‐disciplinary perspective, and few empirical studies have strived to integrate factors at the individual, sociodemographic, and socio‐spatial levels into a unified framework (but see reviews by Huang et al., 2024; Javaid et al., 2020). As a result, the nature of the relations between these factors—whether they act independently or through mediation or moderation links—remains underexplored.

On the one hand, based on the *sufficiency hypothesis* (Ajzen, 2011), one may expect the influence of background (i.e., socio‐spatial and sociodemographic) variables on travel mode choices to be mediated by psychological constructs. According to this hypothesis, socio‐cognitive variables (notably intention and self‐efficacy) are the most proximal factors of behaviors, and all other variables affect behavior through them. In this perspective, the effects of background variables are fully driven by psychological ones. In support of this hypothesis, Klöckner and Blöbaum (2010) found that self‐efficacy mediates the link between car access and travel mode choices, while Giles‐Corti et al. (2016) demonstrated that urban planning factors (e.g., infrastructure, density of population, and services) influence travel mode choices through social norms and personal attitudes. The review of reviews conducted by Javaid et al. (2020) also reveals that traffic infrastructure impacts self‐efficacy and habit formation, which, in turn, affect travel mode choices.

On the other hand, other research suggests the possibility of independent or moderating relations between factors operating at different levels. Javaid et al. (2020) reported direct effects on travel mode choices not only of individual factors, but also of sociodemographic and socio‐spatial ones. However, as this is a synthesis of reviews, it remains unclear whether these factors were examined simultaneously within individual empirical studies. In addition to possible independent effects, Javaid et al. (2020) suggest that the effects of variables at a particular level may depend on variables at other levels, pointing to potential moderating relationships. For example, the effects of transport price on travel mode choices differ between women and men (Hensher, 2008), and highly depend on infrastructure availability (Avner et al., 2014). Furthermore, how people evaluate transport modes may depend on context. For example, Hoffmann et al. (2018) showed that such perceptions varied with trip length, journey purpose, and mode type, with car users emphasizing flexibility for short trips, and non‐car users valuing environmental and health aspects more. Conversely, psychological factors may also moderate the effects of urban environment on travel choices, but effects are inconsistent (Rhodes et al., 2018), and research remains scarce.

### The Present Study

To address these gaps, we tested three concurrent hypotheses regarding the relations between individual (psychological and behavioral), sociodemographic and socio‐spatial variables (See Supplementary Figure 1 and Supplementary Figure 2 summarizing the hypotheses and Supplementary Table S1 for the full list of variables). The study focused on factors whose influence on travel mode choices has been documented in previous research, such as socio‐cognitive constructs from the Theory of Planned Behavior (intention, self‐efficacy, attitudes, subjective norms), the Value‐Belief‐Norm model (green identity), and the dual‐process approach (habits), usual physical activity, and health‐related perceptions (e.g., perceived risk of contracting Covid‐19, as the study was conducted during the sanitary crisis) (psychological and individual factors); gender, age, socioeconomic status, and household composition (sociodemographic factors); and urban density, trip purposes, trip chaining complexity, access to transport resources, and mobility biographies (socio‐spatial factors).

Specifically, we tested if these variables are independently associated with ASM (H1), if psychological factors (intention and self‐efficacy) mediate the associations between the other variables and ASM (H2), or if psychological factors moderate (H3) the associations between the other variables and ASM. Results supporting H2 but not H1 would be in line with the sufficiency hypothesis (Ajzen, 2011), which posits that psychological factors fully mediate the influence of other variables on ASM. Conversely, results supporting H1 or H3 would indicate a more complex pattern: either direct effects of variables from multiple levels (H1), or interactions showing that the associations between psychological factors and ASM depend on contextual factors. More particularly, for each group of variables, we focused on those for which prior research provides evidence of associations with travel mode choices.

These three concurrent hypotheses were examined in a cross‐sectional quantitative study. To have a finer‐grained understanding of socio‐spatial factors specific to the context of the study, we further explored these factors in a qualitative study. Indeed, transport geography requires a deep understanding of the context in which daily mobility takes place (Van Acker et al., 2010). Using a mixed‐methods design combines the strengths of quantitative and qualitative approaches to provide both generalizable patterns and rich, contextualized insights (Patton, 1990). While the quantitative study enables us to identify the nature of the associations (i.e., independent, mediating, moderating) between the variables of interest across a broad population sample, the qualitative study allows us to examine how these factors operate in specific settings and from the perspectives of key actors, such as mobility stakeholders and residents. Such a qualitative approach may allow for an in‐depth understanding of contextual and experiential dimensions of travel mode choices that might be overlooked in quantitative studies that are based on general theories (e.g., Beirão & Cabral, 2007). Moreover, as highlighted by Aldred (2019), even when individuals express preferences for built environment features conducive to active travel—such as wide footpaths or protected cycling infrastructure—the broader cultural context for sustainable mobility can vary substantially across regions and shape behavioral change. Such an approach can reveal place‐based, situated meanings and constraints that shape travel mode choices and behaviors, particularly in areas where geographic, infrastructural, and policy conditions interact in unique ways.

Specifically, the qualitative study was conducted in the Grenoble Metropolitan Area (France), a region with unique geographic and policy characteristics. Although Grenoble is known for its ambitious sustainable transport policies: early adoption of urban tramway systems, extensive cycling infrastructure (450 km of bike lanes), and ambitious sustainable transport policies, the region also faces important mobility challenges due to its topographical metropolitan constraints, as it is situated in the Alps (Chardonnel et al., 2018). These features make Grenoble a relevant case study for exploring the perceived barriers and facilitators to ASM in a complex geographic context.

## METHODS

### Participants

#### Quantitative study: Online questionnaire

Before starting the study, we carried out an a priori power analysis using G. Power 3.1.9.4 (Erdfelder et al., 2009). This analysis indicated the need for 378 participants to obtain an R^2^ of 0.60 (obtained in Schoenau & Müller, 2017) with a power of 90% while considering 72 predictors (36 independent predictors and 36 interactions). In total, 671 individuals completed the online questionnaire. The condition for participating in the study was to be major and live in Metropolitan France. After applying the inclusion criteria (being over 18 years old, having provided informed consent, and having completed all questionnaire items), 538 participants were retained for analysis (for more details, see Supplementary Tables S1 and S2).

#### Qualitative study: Focus groups and Individual Interviews

Twelve experts (five women and seven men, aged between 21 and 60 years) with diverse profiles (e.g., researchers, policy‐makers, urbanists and consultants working in mobility in Grenoble Metropolitan area) and five car drivers (three women, aged between 21 and 70 years) intending to reduce car use (living or working in Grenoble Metropolitan Area) participated in this study (see Supplementary Table S3). Experts were recruited through purposive sampling, by identifying relevant profiles on professional social networks or through suggestions from the research team, and were approached by email. Individuals intending to reduce car use were recruited through convenience and snowball sampling, using the contact list of a pilot study on mobility behavior change and participant referrals, and were contacted by telephone. Two additional experts were contacted by email, but did not respond. Selecting both mobility experts and car users intending to reduce their car use was a deliberate methodological choice. Experts provide system‐level insights into structural and policy‐related determinants, whereas car users offer first‐hand accounts of individual motivations and constraints. In line with best practices in behavior‐change research, conducting a needs assessment requires integrating perspectives from both potential intervention beneficiaries and domain experts (Bartholomew et al., 2016). This combination was therefore selected to capture complementary inputs relevant for designing effective behavior‐change strategies.

Data were collected either online via videoconference (for experts and two of the car drivers) or face‐to‐face at the University Grenoble Alpes (for the remaining car drivers and experts). During the focus groups, in addition to the moderator (CTE), two observers were present to take detailed field notes to complement the transcripts (in addition to video‐registration that was made at the same time).

### Procedure and measures

#### Quantitative study

The participants of the quantitative study completed a 20‐minute survey in October 2020. The first part of the survey included an online informed consent that was compulsory to read and accept in order to continue the survey. Then they answered items about individual factors associated with ASM (e.g., intention toward ASM, subjective norms, ecological identity), mobility‐related factors (e.g., possession of a bike, trip chaining, car accessibility), and sociodemographic information (e.g., gender, age, level of incomes). Two ASM outcomes were considered separately, according to the responders' typical week uses: the percentage of trips made by biking or walking, and the percentage of trips made by public transport or carpooling. For a full description of the 36 variables measured and the tools used, see Supplementary Table S1 and Supplementary material File 3. All scales showed good reliability (⍺s > .60).

#### Qualitative study

The interviews and the focus groups were conducted by CTE, a woman PhD student in psychology at the time of the study with a Master of Science degree in psychology. Prior to this project, she had attended a training course on qualitative research methods and had previous experience in data collection during her bachelor's studies. Before starting the focus groups or the individual interviews, all participants signed informed consent and an authorization to be filmed.

A semi‐structured interview guide was followed during both the individual interviews and focus groups. This guide contained predefined questions and prompts designed to elicit detailed accounts of participants' experiences. The development of the interview guide was informed by the PRECEDE‐PROCEED model, which guided the exploration of predisposing, enabling, and reinforcing factors related to behaviour change (Green & Kreuter, 2005). Before data collection, the questions were piloted with colleagues of the interviewer to gather feedback and refine wording and clarity. The protocol included questions about participants' profiles (e.g., age, profession) and any past experiences with the promotion or adoption of ASM. The second part focused on potential levers (ideas, thoughts, resources) that might facilitate the adoption of ASM, as well as obstacles (ideas, resources, contexts) that might hinder such behaviors. The complete interview guide is provided in the Supplementary material File 5.

Among experts, eight of them participated in individual interviews, and four participated in a focus group. Five individuals intending to reduce car use and living or working in the Grenoble Metropolitan Area took part in two additional focus groups. Each participant was interviewed only once. In these groups, participants first introduced themselves (profession) and described their past experiences in changing daily travel mode choices (from car to another mode). The second part of the discussion followed the same structure as for the expert participants.

All interviews and focus groups were video‐recorded. In addition, as already explained, during the focus groups, two observers were present to take notes in parallel with the recordings. The average duration of each interview and focus group is reported in the Supplementary material File 4. Data saturation was not explicitly discussed, and the transcripts were not returned to participants for further comment or correction.

The interviewer (CTE) had no prior personal relationship with the experts. Among the participants aiming to change their travel mode choices, two were colleagues from the same institution as the interviewer, while the others were not previously known to her. All participants were aware that the interviewer was a PhD student conducting a thesis on motivations and barriers to reducing car use. For participants not personally known to her, no additional information about the interviewer was available, whereas the two colleagues were familiar with the general purpose of the research.

### Analytical procedures

#### Quantitative study

In the quantitative study, we checked the normality of the dependent variables (percentage of biking/walking and percentage of public transport/carpooling) using skewness and kurtosis indices. These analyses showed that this percentage was normally distributed.

Hypotheses H1 to H3 were tested using multiple linear regressions in R studio version 4.1.2, using the function “Lm” for H1 and H2, and a stepwise regression with the “olsrr” package (Hebbali, 2020) for H3. Moreover, dummy variables were created for the categorical variables (gender and principal motive of commuting). The reference group for gender was women, and the reference group for the principal motive of commuting was work/studies.

H1 was tested using a multiple linear regression analysis, following the steps suggested by Sniehotta et al. (2013) and Teran‐Escobar et al. (2021). In the first step, we investigated the association between all the mobility‐related and sociodemographic variables and ASM. In the second step, we added the individual variables. Finally, we compared the explanatory power of both models by using a chi‐square difference test.

H2 was tested using a mediation analysis based on the recommendations of Yzerbyt et al. (2018). The joint significance test was used to diminish the probability of a Type I error, as it presents a better balance between Type I error and statistical power compared to other mediation approaches such as bias‐corrected bootstrap method. In the first step, we tested the association between mobility‐related, sociodemographic and individual variables and biking/walking and using public transport. In the second step, we investigated the association between the mobility‐related, sociodemographic and individual variables, and the hypothesized mediators (intention and self‐efficacy toward ASM). In the third step, we tested the association between the mobility‐related, sociodemographic and individual variables, including the potential mediators, and biking/walking and using public transport. If the regression coefficients in the second and third steps were significant, we tested a possible indirect effect by using Montecarlo joint analysis (i.e., JS mediation package, Batailler et al., 2021).

H3 was investigated by using a stepwise forward regression analysis. First, we centered all the independent variables (i.e. subtract mean) to avoid any multicollinearity problems (Iacobucci et al., 2016; Shieh, 2011). Second, we tested the association between mobility‐related, sociodemographic, and individual factors and biking/walking and using public transport. Third, we tested the association between mobility‐related, sociodemographic, and individual factors and the interaction between mobility‐related and sociodemographic factors and intention toward ASM (e.g., number of children under 12 × intention toward ASM) and biking/walking and using public transport. Finally, the significant interactions were decomposed into simple slopes and Johnson‐Newman plots.

Moreover, after each hypothesis was tested, we assessed the linearity, homogeneity of variance, collinearity and the normality of residuals by using the “performance” package (Lüdecke et al., 2021) (see Supplementary Figure 3, Supplementary Figure 4 and Supplementary Figure 5).

The R code and the dataset for this research are available in the open platform OSFHOME (https://osf.io/9h2ck/?view_only=88ea796fe6574b8c9bed7ade6936543c).

#### Qualitative study

To analyze and interpret the qualitative data, we used a deductive reflexive thematic analysis (Braun & Clarke, 2012, 2021). This method allows the identification, organization, and interpretation of patterns of themes across datasets. The analysis was informed by the first author's theoretical background in health and environmental psychology, which sensitized the analysis to potential levers and obstacles to active and sustainable mobility. This distinction functioned as an analytic orientation rather than a predefined coding framework. Coding and theme development were conducted flexibly and iteratively through reflexive engagement with the data.

The analysis was conducted using NVivo 11 (QSR International Pty Ltd, 2015) by CTE and a research intern, who was trained in the use of thematic analysis. The research assistant contributed to the qualitative analysis by participating in the coding process and in reflexive discussions that informed the refinement of the themes. The final interpretation, theoretical integration, and manuscript writing were conducted by the author team. The steps followed were adapted from Braun and Clarke (2012):

1. Both researchers read the corpus to familiarize themselves with the material.
2. They generated initial codes or “nodes” by noting patterns of meaning relevant to the research questions (e.g. *“biking can be dangerous because of the car drivers' incivilities”*, *“using public transport in the evening can be dangerous”*)
3. They developed candidate themes capturing broader patterns of shared meaning across the dataset (e.g., “the lack of security while commuting can be an obstacle for some people”)
4. They reflexively examined the coherence of each theme and the distinctions between themes, ensuring that each theme captured a meaningful and coherent pattern in the data.
5. Themes were collaboratively reviewed and iteratively refined, then clearly defined and named to capture their central organising concept.

No coding tree was developed beyond the distinction between levers and obstacles, which guided the thematic organization. Transcripts or themes were not returned to participants for feedback.

## RESULTS

### Descriptive statistics

#### Quantitative study

We recruited participants living in France and aged 18 and more through social media (i.e., Twitter and Facebook) and by word of mouth (*N =* 538, 54.83% women, *M*_age_ = 38, *SD* = 11.24, 83% of the participants had at least a Master's degree and 72% had a household net income of at least 2000 euros per month) (for more details on the variables see Supplementary Table S2). The sample reported an average percentage of 49% (SD = 42.77) of their trips made by biking (classic or electric) or walking, and 22.14% (SD = 34.69) made by public transport or carpooling. Public transport refers here to collective and scheduled passenger services (e.g., bus, tramway, train, metro) (Vuchic, 2005). Table 1 shows the means, standard deviations and description of all variables. Correlations between the variables can be found in Supplementary Table S2.

**TABLE 1:** Means, standard deviations, confidence intervals, and description of variables.

Variable	Mean	SD	Median	Min ‐ Max	Range/unity of measure
**Dependent variable**					
Percentage of biking or walking during a typical week	49.00	42.77	50.00	0.00–100.00	0–100%
Percentage of using public transport or car‐sharing during a typical week	22.14	34.69	0.00		
**Sociodemographic and mobility‐related variables**					
Gender	54.83% women, 44.61% men, 0.56% NA				
Age	38	11.24	36	19–69	
Educational attainment	5.98	1.03	6.00	2–7	0–7
Level of incomes	4.40	1.41	5.00	1–6	1–6
Work percentage	93.18	16.97	100.00	1–100	0–100%
Number of persons in the household	2.50	1.32	2.00	1–4	
Number of children under 12 years	0.44	0.84	0.00	0–7	
Number of children of 12 years and older	0.35	0.84	0.00	0–9	
Habitat surface	86.00	47.17	75.00	1–300	Square meters
Number of cars in the household	1.21	0.98	1.00	0–6	
Possession of a bike	3.19	1.56	4.00	0–4	0–4
Possession of a transport pass	1.78	1.24	1.00	1–4	1–4
Density of the domicile	8707.1	11485.11	4921.4	0–91852.7	Pers/km2 ratio
Accessibility by car	2.57	0.81	3.00	1–3	1–3
Proximity to a public transport stop	2.71	0.63	3.00	1–3	1–3
Principal motive of commuting	89.78% work or study, 4.09%, purchases and other services, 2.05% accompany someone, 4.09% “other” such as sports or cultural activities, volunteering				
Frequency of simple trip chaining (2 activities)	2.75	0.83	3.00	1–4	1–4
Frequency of medium trip chaining (3 or 4 activities)	1.93	0.81	2.00	1–4	1–4
Frequency of complex trip chaining (chaining 5 or more activities in the same travel)	1.36	0.61	1.00	1–4	1–4
ASM during elementary school	2.31	0.91	3.00	1–3	1–3
ASM during high school	2.64	0.63	3.00	1–3	1–3
ASM during university	2.65	0.69	3.00	1–3	1–3
ASM during first job	2.41	0.87	3.00	1–3	1–3
**Psychological and Individual Variables**					
Intention toward ASM	4.89	2.42	6.0	1–7	1–7
Self‐efficacy toward ASM	4.92	2.41	6.0	1–7	1–7
Attitude toward car	3.12	1.96	2.5	1–7	1–7
Attitude toward ASM	4.98	1.88	5.5	1–7	1–7
Social norms of ASM	3.72	1.29	3.6	1.2–7	1–7
Car habits	2.30	1.77	1.25	1–7	1–7
ASM habits	4.51	2.22	5.0	1–7	1–7
Associated ASM habits	2.45	0.90	2.33	1–5	1–5
Green identity	5.97	0.98	6.2	1.6–7	1–7
Perceived risks of getting COVID‐19	2.91	1.20	2.80	1–6.8	1–7
Moderate‐to‐vigorous PA	513.1	488.46	420.0	1–4,640	Minutes per week
Perceived physical health	3.29	0.34	3.20	1.8–5.0	1–5

*Note*: N = 538. ASM = Active and sustainable mobility, PA = Physical activity, CI = Confidence interval, SD = Standard deviation, NA = No answer, Pers/km2 ratio = Person per squared kilometer ratio. Values between brackets represent confidence intervals.

#### Qualitative Study

The profile of the participants of the focus groups and the interviews is described in the Supplementary Table S3. Eight participants (47%) were women and 12 were men (53%); the participants were aged between 21 and 70 years old.

### The independent associations between mobility‐related, sociodemographic, and individual factors with biking and walking (H1)

The Model 2a (Supplementary Table S4) including all the mobility‐related, sociodemographic, and individual variables predicting biking or walking was statistically significant (*F* [37, 389] = 16.74, *p* < .001), with a *R* ^2^ = .58 (compared to the *R* ^2^ of the Model 1a of .31). Among the sociodemographic variables, only the number of children aged 12 years and older in the household was negatively associated with biking and walking for commuting (β = −.10, *p* = .025). Concerning mobility‐related factors, possession of a bike (β = .08, *p* = .047), of a transport pass (β = −.23, *p* < .001), and accessibility by car (β = .07, *p* = .041), as well as medium trip chaining (β = −.12, *p* = .023) and complex trip chaining (β = .10, *p* = .026), were significantly associated with biking and walking for commuting. Finally, regarding individual factors, perceived physical health (β = .08, p = .027), negative attitude toward car use (β = −.33, *p* < .001), positive attitude toward ASM (β = .17, *p* = .001), car‐use habits (β = −.14, *p* = .007), ASM habits (β = .10, *p* = .042), and associated ASM habits (e.g., reading, listening to the radio during commuting) (β = −.18, *p* < .001) were also significantly linked with biking and walking for commuting. Detailed results are presented in Supplementary Table S4. Referring to the chi‐squared distribution, the critical value for 12 degrees of freedom (corresponding to the difference in the number of parameters between Model 2 and Model 1) was 21.03. The likelihood ratio test statistic, based on the difference in log‐likelihoods, was 223.36. Since this value exceeds the critical threshold, the test indicates that the more complex model (Model 2a) provides a significantly better fit to the data than the simpler model (Model 1a).

### The independent associations between mobility‐related, sociodemographic, and individual factors in using public transport (H1)

The Model 2b including all the mobility‐related, sociodemographic, and individual variables predicting using public transport was statistically significant (*F* [37, 389] = 11.86, *p* < .001), with a *R* ^2^ = .49 (compared to the *R* ^2^ of the Model 1b of .44). Among the mobility‐related factors, possession of a transport pass (β = .53, *p* < .001), medium trip chaining (β = .12, *p* = .045), and complex trip chaining (β = −.14, *p* = .004) were significantly associated with using public transport and/or carpooling. Regarding mobility biographies, having practiced ASM during the first job was positively associated with using public transport and/or carpooling (β = .10, *p* = .026). Finally, concerning psychological variables, intention toward ASM (β = .11, *p* = .038), negative attitudes toward ASM (β = −.13, *p* = .028), and ASM habits (β = .22, *p* < .001) were also significantly associated with using public transport and/or carpooling. Detailed results are presented in Supplementary Table S5. Referring to the chi‐squared distribution, the critical value for 12 degrees of freedom (corresponding to the difference in the number of parameters between Model 2 and Model 1) was 21.03. The likelihood ratio test statistic, based on the difference in log‐likelihoods, was 51.83. Since this value exceeds the critical threshold, the test indicates that the more complex model (Model 2b) provides a significantly better fit to the data than the simpler model (Model 1b).

### The mediation of the associations between mobility‐related, sociodemographic, and individual factors with ASM, by intention and self‐efficacy (H2)

Hierarchical multiple regression analyses were used (see Supplementary Table S6). Models 3a and 3b tested the association between mobility‐related, sociodemographic, and individual variables (except the potential mediators), and either biking and walking (Model 3a) or using public transport (Model 3b). Model 3a was statistically significant (*F* [35, 391] = 17.48, *p* < .001), with an *R* ^2^ of .58. The association between the number of children of 12 years and older (β = −.10, *p* = .035), possession of a bike (β = .08, *p* = .046), possession of a transport pass (β = −.22, *p* < .001), accessibility by car (β = .07, *p* = .048), frequency of medium trip chaining (β = −.12, *p* = .026), frequency of complex trip chaining (β = .09, *p* = .034), perceived physical health (β = .08, *p* = .020), attitude toward car (β = −.35, *p* < .001), attitude toward ASM (β = .23, *p* < .001), car habits (β = −.14, *p* = .005), ASM habits (β = .11, *p* = .018), and associated ASM habits (β = −.18, *p* < .001) were statistically significant predictors of biking.

Model 3b was statistically significant (*F* [35, 391] = 12.32, *p* < .001), with an *R* ^2^ of .48. The association between possession of a transport pass (β = .54, *p* < .001), frequency of medium trip chaining (β = .12, *p* = .034), frequency of complex trip chaining (β = −.15, *p* = .002), ASM during first job (β = .10, *p* = .017), and associated ASM habits (β = .22, *p* < .001) was statistically significant predictors of public transport use.

Model 3.2 tested the association between the sociodemographic, mobility‐related, and individual variables, and intention toward ASM. This model was statistically significant (*F* [35, 391] = 14.92, *p* < .001) with an *R* ^2^ of .53. The association between possession of a bike (β = .09, p = .027), possession of a transport pass (β = .08, p = .049), accessibility by car (β = .09, p = .020), the principal motive “other” (β = −.08, p = .021), attitude toward car (β = −.12, p = .034), attitude toward ASM (β = .47, p < .001), ASM habits (β = .15, p = .003), and green identity (β = −.09, p = .017) were statistically significant predictors of intention toward ASM.

Model 3.3 tested the association between the sociodemographic, mobility‐related, and individual variables, and self‐efficacy toward ASM. This model was statistically significant (*F* [35, 391] = 18.64, *p* < .001) with an *R* ^2^ of .59. The association between the number of persons in the household (β = −.19, *p* = .008), the number of children under 12 (β = .12, *p* = .035), attitude toward car (β = −.19, *p* < .001), attitude toward ASM (β = .49, *p* < .001), and ASM habits (β = .12, *p* = .007) was statistically significant predictors of self‐efficacy toward ASM.

Finally, the models testing the association between all the mobility‐related, sociodemographic, and individual factors and biking and/or walking (Model 1b) and using public transport (Model 2b) were already presented (Supplementary Tables S4 and S5). As intention and self‐efficacy were not significantly associated with biking/walking, the conditions for mediation were not met. Therefore, no indirect effects were tested for this outcome (percentage of travel made by biking/walking).

Then, we followed the steps recommended by Yzerbyt et al. (2018) by testing the indirect effects of bike possession, transport pass possession, car accessibility, and the principal motive “other” on public transport use through intention. As self‐efficacy was not significantly associated with public transport, no indirect effects were tested for this mediator.

Using public transport was significantly mediated by intention (Indirect effect = 1.59; CI 95% [0.92; 2.35]), and the association between the possession of a transport pass and using public transport was significantly mediated by intention toward ASM (Indirect effect = 0.53; CI 95% [0.15; 1.00]) (see Supplementary Table S7).

### The moderation of the associations between mobility‐related and sociodemographic factors with ASM by intention (Hypothesis 3)

**FIGURE 1:**
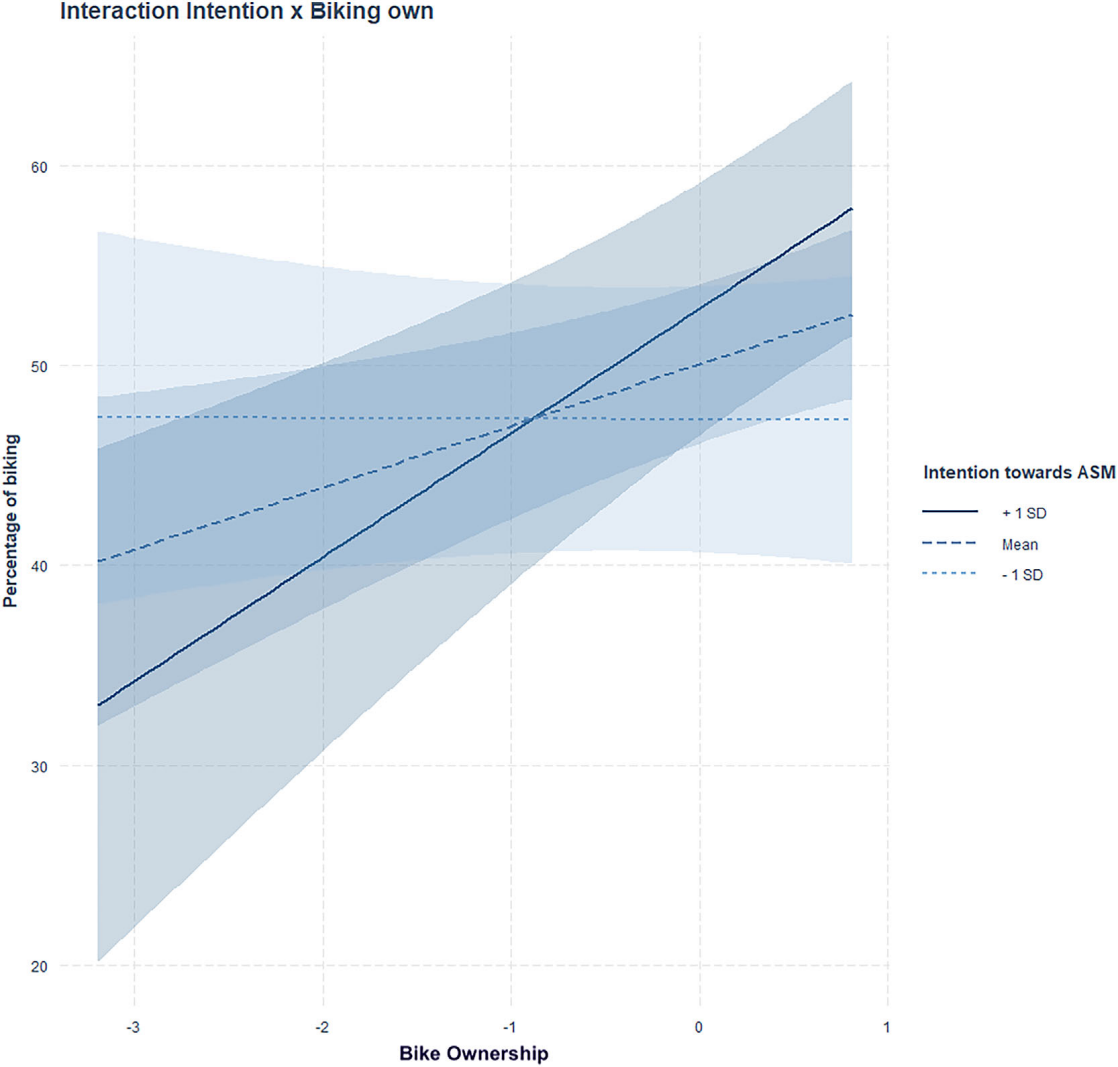
Interaction between bike ownership and intention toward ASM on biking/walking.

**FIGURE 2:**
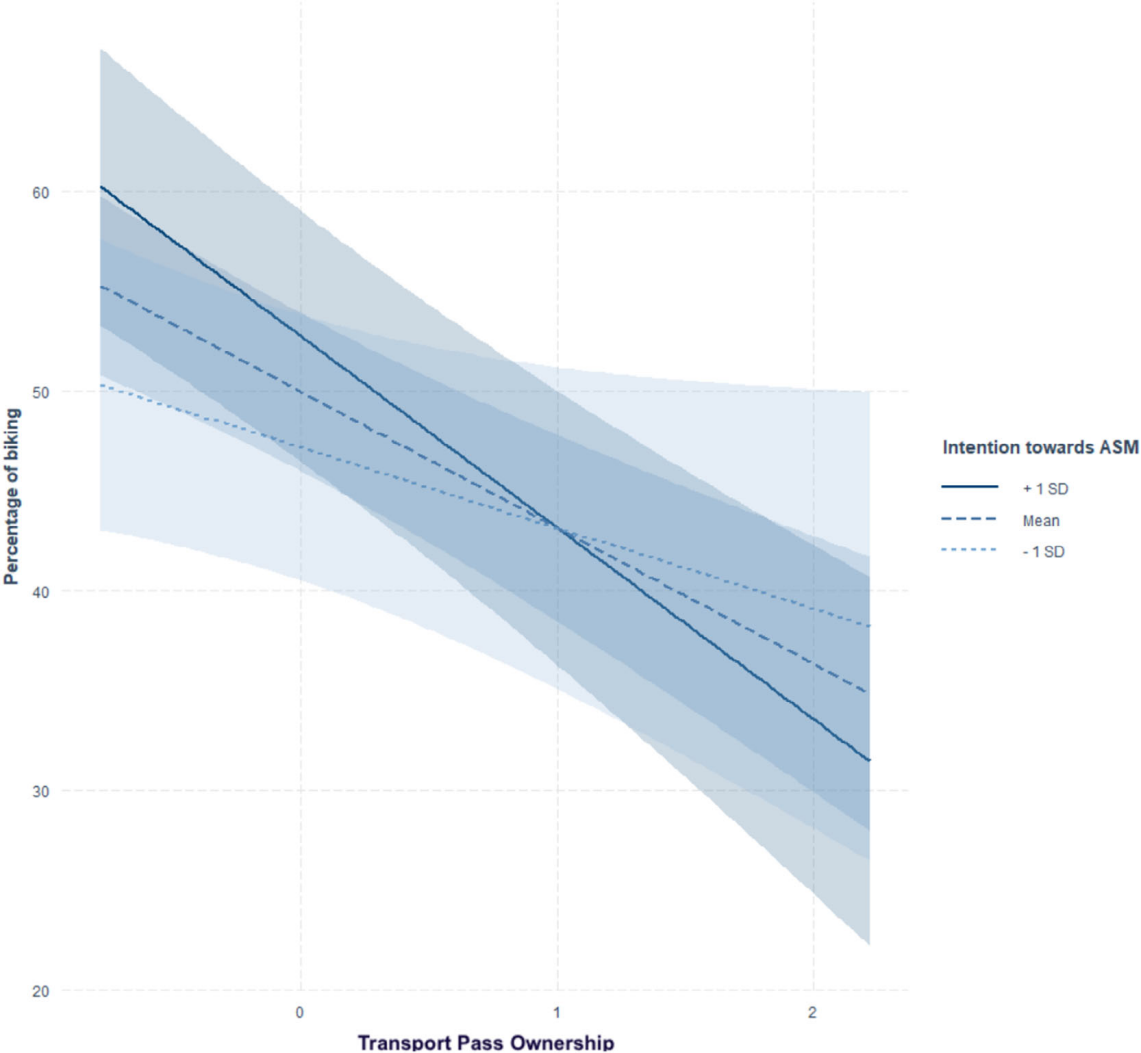
Interaction between public transport pass ownership and intention toward ASM on biking/walking.

A stepwise regression analysis including all the variables and the interactions between intention toward ASM and mobility‐related and sociodemographic factors was used (Supplementary Tables S8 and S9). Model 4a was statistically significant (*F* [46, 380] = 14.97, *p* < .001), with an *R* ^2^ of .76. The association between attitude toward the car (β = −0.31, *p* < .001), possession of a transport pass (β = −0.19, *p* < .001), attitude toward ASM (β = 0.20, *p* < .001), associated ASM habits (β = −0.16, *p* < .001), possession of a bike (β = 0.11, *p* = .007), frequency of complex trip chaining (β = 0.09, *p* = .037), ASM habits (β = −0.13, *p* = .008), and biking/walking was significant. We also found significant interaction effects between possession of a bike × intention toward ASM (β = 0.12, *p* = .001), and between possession of a transport pass × intention toward ASM (β = −0.07, *p* = .048), on biking/walking (see Figures 1 and 2). Simple slope analyses (see Supplementary Table S10) showed that individuals possessing a bike traveled more often by biking and walking but only if they presented average (b = 3.09, *p* = .01) or stronger intentions (b = 6.21, *p* < .001) compared to individuals with low intentions toward ASM (b = 0.03, *p* = .980). Concerning the interaction between possessing a public transport pass and intention on biking, slope analysis (see Supplementary Table S11) showed that individuals possessing a public transport pass travelled less often by biking or walking but only if they presented average (b = −6.82, *p* < .001) or stronger intentions (b = −9.60, *p* < .001) compared to individuals with low intentions toward ASM (b = −4.04, *p* = .060).

**FIGURE 3:**
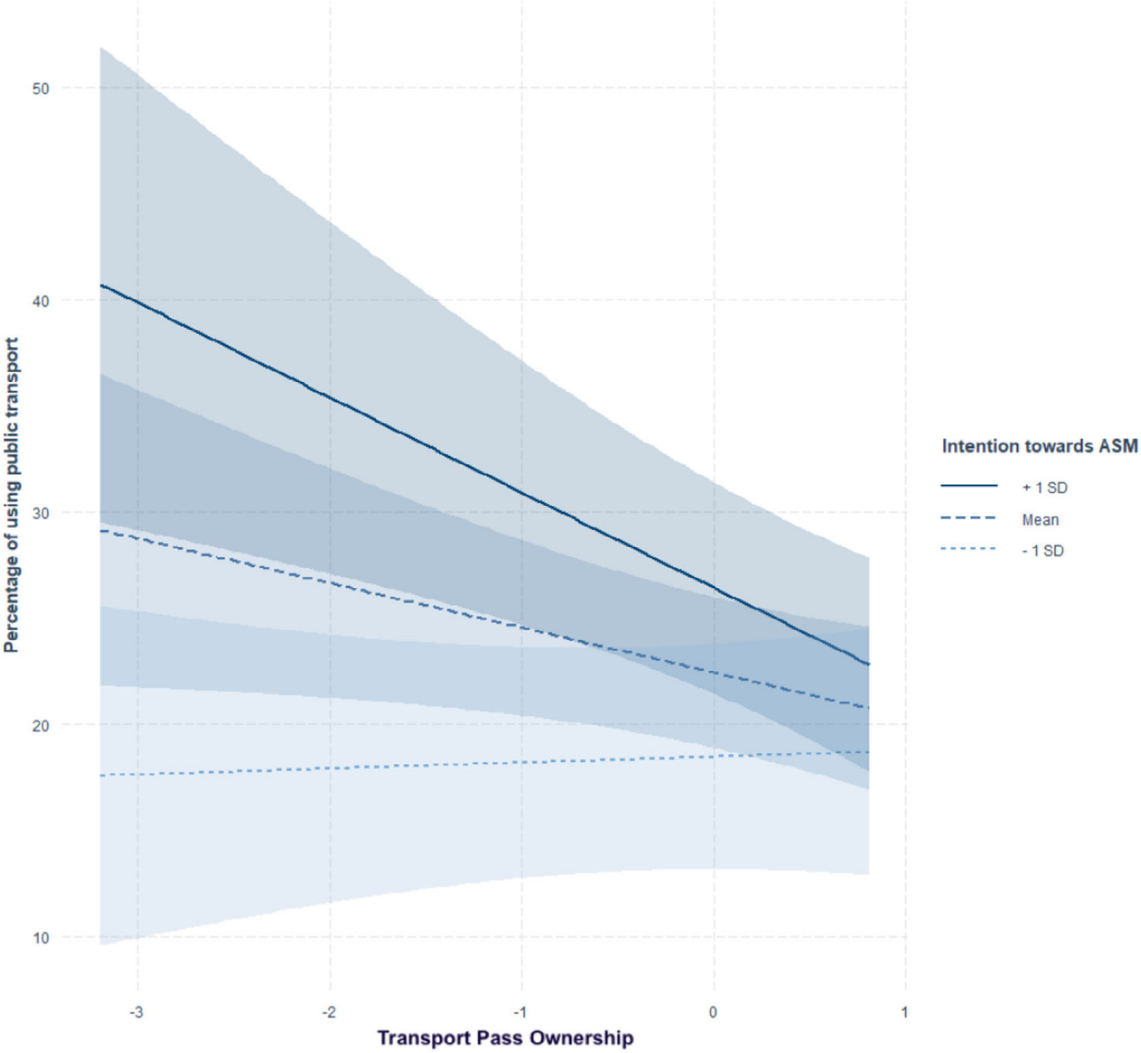
Interaction between transport pass ownership and intention toward ASM on using public transport.

Model 4b was statistically significant (*F* [41, 385] = 11.38, *p* < .001), with an *R* ^2^ of .50 The associations between possession of a transport pass (β = 0.49, *p* < .001), associated ASM habits (β = 0.20, *p* < .001), ASM during first job (β = 0.09, *p* = .031), frequency of complex trip chaining (β = −0.14, *p* = .003), possession of a bike (β = −0.09, *p* = .043), attitude toward ASM (β = −0.14, *p* = .015), and intention toward ASM (β = 0.12, *p* = .030) with public transport use were significant. In addition, one interaction reached statistical significance: possession of a bike × intention toward ASM (β = −0.12, *p* = .003), see Figure 3. Simple slope analyses (see Supplementary Table S12) showed that individuals possessing a bike traveled less often by public transport but only if they presented average (b = −2.10, *p* = .040) or stronger intentions (b = −4.47, *p* < .001) compared to individuals with low intentions toward ASM (b = 0.28, *p* = .800).

### Specific mobility‐related factors of ASM to the Grenoble Metropolitan Area (H4)

The quotations selected illustrate and confirm the consistency between participants' accounts and the identified major themes.

The participants of the qualitative study mentioned that some local and regional investments encourage “car culture” (i.e., a very positive image of the car as a source of flexibility and freedom). In particular, investments in the construction and widening of motorways are perceived as more important than investments in cycling or walking infrastructures. As such, increases in the car's importance in transport and the place it occupies in the public space is perceived as an obstacle to reduce car use and increase ASM:

Expert 9: “*Their whole social model is built around the car. Everything is advertised for the car. What are the first investments in the country? It's the **480 motorway at 300 million euros, it's the RN75 motorway**, **65 million euros.** The big public investments are for the car.”*
Expert 11: *“You look at the economic plan proposed by the government, it's the same thing, “let's go for the bike boost, we'll put twenty million on the table” and then afterwards it's* ***eight billion*** ***for the car industry** …”* (Experts 9 and 11).

Beyond describing policy priorities, these narratives highlight the car's position both as a cultural icon and as a perceived necessity. Notably, the contrast between the massive budgets dedicated to motorways and the relatively minor investments in cycling illustrates how political actors appear eager to sustain—or even reinforce—this perceived necessity.

In addition, one regular car user explained that despite her current car use (mostly because of living in the surrounding Grenoble mountains), she was used to bike during her childhood when she was living in the city center: *“I am someone who has never loved the car. In my **childhood I was always on my bike**, living in **Grenoble city centre**; I was always on my bike. So I discovered the car when I was thirty years old and I am happy to have found other alternatives, and I accept the constraints easily.”* (Car driver 2).

Furthermore, certain mobility experts mentioned that transport facilities and the land‐use planning are not always adapted to ASM and they could be improved (especially in business and activity zones that are poorly deserved in terms of public transportation): *“A lot of people work in **business zones** that have not been planned at all in terms of transport other than car and individual car transport … Afterwards, in terms of transport service to the territory, they are generally areas where you have **zero‐cycle paths and even zero sidewalks**, you can't even get around on foot within the area.”* (Expert 2).

The proposed narratives reflect that some spaces—such as business zones, but also some residential ones—are organized in ways that privilege car access and marginalize other forms of mobility. As researchers, we interpret these accounts as critiques of the structural inequalities embedded in the design of cities and suburbs. This interpretation underlines the importance of examining planning decisions and their role in shaping mobility behaviors, moving beyond a narrow focus on individual responsibility or personal determinants of mobility choices, and particularly those related to ASM.

All these car users repeatedly indicated (versus only one transport expert) that they feel a sense of traffic insecurity when commuting differently than the car. More precisely, car drivers mentioned the danger of suffering incivility while walking, biking or using public transport: *“Whether on foot, on a bike, on a scooter, sometimes you find yourself in situations, even as a pedestrian, **you don't necessarily feel safe**.”* (Car driver 1). Car users mentioned that this insecurity is not only related to the lack of biking or walking facilities, but also because the authorities do not ensure compliance with traffic regulations, especially in suburbs and less urban areas:

So for the time being, we can have facilities that are scaled up in the different large cities, like in Grenoble, but we will never be able to replace that in relation to incivility … **No matter how much more reassuring the facilities are on the safety aspect, if there is incivility, it will be useless**. (Car driver 5).

One car driver recounts the hazardous trip that he made by bike to come to our study: *“So I have a car coming out the wrong way, in front of me. There is a truck parked on the cycle paths. I overtake him, I put the mirror down so that I can pass, **I get insulted**. And I've got a car over there, there's a passage where the path crosses the road in an elevated passage, so it has priority**. A car didn't slow down and insulted me when I was crossing** …”* (Car driver 4).

The different narratives suggest that ASM is perceived not only as inconvenient but also as risky. Indeed, the idea that travelling by modes other than the car exposes individuals to incivilities or to the lack of appropriate infrastructures may reinforce the cultural dominance of the car. Notably, it was primarily women who reported such experiences, which raises broader questions about gendered safety in public spaces and the unequal distribution of vulnerabilities in urban mobility. Moreover, specific geographical characteristics of Grenoble Metropolitan Area (such as the mountains, peripheral areas that surround the city and the housing display in these areas) were reported as a barrier: “*When you live in the mountains, going shopping every day is not possible. In any case, in the mountains you have nothing …”*. (Car driver 1); *“**For people who live in really remote areas, it's complicated**. Sometimes it's even complicated to find a carpool. Those who work in very remote areas, for those who have shifted hours, especially at night, it is hard to take public transport.”* (Expert 7).

While these accounts highlight material constraints, they also serve to naturalize and justify car dependency. This interpretation raises important questions about the extent to which the type of territory is mobilized as a justification for mobility behaviors. As researchers in geography and psychology, we interpreted these narratives as more than factual descriptions of the territory: they reveal how geographical surroundings become embedded in perceptions of mobility, fostering a sense of resignation in which car use appears inevitable in such contexts.

Finally, certain economic and material measures (e.g., economic incentives, preferential subscriptions) regulated by governments or local authorities (specific to French legislation, such as the obligation of employers to pay at least half of the annual or monthly transport passes) can be a lever for some people: *“I can speak from personal experience. **When I learned that my employer could pay 70% of the bus transport pass, I must admit that I didn't hesitate**.”* (Car driver 2).

Overall, the qualitative analysis revealed five major themes structuring participants' accounts. First, both experts and car users emphasized the persistence of a “car culture” reinforced by large‐scale public investments that historically favored motorways and the car industry over active transport infrastructures. Second, infrastructure and land‐use planning were described as poorly adapted to ASM, particularly in business zones in suburbs and remote‐urban areas. Third, participants repeatedly stressed perceptions of traffic insecurity and incivilities when walking, cycling, or using public transport, which were seen as a major deterrent to changing travel mode choices. Fourth, geographical barriers linked to the mountainous context and dispersed housing in the Grenoble metropolitan area were considered to limit the feasibility of ASM. Finally, some participants mentioned economic and material incentives (e.g., reimbursement of public transport passes by employers) as concrete levers to encourage a shift away from car use.

The totality of the coded themes is available on https://osf.io/9h2ck/?view_only=88ea796fe6574b8c9bed7ade6936543c.

**FIGURE 4:**
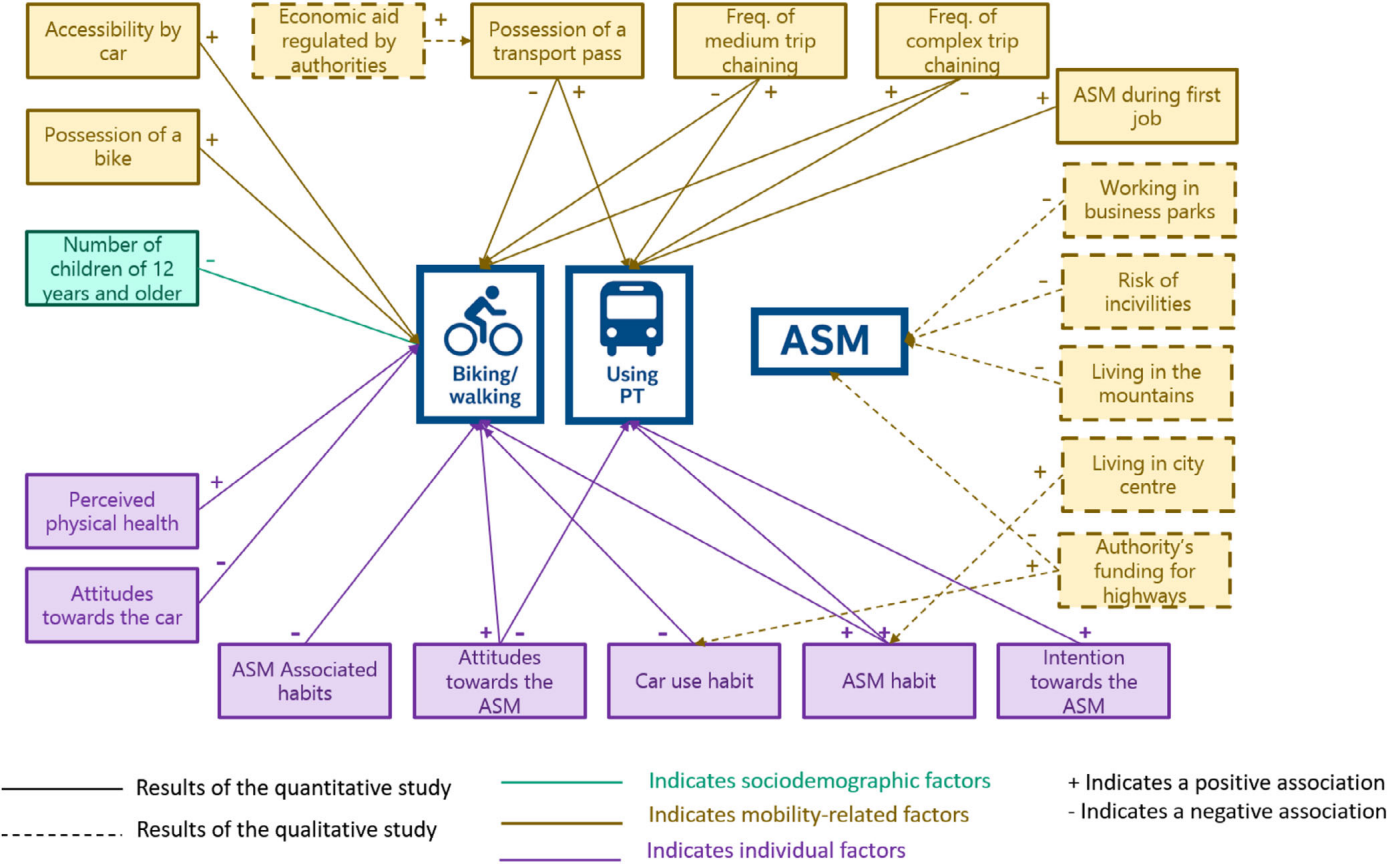
Summary of the results of the quantitative study (independence hypothesis) and the qualitative study. N = Number, ASM = Active and Sustainable mobility, PT = Public transport, Freq = Frequency. Biking/Walking and Using PT were measured by the percentage of trips in a typical week made using these travel modes. Icons were created with the assistance of OPENAI (ChatGPT).

The Figure 4 shows the combination of the results of two studies.

## DISCUSSION

### Main findings

The main contribution of this study is to go beyond person‐centered approaches by enlarging the focus on sociodemographic and socio‐spatial factors of travel mode choices. Overall, the quantitative study supported the independency hypothesis by showing that ASM outcomes were independently associated with mobility‐related (transport pass ownership, complex trip chaining) and individual factors (attitudes toward car use and ASM, and ASM habits). In addition, some factors were outcome‐specific. On the one hand, biking and walking were associated with perceived physical health and car‐use habits at the individual level, with possession of a bike at the mobility level, and with having children aged 12 years or older at the sociodemographic level. On the other hand, public transport and/or carpooling were linked to having practiced ASM during the first job at the mobility level and with intention toward ASM at the individual level.

These results are consistent with past studies showing the role of these variables (De Witte et al., 2013; Kim & Ulfarsson, 2008; Martin et al., 2012, at the socio‐spatial level; Rubin et al., 2014 at the sociodemographic level; Hoffmann et al., 2017; Lanzini & Khan, 2017; McCarthy et al., 2021; Steg, 2005; Vincent‐Geslin, 2010 at the psychological level). The added value of our study to this literature is to examine the role of these variables simultaneously, instead of focusing only on one level.

In contrast, the hypothesis that the effects of socio‐spatial factors are fully driven by more proximal psychological factors (called the *sufficiency hypothesis*, Ajzen, 2011) was mostly not supported, as intention toward ASM mediated only the links between bike/transport pass ownership and public transport or carpooling. The persistence of direct effects of factors at the individual and mobility level indicates that explanations are not exhausted by a single motivational chain, consistent with reviews highlighting independent contributions from multiple levels (Javaid et al., 2020; Huang et al., 2024).

Finally, the effects of some socio‐spatial variables on ASM outcomes were moderated by intention toward ASM. Individuals possessing a bike were more likely to commute by biking or walking when their intentions toward ASM were moderate or high, whereas this association was absent among those with low intentions. Conversely, the negative relationship between public transport pass ownership and biking or walking was stronger among individuals with moderate or high ASM intentions, suggesting a possible substitution between sustainable modes when intentions are high. Similarly, the negative association between bike possession and public transport use was amplified among individuals with moderate and strong ASM intentions. Overall, these findings are consistent with the hypothesis of interaction effects between factors from different levels, in line with a few targeted findings (e.g., price effects varying by gender or depending on infrastructure; Avner et al., 2014; Hensher, 2008), although such effects seem to be infrequently observed in the literature (Rhodes et al., 2018).

Qualitative findings allowed the identification of additional socio‐spatial factors that were specific to the Grenoble Metropolitan Area. Participants reported that urban planning—such as poorly connected business zones—and geographical constraints like mountain or peripheral living could hinder sustainable commuting, reflecting existing literature (Leslie & Cerin, 2008; Panter et al., 2013; Wang et al., 2016). In contrast, living in the city center or the existence of locally/nationally funded motorways were seen to shape habits and attitudes toward travel mode choices. For example, city‐center living may foster ASM routines, while motorways might promote car‐friendly attitudes. Safety concerns also emerged, with participants highlighting a lack of policies addressing risks linked to insecurity and incivility from drivers and other users. The feeling of insecurity during commuting, especially among women, is under‐researched but noted in a few studies (e.g., Sarrafi et al., 2019). Additionally, some economic measures (e.g., mandatory employer contributions to transport passes) were viewed as effective enablers. Qualitative data also allowed to contextualize some results from the quantitative study: w*hile the quantitative analysis identified transport pass possession and early ASM experiences as predictors of public transport use/carpooling, the qualitative interviews enriched these results by suggesting the triggering role of economic incentives and lifelong mobility habits, respectively*.

While the quantitative study outlined the independent nature of the associations between ASM and sociodemographic, socio‐spatial, and individual‐level factors, the qualitative findings offered a complimentary understanding of the levers and barriers that may be specific to the living context of participants. Spatial characteristics such as mountainous terrain, central accessibility, motorway development, and transport gaps in business parks influenced not only mode choices but also individual factors like habits and attitudes. Having a fine‐grained analysis of the specific context is particularly useful for developing tailored interventions such as personalized travel advice, defined as individually tailored information and guidance to encourage a shift from car use toward ASM. Such tailored interventions may be effective to enhance self‐efficacy and knowledge about available transport options, and to reduce in turn car use and increase ASM (Semenescu et al., 2020).

### Strengths and limits

A major strength of this study lies in its original integration of numerous factors across disciplines, supported by a close collaboration between geography and psychology, as recommended by Van Acker et al. (2010) (see also Huang et al., 2024; Javaid et al., 2020). Unlike most interdisciplinary studies, which often include only a limited number of cross‐disciplinary variables (e.g., Bouscasse et al., 2018; Klöckner & Blöbaum, 2010), our approach incorporated a broader range of factors. Additionally, the use of mixed methods strengthened the study's relevance for designing interventions to reduce car use and promote ASM by taking into account the factors of travel mode choices both at the general and local levels (Teran‐Escobar et al., 2022). As noted by Steckler et al. (1992), combining qualitative and quantitative approaches enhances understanding of exploratory questions. By employing a mixed‐methods approach, this research combines breadth and depth (Patton, 1990) to bridge the gap between national‐scale quantitative findings and localized, qualitative insights, needed to inform local public policies and to develop evidence‐based interventions to promote ASM (e.g., Teran‐Escobar et al., 2022).

However, some limitations must be acknowledged. The cross‐sectional design of the quantitative study prevents any causal inference—associations can be identified, but directionality remains unclear (e.g., whether attitudes shape travel mode choices or vice versa). Moreover, the small number of car drivers intending to change their behavior in the qualitative sample limits the generalizability of those findings. Another way of collecting contextual data that could yield additional insights would be to interview car drivers who do not intend to change their behavior. A further limitation is that our study does not fully capture the context‐sensitivity of travel mode choices. As shown by Hoffmann et al. (2018), perceptions of different travel modes vary depending on contextual factors such as trip purpose, distance, or social meaning, and car users and non‐car users construe these modes in systematically different ways. Future research should therefore examine how such contextual factors moderate the influence of psychological, socio‐spatial, and sociodemographic determinants of travel mode choices.

ASM was assessed through self‐reported data, which can lead to inaccuracies, such as overestimating travel time (Kelly et al., 2013). Finally, the qualitative data collection relied on both interviews and focus groups, introducing methodological heterogeneity that may affect comparability.

### Theoretical and practical implications

Theoretical implications of this study highlight the importance of combining interdisciplinary approaches (e.g., geography, psychology, economics) and mixed methods to identify both general and territory‐specific factors influencing daily mobility. This calls for more systemic theoretical frameworks that move beyond disciplinary silos and account for interactions across different levels of the mobility system.

Practically, future experimental and longitudinal studies are needed to explore how psychological factors evolve over time and interact with other influences on mobility behavior (e.g., Teran‐Escobar et al., 2022). Adopting systemic approaches—integrating individual, contextual, and policy‐related factors—could shed light on the impact of socio‐spatial inequalities on mobility change. Identifying these inequalities can help guide public policy and infrastructure planning, including targeted financial support.

Finally, several factors identified in our studies (e.g., car‐related attitudes, perceived insecurity, poor transport access in business areas, self‐efficacy) warrant further investigation with local stakeholders due to their potential role in hindering ASM. Addressing car culture and its symbolic appeal may also require political action, such as regulating advertising—either by reducing its prevalence or including warnings about the environmental and health impacts of car use.

## AUTHOR CONTRIBUTIONS

CT‐E collected, analyzed, and interpreted the data. CT‐E, SD and AC drafted the manuscript and the remaining authors provided suggestions and revisions to the manuscript. All the authors approved the final version of this manuscript for submission.

## CONFLICT OF INTEREST STATEMENT

The authors do not have any potential competing interest to declare.

## ETHICS STATEMENT

This study was reviewed and approved by the ethics committee of Univ. Grenoble‐Alpes (CER Grenoble Alpes‐Avis‐2019‐01‐29‐2).

## Supporting information


**Table S1.** Summary of the variables and measures.
**Table S2.** Matrix of correlations *with confidence intervals.*
**Table S3.** Description of the profiles of the participants in the focus groups and individual interviews.
**Table S4.** Hierarchical regression models testing the independent association between the mobility‐related, sociodemographic, and psychological factors and Biking and Walking (Hypothesis 1).
**Table S5.** Hierarchical regression models testing the independent association between the mobility‐related, sociodemographic, and psychological factors and using Public Transport (Hypothesis 1).
**Table S6.** Hierarchical regression models testing if the association between the mobility‐related and sociodemographic factors and active and sustainable mobility is mediated by psychological factors (Hypothesis 2).
**Table S7.** Indirect effects of mobility‐related variables on using public transport through psychological variables (Hypothesis 2).
**Table S8.** Stepwise regression model testing if the association between the mobility‐related and sociodemographic factors and active and biking/walking is moderated by psychological factors (Hypothesis 3).
**Table S9.** Stepwise regression model testing if the association between the mobility‐related and sociodemographic factors and active and using public transport is moderated by psychological factors (Hypothesis 3).
Table S10. Slope of the interaction between possessing a bike x intention toward ASM on biking/walking.
**Table S11.** Slopes of the interaction between possessing a public transport pass x intention toward ASM on biking/walking.
**Table S12.** Slopes of the interaction between possessing a bike x intention toward ASM using public transport.
**Table S12.** Slopes of the interaction between possessing a bike x intention toward ASM using public transport.
**Figure S1.** Tested framework for Hypothesis 1 (Independent associations) and hypothesis 4 (Specific levers and obstacles). Note. N = Number, ASM = Active and Sustainable mobility, PT = Public transport, Freq = Frequency. Icons were created with the assistance of OPENAI (ChatGPT).
**Figure S2.** Tested framework for Hypothesis 2 (Mediated associations) and Hypothesis 3 (Moderated associations). Icons were created with the assistance of OPENAI (ChatGPT). Note. ASM = Active and Sustainable mobility.
**Figure S3.** Plot of model 1b (Model predicting biking/walking) assumptions (linearity, homogeneity of variance, collinearity, normality of residuals, and normality of random effects). The blue dots represent the observations.
**Figure S4.** Plot of model 2b (Model predicting using public transport) assumptions (linearity, homogeneity of variance, collinearity, normality of residuals, and normality of random effects). The blue dots represent the observations.
**Figure S5.** Plot of model 4a (predicting biking/walking) assumptions (linearity, homogeneity of variance, collinearity, normality of residuals, and normality of random effects). The blue dots represent the observations. Plot of model 4b (predicting using public transport) assumptions (linearity, homogeneity of variance, collinearity, normality of residuals, and normality of random effects). The blue dots represent the observations.
Data S1: Presentation of the quantitative study and informed consent.
Data S2: Informed consent of the qualitative study.
Data S3: Physical activity and full scales of psychological constructs.
Data S4: Protocol of the qualitative study (experts).

## Data Availability

The R code and the dataset for this research are available in the open platform OSFHOME (https://osf.io/9h2ck/?view_only=88ea796fe6574b8c9bed7ade6936543c).
