# Supplementary material for: Beyond person‐centered approaches: Integrating individual, sociodemographic and socio‐spatial factors to better understand active and sustainable mobility
Source: Appl Psychol Health Well Being. 2026 Mar 6;18(2):e70136. doi: 10.1111/aphw.70136 (PMC12965119; doi:10.1111/aphw.70136)
Supplement: Supplementary file 1 — Table S1. Summary of the variables and measures. Table S2. Matrix of correlations with confidence intervals. Table S3. Description of the profiles of the participants in the focus groups and individual interviews. Table S4. Hierarchical regression models testing the independent association between the mobility‐related, sociodemographic, and psychological factors and Biking and Walking (Hypothesis 1). Table S5. Hierarchical regression models testing the independent association between the mobility‐related, sociodemographic, and psychological factors and using Public Transport (Hypothesis 1). Table S6. Hierarchical regression models testing if the association between the mobility‐related and sociodemographic factors and active and sustainable mobility is mediated by psychological factors (Hypothesis 2). Table S7. Indirect effects of mobility‐related variables on using public transport through psychological variables (Hypothesis 2). Table S8. Stepwise regression model testing if the association between the mobility‐related and sociodemographic factors and active and biking/walking is moderated by psychological factors (Hypothesis 3). Table S9. Stepwise regression model testing if the association between the mobility‐related and sociodemographic factors and active and using public transport is moderated by psychological factors (Hypothesis 3). Table S10. Slope of the interaction between possessing a bike x intention toward ASM on biking/walking. Table S11. Slopes of the interaction between possessing a public transport pass x intention toward ASM on biking/walking. Table S12. Slopes of the interaction between possessing a bike x intention toward ASM using public transport. Table S12. Slopes of the interaction between possessing a bike x intention toward ASM using public transport. Figure S1. Tested framework for Hypothesis 1 (Independent associations) and hypothesis 4 (Specific levers and obstacles). Note. N = Number, ASM = Active and Sustainable mobility, PT = Public t [file APHW-18-0-s001.docx]

Supplemental Material Files

Supplementary Table S1

Summary of the variables and measures.

| **Variable** | **Details** | |
| --- | --- | --- |
| **Dependent variable** | | |
| Percentage of walking or biking during a typical week | Percentage of trips done by classic or electric bike and walk. Scale from 0 to 100% | |
| Percentage of public transport use or carpooling during a typical week | Percentage of trips done by public transport, carpooling. Scale from 0 to 100% | |
| **Sociodemographic variables** | | |
| Gender, age, educational attainment, income, work percentage, number of persons in the household, number of children under 12 years and 12 years and older, habitat surface |  | |
| **Mobility-related variables** | | |
| Number of cars in the household |  |  |
| Possession of a bike | 1 (not possessing a bike) to 4 (possessing a functional bike) | |
| Possession of a transport pass | 0 (not possessing a transport pass) to 4 (possessing an annual transport pass) | |
| Population density around the domicile | People per square kilometre ratio was calculated by using the domicile address to recuperate the data from the IRIS database | |
| Accessibility by car | 1 (not parking at all) to 3 (having parking nearby) | |
| Proximity to a public transport stop | 1 (not stop at all) to 3 (having a PT stop nearby) | |
| Principal motive of commuting | Work or study, purchases and other services, accompany someone and “other” such as leisure, sports or cultural activities, volunteering | |
| Frequency of simple trip chaining (2 activities) | 1 (never) to 4 (almost every day) | |
| Frequency of medium trip chaining (3 or 4 activities) |  |  |
| Frequency of complex trip chaining (chaining 5 or more activities in the same travel) |  |  |
| ASM during elementary school, high school, university and first job | 1 (mostly in the car) to 3 (mostly in active mobility)  Müggenburg et al. (2015) | |
| **Individual Variables** | | |
| Intention toward ASM | 1 (no intention at all) to 7 (very strong intention)  Godin (2012) | |
| Self-efficacy toward ASM | 1 (not confident at all) to 7 (very confident)  Schwarzer et al. (2015) | |
| Attitude toward car | Average 2 items. 1 (not agree at all) to 7 (completely agree)  Average of perceived social pressure 1 (not agree at all) to 7 (completely agree) and perceived percentage of relatives using ASM 1 (no one) to 7 (everybody)  Godin (2012) | |
| Attitude toward ASM |  |  |
| Subjective norms of ASM |  |  |
| Car habits | Average 4 items. 1 (not agree at all) to 7 (completely agree)  Good reliability (α= .93, α= .96)  Gardner et al. (2012) | |
| ASM habits |  |  |
| Associated ASM habits | Average 7 items (e.g., reading, listening to the radio during commuting). 1 (never) to 5 (Always)  Acceptable reliability (α= .60)  Buhler (2012) | |
| Green identity | Average 5 items. 1 (not agree at all) to 7 (completely agree)  Good reliability (α= .80).  Lalot et al. (2019) | |
| Perceived risks of getting COVID-19 | Average 5 items. 1 (not agree at all) to 7 (completely agree)  Acceptable reliability (α= .73)  Nexøe et al. (1999) | |
| Moderate-to-vigorous PA | Weekly moderate and vigorous physical activity minutes during the last week  IPAQ (Craig et al., 2003) | |
| Perceived physical health | Average 5 items. 1 (not agree at all) to 7 (completely agree)  Sub-scale SF-12 (Ware et al., 1996) | |

Supplementary Table S2

Matrix of correlations *with confidence intervals*

| Variable | *M* | *SD* | 1 | 2 | 3 | 4 | 5 | 6 | 7 | 8 | 9 | 10 | 11 | 12 | 13 | 14 | 15 |
| --- | --- | --- | --- | --- | --- | --- | --- | --- | --- | --- | --- | --- | --- | --- | --- | --- | --- |
| 1. bik | 49.00 | 42.77 |  |  |  |  |  |  |  |  |  |  |  |  |  |  |  |
| 2. PT | 22.14 | 34.69 | -.32^**^ |  |  |  |  |  |  |  |  |  |  |  |  |  |  |
|  |  |  | [-.39, -.24] |  |  |  |  |  |  |  |  |  |  |  |  |  |  |
| 3. Age | 38.00 | 11.24 | -.04 | -.17^**^ |  |  |  |  |  |  |  |  |  |  |  |  |  |
|  |  |  | [-.12, .05] | [-.25, -.09] |  |  |  |  |  |  |  |  |  |  |  |  |  |
| 4. Sex | 1.45 | 0.50 | .09^*^ | -.02 | .09^*^ |  |  |  |  |  |  |  |  |  |  |  |  |
|  |  |  | [.01, .18] | [-.11, .06] | [.00, .17] |  |  |  |  |  |  |  |  |  |  |  |  |
| 5. Ed | 5.98 | 1.03 | .16^**^ | .08 | -.07 | .07 |  |  |  |  |  |  |  |  |  |  |  |
|  |  |  | [.07, .24] | [-.01, .16] | [-.15, .02] | [-.02, .15] |  |  |  |  |  |  |  |  |  |  |  |
| 6. Inc | 4.40 | 1.41 | -.03 | -.07 | .44^**^ | .07 | .26^**^ |  |  |  |  |  |  |  |  |  |  |
|  |  |  | [-.11, .06] | [-.15, .02] | [.37, .51] | [-.01, .15] | [.17, .33] |  |  |  |  |  |  |  |  |  |  |
| 7. %Wo | 93.18 | 16.97 | .05 | -.07 | .04 | .02 | .17^**^ | .21^**^ |  |  |  |  |  |  |  |  |  |
|  |  |  | [-.03, .14] | [-.15, .02] | [-.04, .13] | [-.06, .11] | [.09, .25] | [.13, .29] |  |  |  |  |  |  |  |  |  |
| 8. Hou | 2.50 | 1.32 | -.03 | -.06 | .33^**^ | .10^*^ | .05 | .48^**^ | -.04 |  |  |  |  |  |  |  |  |
|  |  |  | [-.11, .06] | [-.14, .03] | [.25, .40] | [.01, .18] | [-.04, .13] | [.41, .54] | [-.12, .05] |  |  |  |  |  |  |  |  |
| 9. Ch-12 | 0.44 | 0.84 | .01 | -.04 | .07 | .07 | .08 | .23^**^ | -.05 | .68^**^ |  |  |  |  |  |  |  |
|  |  |  | [-.08, .09] | [-.12, .04] | [-.02, .15] | [-.02, .15] | [-.01, .16] | [.14, .31] | [-.13, .04] | [.63, .72] |  |  |  |  |  |  |  |
| 10. Ch+12 | 0.35 | 0.84 | -.06 | .01 | .38^**^ | .03 | .03 | .27^**^ | .04 | .48^**^ | -.07 |  |  |  |  |  |  |
|  |  |  | [-.14, .02] | [-.07, .10] | [.30, .45] | [-.06, .11] | [-.06, .11] | [.19, .35] | [-.04, .13] | [.41, .54] | [-.15, .02] |  |  |  |  |  |  |
| 11. Sur | 86.00 | 47.17 | -.09^*^ | -.15^**^ | .58^**^ | .08 | .03 | .52^**^ | .04 | .61^**^ | .30^**^ | .42^**^ |  |  |  |  |  |
|  |  |  | [-.17, -.00] | [-.23, -.07] | [.52, .64] | [-.00, .17] | [-.05, .12] | [.45, .58] | [-.05, .12] | [.55, .66] | [.22, .37] | [.35, .49] |  |  |  |  |  |
| 12. BikP | 3.19 | 1.56 | .38^**^ | -.15^**^ | .13^**^ | .08 | .07 | .12^**^ | -.03 | .19^**^ | .14^**^ | .07 | .17^**^ |  |  |  |  |
|  |  |  | [.31, .45] | [-.23, -.07] | [.05, .21] | [-.01, .16] | [-.02, .15] | [.04, .20] | [-.12, .05] | [.11, .27] | [.06, .23] | [-.01, .16] | [.09, .25] |  |  |  |  |
| 13. PtP | 1.78 | 1.24 | -.14^**^ | .65^**^ | -.19^**^ | -.07 | .07 | -.07 | -.02 | -.10^*^ | -.12^**^ | .03 | -.15^**^ | -.16^**^ |  |  |  |
|  |  |  | [-.22, -.06] | [.60, .70] | [-.27, -.11] | [-.15, .02] | [-.01, .16] | [-.15, .02] | [-.10, .07] | [-.18, -.01] | [-.20, -.03] | [-.05, .12] | [-.24, -.07] | [-.24, -.07] |  |  |  |
| 14. Ncar | 1.21 | 0.97 | -.32^**^ | -.24^**^ | .32^**^ | .01 | -.15^**^ | .35^**^ | .07 | .37^**^ | .12^**^ | .21^**^ | .45^**^ | .03 | -.28^**^ |  |  |
|  |  |  | [-.39, -.24] | [-.32, -.16] | [.25, .40] | [-.08, .09] | [-.23, -.06] | [.27, .42] | [-.02, .15] | [.29, .44] | [.04, .21] | [.13, .29] | [.38, .52] | [-.05, .12] | [-.35, -.20] |  |  |
| 15. Den | 8707.13 | 11485.11 | .18^**^ | .23^**^ | -.23^**^ | -.00 | .10^*^ | -.10^*^ | -.00 | -.12^**^ | -.09^*^ | -.13^**^ | -.30^**^ | -.08 | .20^**^ | -.38^**^ |  |
|  |  |  | [.10, .26] | [.15, .31] | [-.31, -.15] | [-.09, .08] | [.01, .18] | [-.18, -.02] | [-.09, .08] | [-.21, -.04] | [-.17, -.00] | [-.21, -.04] | [-.38, -.22] | [-.17, .00] | [.12, .28] | [-.45, -.30] |  |
| 16. CarA | 2.57 | 0.81 | -.04 | -.15^**^ | .03 | .05 | .04 | .17^**^ | .11^*^ | .06 | .07 | .01 | .09^*^ | .04 | -.12^**^ | .21^**^ | -.27^**^ |
|  |  |  | [-.12, .05] | [-.23, -.06] | [-.06, .12] | [-.03, .14] | [-.05, .13] | [.08, .25] | [.02, .20] | [-.03, .14] | [-.02, .15] | [-.08, .10] | [.00, .18] | [-.05, .12] | [-.20, -.03] | [.12, .29] | [-.34, -.18] |
| 17. PtA | 2.71 | 0.63 | .28^**^ | .06 | -.19^**^ | .06 | .10^*^ | -.06 | .06 | -.10^*^ | -.04 | -.06 | -.20^**^ | .05 | .05 | -.33^**^ | .27^**^ |
|  |  |  | [.20, .36] | [-.02, .14] | [-.27, -.11] | [-.03, .14] | [.02, .18] | [-.15, .02] | [-.03, .14] | [-.18, -.02] | [-.13, .04] | [-.14, .03] | [-.28, -.12] | [-.03, .14] | [-.03, .14] | [-.41, -.25] | [.19, .35] |
| 18. Mot | 1.42 | 1.41 | -.01 | -.03 | .05 | .01 | .06 | -.00 | -.07 | .14^**^ | .08 | .16^**^ | .09^*^ | .02 | -.02 | .02 | -.06 |
|  |  |  | [-.10, .07] | [-.11, .06] | [-.03, .13] | [-.08, .09] | [-.03, .14] | [-.09, .08] | [-.15, .01] | [.05, .22] | [-.00, .17] | [.08, .24] | [.01, .18] | [-.06, .11] | [-.10, .07] | [-.07, .10] | [-.14, .03] |
| 19. ChA | 2.75 | 0.83 | .05 | -.02 | -.10^*^ | -.09^*^ | .02 | -.02 | .05 | -.01 | .10^*^ | -.11^*^ | -.07 | .08 | -.03 | -.02 | .10^*^ |
|  |  |  | [-.03, .13] | [-.11, .06] | [-.19, -.02] | [-.17, -.01] | [-.07, .10] | [-.11, .06] | [-.04, .13] | [-.10, .07] | [.02, .19] | [-.19, -.02] | [-.15, .02] | [-.01, .16] | [-.11, .06] | [-.10, .07] | [.02, .19] |
|  |  |  |  |  |  |  |  |  |  |  |  |  |  |  |  |  |  |
| 20. ChB | 1.93 | 0.81 | .02 | .01 | -.03 | -.13^**^ | .04 | -.01 | -.06 | .08 | .12^**^ | -.03 | .02 | .07 | .01 | .04 | .02 |
|  |  |  | [-.07, .11] | [-.08, .09] | [-.12, .05] | [-.21, -.04] | [-.05, .12] | [-.10, .08] | [-.15, .02] | [-.01, .17] | [.03, .20] | [-.11, .06] | [-.07, .10] | [-.02, .15] | [-.07, .10] | [-.04, .13] | [-.06, .11] |
| 21. ChC | 1.36 | 0.61 | .06 | -.05 | -.04 | -.09^*^ | -.08 | -.05 | -.07 | .03 | .02 | -.03 | .02 | .07 | .02 | .02 | -.00 |
|  |  |  | [-.03, .15] | [-.14, .04] | [-.13, .05] | [-.18, -.00] | [-.16, .01] | [-.14, .04] | [-.16, .02] | [-.05, .12] | [-.06, .11] | [-.12, .06] | [-.07, .10] | [-.02, .16] | [-.07, .10] | [-.06, .11] | [-.09, .09] |
| 22. Bio1 | 2.31 | 0.91 | -.00 | -.03 | .20^**^ | -.02 | -.00 | .06 | .03 | .05 | .03 | .02 | .10^*^ | .00 | -.04 | .08 | -.04 |
|  |  |  | [-.09, .08] | [-.12, .05] | [.12, .28] | [-.10, .07] | [-.09, .08] | [-.03, .14] | [-.06, .11] | [-.04, .13] | [-.06, .11] | [-.07, .10] | [.01, .18] | [-.08, .09] | [-.13, .04] | [-.00, .17] | [-.13, .04] |
| 23. Bio2 | 2.64 | 0.63 | .04 | -.00 | .14^**^ | .09^*^ | -.01 | .06 | .02 | .07 | -.00 | .05 | .09^*^ | .04 | -.03 | .08 | -.02 |
|  |  |  | [-.05, .13] | [-.09, .09] | [.05, .22] | [.01, .18] | [-.10, .08] | [-.02, .15] | [-.07, .10] | [-.02, .15] | [-.09, .08] | [-.03, .14] | [.00, .17] | [-.05, .12] | [-.11, .06] | [-.01, .16] | [-.10, .07] |
| 24. Bio3 | 2.65 | 0.69 | .17^**^ | .09^*^ | -.17^**^ | -.09^*^ | .14^**^ | -.04 | .01 | -.05 | -.01 | -.08 | -.09^*^ | .03 | .10^*^ | -.23^**^ | .12^**^ |
|  |  |  | [.09, .25] | [.01, .18] | [-.25, -.08] | [-.18, -.01] | [.06, .23] | [-.12, .05] | [-.07, .10] | [-.13, .04] | [-.09, .08] | [-.17, .00] | [-.18, -.01] | [-.06, .11] | [.02, .19] | [-.31, -.15] | [.03, .20] |
| 25. Bio4 | 2.41 | 0.87 | .26^**^ | .24^**^ | -.17^**^ | -.02 | .18^**^ | -.10^*^ | .04 | -.05 | -.00 | -.04 | -.19^**^ | .09^*^ | .18^**^ | -.40^**^ | .24^**^ |
|  |  |  | [.18, .34] | [.15, .31] | [-.25, -.09] | [-.11, .06] | [.10, .26] | [-.18, -.01] | [-.05, .12] | [-.14, .03] | [-.09, .08] | [-.12, .05] | [-.27, -.11] | [.01, .17] | [.09, .26] | [-.47, -.33] | [.15, .31] |
| 26. MVPA | 513.08 | 488.46 | .08 | -.04 | -.11^*^ | -.02 | -.01 | -.03 | .00 | -.10^*^ | -.08 | -.06 | -.07 | .04 | -.01 | -.05 | .08 |
|  |  |  | [-.00, .16] | [-.13, .04] | [-.19, -.02] | [-.11, .06] | [-.09, .07] | [-.12, .05] | [-.08, .09] | [-.19, -.02] | [-.17, .00] | [-.15, .02] | [-.15, .02] | [-.05, .12] | [-.09, .08] | [-.13, .04] | [-.00, .17] |
| 27. PhyH | 3.29 | 0.34 | .04 | -.02 | .03 | .13^**^ | .02 | .10^*^ | -.02 | .00 | .02 | .00 | .04 | .16^**^ | -.01 | .09^*^ | .00 |
|  |  |  | [-.04, .13] | [-.10, .07] | [-.05, .12] | [.05, .22] | [-.07, .10] | [.02, .19] | [-.10, .07] | [-.08, .09] | [-.07, .10] | [-.08, .09] | [-.05, .12] | [.07, .24] | [-.09, .08] | [.01, .17] | [-.08, .09] |
| 28. Risk | 2.91 | 1.20 | -.07 | -.07 | .01 | .02 | -.03 | .04 | -.04 | -.01 | -.01 | -.00 | -.01 | -.10^*^ | -.06 | -.02 | .02 |
|  |  |  | [-.15, .02] | [-.16, .01] | [-.07, .10] | [-.07, .10] | [-.12, .05] | [-.05, .12] | [-.12, .05] | [-.09, .08] | [-.09, .08] | [-.08, .08] | [-.09, .08] | [-.18, -.01] | [-.15, .02] | [-.11, .06] | [-.06, .11] |
| 29. InASM | 4.89 | 2.42 | .47^**^ | .22^**^ | -.15^**^ | .00 | .23^**^ | -.05 | .04 | -.06 | -.04 | -.01 | -.18^**^ | .26^**^ | .20^**^ | -.35^**^ | .23^**^ |
|  |  |  | [.40, .53] | [.14, .30] | [-.23, -.07] | [-.08, .09] | [.14, .30] | [-.14, .03] | [-.04, .13] | [-.14, .03] | [-.12, .05] | [-.09, .08] | [-.26, -.09] | [.18, .33] | [.12, .28] | [-.42, -.27] | [.14, .31] |
| 30. EfASM | 4.92 | 2.41 | .56^**^ | .18^**^ | -.12^**^ | .08 | .26^**^ | -.04 | .07 | -.07 | -.00 | -.01 | -.13^**^ | .25^**^ | .18^**^ | -.41^**^ | .26^**^ |
|  |  |  | [.50, .61] | [.10, .26] | [-.20, -.03] | [-.00, .16] | [.18, .34] | [-.12, .05] | [-.01, .16] | [-.15, .02] | [-.08, .08] | [-.10, .07] | [-.21, -.05] | [.17, .33] | [.10, .26] | [-.48, -.34] | [.18, .34] |
| 31. AtCar | 3.12 | 1.96 | -.62^**^ | -.22^**^ | .15^**^ | -.07 | -.23^**^ | .06 | -.05 | .08 | .02 | .04 | .19^**^ | -.28^**^ | -.20^**^ | .53^**^ | -.32^**^ |
|  |  |  | [-.67, -.57] | [-.30, -.14] | [.07, .23] | [-.15, .02] | [-.31, -.15] | [-.03, .14] | [-.14, .03] | [-.00, .17] | [-.07, .10] | [-.04, .12] | [.10, .27] | [-.35, -.20] | [-.28, -.12] | [.47, .59] | [-.39, -.24] |
| 32. AtASM | 4.98 | 1.88 | .58^**^ | .09^*^ | -.12^**^ | .01 | .24^**^ | -.06 | .02 | -.04 | -.01 | -.01 | -.13^**^ | .30^**^ | .13^**^ | -.37^**^ | .20^**^ |
|  |  |  | [.53, .64] | [.00, .17] | [-.20, -.04] | [-.07, .10] | [.16, .32] | [-.14, .03] | [-.06, .11] | [-.13, .04] | [-.10, .07] | [-.10, .07] | [-.21, -.04] | [.23, .38] | [.05, .21] | [-.44, -.30] | [.12, .28] |
| 33. NmASM | 3.72 | 1.29 | .23^**^ | .11^**^ | -.10^*^ | .04 | .15^**^ | .01 | .08 | .02 | .04 | -.01 | -.04 | .13^**^ | .07 | -.21^**^ | .19^**^ |
|  |  |  | [.15, .31] | [.03, .20] | [-.18, -.01] | [-.04, .13] | [.06, .23] | [-.07, .10] | [-.00, .17] | [-.06, .11] | [-.05, .12] | [-.09, .08] | [-.12, .05] | [.05, .21] | [-.02, .15] | [-.29, -.13] | [.10, .27] |
| 34. HCar | 2.30 | 1.77 | -.54^**^ | -.19^**^ | .11^*^ | -.03 | -.26^**^ | .06 | -.08 | .07 | .04 | .00 | .19^**^ | -.20^**^ | -.21^**^ | .49^**^ | -.30^**^ |
|  |  |  | [-.59, -.47] | [-.27, -.11] | [.02, .19] | [-.11, .06] | [-.34, -.18] | [-.03, .14] | [-.16, .00] | [-.02, .15] | [-.04, .12] | [-.08, .09] | [.10, .27] | [-.28, -.12] | [-.29, -.13] | [.42, .55] | [-.37, -.22] |
| 35. HASM | 4.51 | 2.22 | .48^**^ | .23^**^ | -.18^**^ | .05 | .17^**^ | -.06 | -.02 | -.06 | .00 | -.05 | -.21^**^ | .21^**^ | .22^**^ | -.47^**^ | .31^**^ |
|  |  |  | [.42, .55] | [.15, .31] | [-.26, -.10] | [-.03, .14] | [.09, .25] | [-.14, .03] | [-.10, .06] | [-.14, .02] | [-.08, .09] | [-.14, .03] | [-.29, -.13] | [.13, .29] | [.14, .30] | [-.53, -.40] | [.23, .38] |
| 36. HAASM | 2.45 | 0.90 | -.17^**^ | .41^**^ | -.26^**^ | -.05 | .11^*^ | -.06 | -.04 | -.11^**^ | -.04 | -.08 | -.18^**^ | -.15^**^ | .34^**^ | -.16^**^ | .10^*^ |
|  |  |  | [-.25, -.09] | [.33, .47] | [-.34, -.18] | [-.13, .04] | [.02, .19] | [-.14, .03] | [-.13, .04] | [-.20, -.03] | [-.12, .04] | [-.17, .00] | [-.26, -.10] | [-.23, -.07] | [.27, .42] | [-.24, -.08] | [.01, .18] |
| 37. IdEc | 5.96 | 0.98 | .24^**^ | .05 | .05 | .03 | .19^**^ | .15^**^ | .03 | .11^**^ | .04 | .07 | .06 | .27^**^ | -.01 | -.12^**^ | .08 |
|  |  |  | [.16, .32] | [-.04, .13] | [-.04, .13] | [-.05, .12] | [.10, .27] | [.07, .23] | [-.06, .11] | [.03, .20] | [-.04, .13] | [-.02, .15] | [-.03, .14] | [.19, .35] | [-.10, .07] | [-.20, -.04] | [-.00, .17] |

| Variable | 16 | 17 | 18 | 19 | 20 | 21 | 22 | 23 | 24 | 25 | 26 | 27 | 28 | 29 | 30 | 31 | 32 | 33 | 34 | 35 | 36 |
| --- | --- | --- | --- | --- | --- | --- | --- | --- | --- | --- | --- | --- | --- | --- | --- | --- | --- | --- | --- | --- | --- |
|  |  |  |  |  |  |  |  |  |  |  |  |  |  |  |  |  |  |  |  |  |  |
| 17. PtA | -.03 |  |  |  |  |  |  |  |  |  |  |  |  |  |  |  |  |  |  |  |  |
|  | [-.12, .06] |  |  |  |  |  |  |  |  |  |  |  |  |  |  |  |  |  |  |  |  |
| 18. Mot | -.02 | -.01 |  |  |  |  |  |  |  |  |  |  |  |  |  |  |  |  |  |  |  |
|  | [-.11, .07] | [-.10, .07] |  |  |  |  |  |  |  |  |  |  |  |  |  |  |  |  |  |  |  |
| 19. ChA | .02 | .02 | .08 |  |  |  |  |  |  |  |  |  |  |  |  |  |  |  |  |  |  |
|  | [-.07, .11] | [-.06, .11] | [-.01, .16] |  |  |  |  |  |  |  |  |  |  |  |  |  |  |  |  |  |  |
|  |  |  |  |  |  |  |  |  |  |  |  |  |  |  |  |  |  |  |  |  |  |
| 20. ChB | -.04 | .02 | .08 | .65^**^ |  |  |  |  |  |  |  |  |  |  |  |  |  |  |  |  |  |
|  | [-.13, .04] | [-.07, .10] | [-.01, .16] | [.60, .70] |  |  |  |  |  |  |  |  |  |  |  |  |  |  |  |  |  |
| 21. ChC | -.07 | .01 | .11^*^ | .42^**^ | .67^**^ |  |  |  |  |  |  |  |  |  |  |  |  |  |  |  |  |
|  | [-.16, .02] | [-.07, .10] | [.02, .19] | [.34, .49] | [.62, .71] |  |  |  |  |  |  |  |  |  |  |  |  |  |  |  |  |
| 22. Bio1 | .01 | .02 | -.00 | -.10^*^ | -.02 | .00 |  |  |  |  |  |  |  |  |  |  |  |  |  |  |  |
|  | [-.08, .09] | [-.07, .11] | [-.09, .08] | [-.19, -.02] | [-.10, .07] | [-.09, .09] |  |  |  |  |  |  |  |  |  |  |  |  |  |  |  |
| 23. Bio2 | .01 | .06 | .02 | -.06 | -.04 | -.00 | .36^**^ |  |  |  |  |  |  |  |  |  |  |  |  |  |  |
|  | [-.08, .10] | [-.02, .15] | [-.07, .10] | [-.15, .02] | [-.13, .04] | [-.09, .08] | [.29, .43] |  |  |  |  |  |  |  |  |  |  |  |  |  |  |
| 24. Bio3 | -.05 | .10^*^ | .02 | .02 | -.01 | -.06 | .02 | .01 |  |  |  |  |  |  |  |  |  |  |  |  |  |
|  | [-.14, .04] | [.01, .18] | [-.07, .10] | [-.07, .11] | [-.09, .08] | [-.15, .03] | [-.06, .11] | [-.07, .10] |  |  |  |  |  |  |  |  |  |  |  |  |  |
| 25. Bio4 | -.14^**^ | .21^**^ | .10^*^ | .00 | -.06 | -.05 | .01 | .05 | .39^**^ |  |  |  |  |  |  |  |  |  |  |  |  |
|  | [-.23, -.06] | [.13, .29] | [.02, .18] | [-.08, .09] | [-.14, .03] | [-.14, .04] | [-.07, .10] | [-.04, .14] | [.32, .46] |  |  |  |  |  |  |  |  |  |  |  |  |
| 26. MVPA | -.01 | -.01 | -.04 | .13^**^ | .09^*^ | .08 | -.05 | .03 | .03 | .01 |  |  |  |  |  |  |  |  |  |  |  |
|  | [-.10, .07] | [-.09, .08] | [-.12, .05] | [.05, .22] | [.01, .18] | [-.01, .16] | [-.13, .04] | [-.06, .11] | [-.06, .11] | [-.08, .09] |  |  |  |  |  |  |  |  |  |  |  |
| 27. PhyH | .08 | -.04 | -.01 | .07 | .06 | .03 | .04 | .01 | -.12^**^ | -.16^**^ | .11^*^ |  |  |  |  |  |  |  |  |  |  |
|  | [-.01, .17] | [-.13, .04] | [-.10, .07] | [-.02, .15] | [-.02, .15] | [-.06, .12] | [-.05, .12] | [-.07, .10] | [-.20, -.04] | [-.24, -.08] | [.02, .19] |  |  |  |  |  |  |  |  |  |  |
| 28. Risk | -.06 | -.05 | -.00 | -.07 | -.09^*^ | -.12^**^ | -.01 | -.09^*^ | .03 | -.02 | -.07 | -.14^**^ |  |  |  |  |  |  |  |  |  |
|  | [-.15, .03] | [-.13, .04] | [-.09, .08] | [-.16, .01] | [-.17, -.00] | [-.21, -.04] | [-.10, .07] | [-.17, -.00] | [-.06, .11] | [-.10, .07] | [-.15, .01] | [-.22, -.06] |  |  |  |  |  |  |  |  |  |
| 29. InASM | -.01 | .22^**^ | -.02 | .04 | .04 | .01 | .02 | .02 | .20^**^ | .33^**^ | .03 | -.03 | -.08 |  |  |  |  |  |  |  |  |
|  | [-.10, .08] | [.14, .30] | [-.11, .06] | [-.04, .13] | [-.05, .12] | [-.08, .10] | [-.06, .11] | [-.06, .11] | [.11, .28] | [.25, .40] | [-.06, .11] | [-.11, .05] | [-.16, .01] |  |  |  |  |  |  |  |  |
| 30. EfASM | -.10^*^ | .30^**^ | -.01 | .04 | .03 | .01 | .01 | .05 | .17^**^ | .37^**^ | .02 | .01 | -.14^**^ | .68^**^ |  |  |  |  |  |  |  |
|  | [-.19, -.01] | [.22, .38] | [-.09, .08] | [-.05, .12] | [-.06, .11] | [-.08, .10] | [-.07, .10] | [-.03, .14] | [.09, .26] | [.30, .44] | [-.07, .10] | [-.08, .09] | [-.22, -.06] | [.63, .72] |  |  |  |  |  |  |  |
| 31. AtCar | .16^**^ | -.36^**^ | -.01 | -.08 | -.06 | -.07 | -.01 | -.02 | -.24^**^ | -.42^**^ | -.03 | .06 | .12^**^ | -.58^**^ | -.68^**^ |  |  |  |  |  |  |
|  | [.07, .24] | [-.43, -.29] | [-.10, .07] | [-.16, .00] | [-.14, .03] | [-.16, .02] | [-.10, .07] | [-.11, .06] | [-.32, -.15] | [-.49, -.35] | [-.11, .06] | [-.03, .14] | [.04, .20] | [-.64, -.53] | [-.73, -.64] |  |  |  |  |  |  |
| 32. AtASM | -.06 | .21^**^ | -.02 | .06 | .05 | .05 | .03 | .01 | .20^**^ | .32^**^ | .04 | -.01 | -.12^**^ | .68^**^ | .73^**^ | -.64^**^ |  |  |  |  |  |
|  | [-.14, .03] | [.13, .29] | [-.11, .06] | [-.03, .14] | [-.04, .13] | [-.04, .14] | [-.06, .12] | [-.07, .10] | [.12, .28] | [.24, .39] | [-.04, .12] | [-.09, .08] | [-.21, -.04] | [.63, .72] | [.69, .77] | [-.69, -.59] |  |  |  |  |  |
| 33. NmASM | -.06 | .09^*^ | -.07 | .05 | .04 | .03 | .08 | .01 | .19^**^ | .21^**^ | .03 | .01 | .00 | .25^**^ | .27^**^ | -.28^**^ | .27^**^ |  |  |  |  |
|  | [-.14, .03] | [.01, .18] | [-.15, .02] | [-.03, .13] | [-.05, .12] | [-.05, .12] | [-.01, .16] | [-.07, .10] | [.10, .27] | [.13, .29] | [-.06, .11] | [-.07, .10] | [-.08, .09] | [.17, .33] | [.19, .35] | [-.36, -.20] | [.19, .35] |  |  |  |  |
| 34. HCar | .12^**^ | -.32^**^ | -.00 | -.03 | -.01 | .02 | -.03 | -.03 | -.26^**^ | -.44^**^ | -.06 | .11^*^ | .12^**^ | -.53^**^ | -.59^**^ | .73^**^ | -.56^**^ | -.19^**^ |  |  |  |
|  | [.03, .20] | [-.40, -.25] | [-.09, .08] | [-.12, .05] | [-.10, .07] | [-.07, .11] | [-.12, .05] | [-.11, .06] | [-.34, -.18] | [-.50, -.37] | [-.15, .02] | [.02, .19] | [.03, .20] | [-.59, -.47] | [-.64, -.53] | [.69, .77] | [-.61, -.50] | [-.27, -.11] |  |  |  |
| 35. HASM | -.10^*^ | .29^**^ | .02 | .07 | .05 | .06 | .06 | .02 | .21^**^ | .38^**^ | .10^*^ | .03 | -.09^*^ | .55^**^ | .60^**^ | -.63^**^ | .58^**^ | .29^**^ | -.56^**^ |  |  |
|  | [-.19, -.01] | [.21, .36] | [-.07, .10] | [-.02, .15] | [-.03, .14] | [-.03, .15] | [-.03, .14] | [-.06, .11] | [.13, .29] | [.30, .45] | [.02, .18] | [-.05, .12] | [-.18, -.01] | [.49, .61] | [.54, .65] | [-.68, -.58] | [.52, .64] | [.21, .37] | [-.62, -.50] |  |  |
| 36. HAASM | -.06 | .05 | -.00 | .09^*^ | .10^*^ | .11^*^ | -.02 | -.06 | .02 | .11^**^ | .04 | .00 | .07 | .12^**^ | .08^*^ | -.10^*^ | .06 | .11^*^ | -.07 | .18^**^ |  |
|  | [-.14, .03] | [-.03, .14] | [-.09, .08] | [.01, .18] | [.02, .19] | [.02, .19] | [-.11, .06] | [-.15, .02] | [-.07, .10] | [.03, .20] | [-.05, .12] | [-.08, .09] | [-.01, .16] | [.04, .21] | [.00, .17] | [-.18, -.01] | [-.03, .14] | [.02, .19] | [-.15, .02] | [.10, .26] |  |
|  |  |  |  |  |  |  |  |  |  |  |  |  |  |  |  |  |  |  |  |  |  |
| 37. IdEc | .01 | .08 | .00 | -.03 | -.04 | -.06 | .09^*^ | .08 | .08 | .18^**^ | -.00 | .10^*^ | -.06 | .20^**^ | .27^**^ | -.28^**^ | .27^**^ | .16^**^ | -.32^**^ | .26^**^ | .03 |
|  | [-.08, .09] | [-.00, .17] | [-.08, .08] | [-.12, .05] | [-.13, .04] | [-.15, .02] | [.01, .18] | [-.01, .16] | [-.01, .16] | [.09, .26] | [-.09, .08] | [.01, .18] | [-.15, .02] | [.11, .28] | [.19, .35] | [-.36, -.20] | [.19, .35] | [.08, .24] | [-.39, -.24] | [.18, .34] | [-.06, .11] |

*Note.* Values in square brackets indicate the 95% confidence interval for each correlation. bik= Biking, PT= Using public transport, Ed = Educationnal attainment, Inc = Incomes, %Wo = Percentage of work, Hou = Household number, Ch-12 = Number of children under 12 years, Ch+12 = Number of children of 12 years and older, Sur = Habitat surface, BikP = Possession of a bike , PtP = Possession of a transport pass , CarP = Number of cars in the household, Den = Density of the domicile, CarA= Accessibility by car, PtA = Proximity to a public transport stop, Mot = Principal motive of commuting, ChA = Frequency of simple trip chaining (2 activities), ChB= Frequency of medium trip chaining (3 or 4 activities), ChC= Frequency of complex trip chaining (chaining 5 or more activities in the same travel), Bio1= ASM during elementary school, Bio2= ASM during high school, Bio3= ASM during university, Bio4= ASM during first job, Mvpa= Moderate-to-vigorous PA, PhyH= Perceived physical health, InAm= Intention toward ASM, EfAm= Self-efficacy toward ASM, AtCar= Attitude toward car, AtAm= Attitude toward ASM, NAm= Social norms of ASM, HCar= Car habits, HAm= ASM habits, HSAm= Associated ASM habits, GI= Green identity. ^*^ indicates *p* < .05. ^**^ indicates *p* < .01.

Supplementary Table S3

*Description of the profiles of the participants in the focus groups and individual interviews.*

| **N** | **Participant** | **Age range** | **Sex** | **Profile** | **Current position** | **Profession** | **Sector of activity** | **Type of participation** | **Duration (in mins)** |
| --- | --- | --- | --- | --- | --- | --- | --- | --- | --- |
| 1 | EXPERT 1 | 21-30 | M | E | Ecomobility project manager in a metropolitan area | Geographer/urbanist | Public, corporate and planning policies | Interview | 45 |
| 2 | EXPERT 2 | 51-60 | W | E | Director of research in a government department | Anthropologist | Public, corporate and planning policies and research | Interview | 38 |
| 3 | EXPERT 3 | 31-40 | W | E | Active mobility consultant in a private company | Occupational therapist | Public, corporate and planning policies and research | Interview | 84 |
| 4 | EXPERT 4 | 21-30 | W | E | Mobility advisor in a metropolitan area | Unknown | Public policies | Interview | 48 |
| 5 | EXPERT 5 | 31-40 | M | E | Scientific collaborator in an university | Transport engineer/socioeconomist | Public, corporate and planning policies and research | Interview | 66 |
| 6 | EXPERT 6 | 41-50 | M | E | Researcher | Social psychologist | Research | Interview | 81 |
| 7 | EXPERT 7 | 31-40 | W | E | Mobility consultant in a private company | Urbanist | Public, corporate and planning policies and research | Interview | 51 |
| 8 | EXPERT 8 | 31-40 | W | E | Director of research in a public institution- behaviour change | Sociologist | Public, corporate and planning policies and research | Interview | 57 |
| 9 | EXPERT 9 | 61-70 | M | E | Member of an association for the promotion of active mobility | Retired geometer | Non-profit sector | *Focus group* | 100 |
| 10 | EXPERT 10 | 41-50 | M | E | Mobility/territory studies officer | Urbanist | Public, corporate and planning policies and research | *Focus group* | 100 |
| 11 | EXPERT 11 | 31-40 | M | E | Head of cycling policies in a metropolitan area | Engineer/urban planner | Public, corporate and planning policies and research | *Focus group* | 100 |
| 12 | EXPERT 12 | 31-40 | M | E | Car-sharing research officer | Urbanist | Public, corporate and planning policies and research | *Focus group* | 100 |
| 13 | CAR-DRIVER 1 | 41-50 | W | CCM | Humain resources responsable | Not necessary | Not necessary | *Focus group* | 73 |
| 14 | CAR-DRIVER 2 | 41-50 | W | CCM | Executive Assistant | Not necessary | Not necessary | *Focus group* | 73 |
| 15 | CAR-DRIVER 3 | 21-30 | W | CCM | Doctoral student | Not necessary | Not necessary | *Focus group* | 62 |
| 16 | CAR-DRIVER 4 | 61-70 | M | CCM | Retired | Not necessary | Not necessary | *Focus group* | 62 |
| 17 | CAR-DRIVER 5 | 21-30 | M | CCM | Survey officer | Not necessary | Not necessary | *Focus group* | 62 |

***Note.*** *N* = 17. Mins = minutes, M = Man, F = Women, E = Expert, ICCM = Individual willing to change his mobility

Supplementary Table S4

*Hierarchical regression models testing the independent association between the mobility-related, sociodemographic, and psychological factors and Biking and Walking (Hypothesis 1)*

|  | ***Model 1a*** | | | | | ***Model 1b*** | | | | |
| --- | --- | --- | --- | --- | --- | --- | --- | --- | --- | --- |
| **Variable** | ***b*** | **CI [LL, UL]** | ***SE b*** | **β** | ***p*** | ***b*** | **CI [LL, UL]** | ***SE b*** | **β** | ***p*** |
| (Constant) | -47.26 | [-91.23, -3.28] | 22.37 | — | .035 | 18.45 | [-32.66, 69.57] | 26.00 | — | .478 |
| Age | 0.33 | [ -0.10, 0.77] | 0.22 | .09 | .131 | 0.14 | [ -0.20, 0.49] | 0.18 | .04 | .414 |
| Gender | 2.90 | [ -4.15, 9.96] | 3.59 | .03 | .419 | -1.10 | [ -6.78, 4.58] | 2.89 | -.01 | .704 |
| Educational attainment | 2.75 | [ -1.24, 6.75] | 2.03 | .06 | .176 | 0.99 | [ -2.21, 4.19] | 1.63 | .02 | .544 |
| Level of incomes | -1.23 | [ -4.56, 2.10] | 1.69 | -.04 | .469 | -1.06 | [ -3.72, 1.60] | 1.35 | -.03 | .435 |
| Work percentage | 0.09 | [ -0.16, 0.34] | 0.13 | .03 | .493 | -0.11 | [ -0.31, 0.09] | 0.10 | -.04 | .283 |
| Number of persons in the household | 3.40 | [ -2.51, 9.31] | 3.00 | .10 | .258 | 2.45 | [ -2.28, 7.18] | 2.41 | .07 | .309 |
| Number of children under 12 years | -3.31 | [-10.76, 4.13] | 3.79 | -.06 | .382 | -4.52 | [-10.41, 1.38] | 3.00 | -.09 | .133 |
| Number of children of 12 years and older | -4.29 | [-10.03, 1.44] | 2.92 | -.08 | .142 | -5.17 | [ -9.68, -0.65] | 2.30 | -.10^*^ | .025 |
| Habitat surface | -0.05 | [ -0.16, 0.06] | 0.06 | -.05 | .417 | -0.02 | [ -0.10, 0.07] | 0.04 | -.02 | .689 |
| Possession of a bike | 9.08 | [6.74, 11.43] | 1.19 | .33^**^ | < .001 | 2.14 | [0.03, 4.24] | 1.07 | .08^*^ | .047 |
| Possession of a transport pass | -7.11 | [-10.13, -4.10] | 1.53 | -.20^**^ | < .001 | -8.03 | [-10.56, -5.50] | 1.29 | -.23^***^ | < .001 |
| Number of cars in the household | -12.26 | [-17.18, -7.34] | 2.50 | -.27^**^ | < .001 | -0.97 | [ -5.18, 3.25] | 2.14 | -.02 | .653 |
| Density of the domicile | 4.25e-04 | [0.00, 0.00] | 1.75e-04 | .12^*^ | .016 | 7.85e-05 | [ -0.00, 0.00] | 1.41e-04 | .02 | .578 |
| Accessibility by car | 2.05 | [ -2.71, 6.81] | 2.42 | .04 | .398 | 3.98 | [0.16, 7.80] | 1.94 | .07^*^ | .041 |
| Proximity to a public transport stop | 9.37 | [3.38, 15.36] | 3.05 | .14^**^ | .002 | 3.20 | [ -1.67, 8.07] | 2.48 | .05 | .197 |
| Principal motive: purchases | -2.92 | [-22.60, 16.75] | 10.01 | -.01 | .770 | -1.76 | [-17.25, 13.74] | 7.88 | -.01 | .824 |
| Principal motive: accompany someone | 4.24 | [-21.92, 30.39] | 13.31 | .01 | .750 | 19.93 | [ -0.78, 40.65] | 10.54 | .06 | .059 |
| Principal motive: « other » | -10.48 | [-27.77, 6.81] | 8.79 | -.05 | .234 | -11.54 | [-25.28, 2.21] | 6.99 | -.05 | .100 |
| Frequency of simple trip chaining | -0.02 | [ -5.69, 5.65] | 2.88 | -.00 | .994 | 1.64 | [ -2.82, 6.11] | 2.27 | .03 | .470 |
| Frequency of medium trip chaining | -2.99 | [ -9.73, 3.76] | 3.43 | -.06 | .385 | -6.15 | [-11.46, -0.85] | 2.70 | -.12^*^ | .023 |
| Frequency of complex trip chaining | 7.48 | [ -0.26, 15.22] | 3.94 | .11 | .058 | 6.93 | [0.82, 13.03] | 3.10 | .10^*^ | .026 |
| ASM during elementary school | -0.32 | [ -4.40, 3.77] | 2.08 | -.01 | .878 | -2.41 | [ -5.68, 0.86] | 1.66 | -.05 | .149 |
| ASM during high school | 0.71 | [ -5.12, 6.54] | 2.96 | .01 | .811 | 1.30 | [ -3.30, 5.90] | 2.34 | .02 | .578 |
| ASM during university | 5.94 | [0.26, 11.62] | 2.89 | .09^*^ | .041 | 0.55 | [ -4.03, 5.12] | 2.33 | .01 | .814 |
| ASM during first job | 2.26 | [ -2.41, 6.94] | 2.38 | .05 | .342 | -0.54 | [ -4.29, 3.21] | 1.91 | -.01 | .779 |
| Moderate-to-vigorous PA |  |  |  |  |  | 6.81e-04 | [ -0.00, 0.01] | 0.003 | .01 | .808 |
| Perceived physical health |  |  |  |  |  | 9.74 | [1.13, 18.36] | 4.38 | .08^*^ | .027 |
| Perceived risks of getting COVID-19 |  |  |  |  |  | 1.68 | [ -0.70, 4.05] | 1.21 | .05 | .165 |
| Intention toward ASM |  |  |  |  |  | 0.20 | [ -1.59, 1.98] | 0.91 | .01 | .829 |
| Self-efficacy toward ASM |  |  |  |  |  | 1.80 | [ -0.08, 3.67] | 0.95 | .10 | .060 |
| Attitude toward car |  |  |  |  |  | -7.28 | [ -9.70, -4.86] | 1.23 | -.33 | < .001 |
| Attitude toward ASM |  |  |  |  |  | 4.01 | [1.59, 6.42] | 1.23 | .17^***^ | .001 |
| Social norms of ASM |  |  |  |  |  | 1.74 | [ -0.54, 4.03] | 1.16 | .05 | .135 |
| Car habits |  |  |  |  |  | -3.34 | [ -5.76, -0.93] | 1.23 | -.14^**^ | .007 |
| ASM habits |  |  |  |  |  | 1.84 | [0.07, 3.61] | 0.90 | .10^*^ | .042 |
| Associated ASM habits |  |  |  |  |  | -8.37 | [-11.76, -4.99] | 1.72 | -.18^***^ | < .001 |
| Green identity |  |  |  |  |  | -1.26 | [ -4.44, 1.92] | 1.62 | -.03 | .437 |
| **R²** | 0.35 |  |  |  |  |  | 0.61 |  |  |  |
| **Adjusted R²** | 0.31 |  |  |  |  |  | 0.58 |  |  |  |
| **Durbin–Watson** | 2.02 |  |  |  |  |  | 1.88 |  |  |  |

Note. * p < .05, ** p < .01, *** p < .001. b = unstandardized; β = standardized, CI = Confidence Interval; LL = Lower Limit; UL = Upper Limit..*N*_Model 1_= 426, *N*_Model 2_= 426. Dependent variable is percentage of active and sustainable mobility in a typical week. ASM = Active and sustainable mobility, PA = Physical activity. Women and work/study were used as reference dummy group for gender and principal motive. Values between brackets represent confidence intervals.

Supplementary Table S5

*Hierarchical regression models testing the independent association between the mobility-related, sociodemographic, and psychological factors and using Public Transport (Hypothesis 1)*

|  | ***Model 2a*** | | | | | ***Model 2b*** | | | | |
| --- | --- | --- | --- | --- | --- | --- | --- | --- | --- | --- |
| **Variable** | ***b*** | **CI [LL, UL]** | ***SE b*** | **β** | ***p*** | ***b*** | **CI [LL, UL]** | ***SE b*** | **β** | ***p*** |
| (Constant) | -3.41 | [-35.39, 28.58] | 16.27 |  | .834 | -14.07 | [-59.53, 31.38] | 23.12 |  | .543 |
| Age | -0.02 | [ -0.34, 0.29] | 0.16 | -.01 | .884 | 0.07 | [ -0.24, 0.38] | 0.16 | .02 | .642 |
| Gender | 1.56 | [ -3.57, 6.70] | 2.61 | .02 | .549 | 1.77 | [ -3.28, 6.82] | 2.57 | .03 | .492 |
| Educational attainment | 0.08 | [ -2.83, 2.99] | 1.48 | .00 | .957 | -0.80 | [ -3.65, 2.04] | 1.45 | -.02 | .580 |
| Level of incomes | 0.96 | [ -1.46, 3.39] | 1.23 | .04 | .434 | 0.47 | [ -1.90, 2.83] | 1.20 | .02 | .698 |
| Work percentage | -0.06 | [ -0.24, 0.12] | 0.09 | -.03 | .501 | -0.04 | [ -0.22, 0.14] | 0.09 | -.02 | .647 |
| Number of persons in the household | -1.50 | [ -5.80, 2.80] | 2.19 | -.06 | .493 | -0.92 | [ -5.12, 3.29] | 2.14 | -.03 | .669 |
| Number of children under 12 years | 3.46 | [ -1.95, 8.87] | 2.75 | .08 | .210 | 2.24 | [ -3.01, 7.48] | 2.67 | .05 | .402 |
| Number of children of 12 years and older | 1.34 | [ -2.83, 5.51] | 2.12 | .03 | .529 | 0.89 | [ -3.13, 4.91] | 2.04 | .02 | .662 |
| Habitat surface | -0.03 | [ -0.11, 0.05] | 0.04 | -.04 | .526 | -0.01 | [ -0.08, 0.07] | 0.04 | -.01 | .877 |
| Possession of a bike | -1.17 | [ -2.88, 0.53] | 0.87 | -.05 | .178 | -1.48 | [ -3.35, 0.39] | 0.95 | -.07 | .122 |
| Possession of a transport pass | 17.41 | [15.22, 19.61] | 1.12 | .62^***^ | < .001 | 15.04 | [12.79, 17.29] | 1.14 | .53^***^ | < .001 |
| Number of cars in the household | 1.53 | [ -2.04, 5.11] | 1.82 | .04 | .400 | 2.94 | [ -0.81, 6.69] | 1.91 | .08 | .124 |
| Density of the domicile | 0.0001 | [ -0.00, 0.00] | 0.0001 | .04 | .390 | 0.0001 | [ -0.00, 0.00] | 0.0001 | .04 | .330 |
| Accessibility by car | -2.95 | [ -6.41, 0.51] | 1.76 | -.07 | .094 | -2.89 | [ -6.28, 0.51] | 1.73 | -.07 | .096 |
| Proximity to a public transport stop | 0.52 | [ -3.84, 4.88] | 2.22 | .01 | .815 | -0.51 | [ -4.84, 3.82] | 2.20 | -.01 | .818 |
| Principal motive: purchases | 11.78 | [ -2.53, 26.10] | 7.28 | .06 | .106 | 10.68 | [ -3.10, 24.45] | 7.01 | .06 | .128 |
| Principal motive: accompany someone | -5.25 | [-24.28, 13.78] | 9.68 | -.02 | .588 | -6.90 | [-25.32, 11.51] | 9.37 | -.03 | .462 |
| Principal motive: « other » | -7.71 | [-20.29, 4.86] | 6.40 | -.05 | .229 | -6.45 | [-18.68, 5.77] | 6.22 | -.04 | .300 |
| Frequency of simple trip chaining | -1.63 | [ -5.75, 2.50] | 2.10 | -.04 | .439 | -1.83 | [ -5.81, 2.14] | 2.02 | -.04 | .364 |
| Frequency of medium trip chaining | 5.61 | [0.70, 10.51] | 2.50 | .14^*^ | .025 | 4.83 | [0.12, 9.55] | 2.40 | .12^*^ | .045 |
| Frequency of complex trip chaining | -7.53 | [-13.15, -1.90] | 2.86 | -.13^**^ | .009 | -8.01 | [-13.44, -2.58] | 2.76 | -.14^**^ | .004 |
| ASM during elementary school | 0.30 | [ -2.67, 3.28] | 1.51 | .01 | .840 | -0.37 | [ -3.27, 2.54] | 1.48 | -.01 | .805 |
| ASM during high school | 1.27 | [ -2.97, 5.51] | 2.16 | .02 | .557 | 1.20 | [ -2.89, 5.29] | 2.08 | .02 | .565 |
| ASM during university | -1.98 | [ -6.11, 2.15] | 2.10 | -.04 | .347 | -0.52 | [ -4.59, 3.55] | 2.07 | -.01 | .801 |
| ASM during first job | 5.06 | [1.66, 8.46] | 1.73 | .13^**^ | .004 | 3.78 | [0.45, 7.12] | 1.70 | .10^*^ | .026 |
| Moderate-to-vigorous PA |  |  |  |  |  | -2.31e-03 | [ -0.01, 0.00] | 0.003 | -.03 | .356 |
| Perceived physical health |  |  |  |  |  | -0.15 | [ -7.81, 7.52] | 3.90 | .00 | .970 |
| Perceived risks of getting COVID-19 |  |  |  |  |  | -1.42 | [ -3.53, 0.69] | 1.07 | -.05 | .187 |
| Intention toward ASM |  |  |  |  |  | 1.68 | [0.10, 3.26] | 0.81 | .11^*^ | .038 |
| Self-efficacy toward ASM |  |  |  |  |  | 0.06 | [ -1.61, 1.72] | 0.85 | .00 | .944 |
| Attitude toward car |  |  |  |  |  | -1.71 | [ -3.86, 0.45] | 1.10 | -.09 | .120 |
| Attitude toward ASM |  |  |  |  |  | -2.41 | [ -4.56, -0.26] | 1.09 | -.13^*^ | .028 |
| Social norms of ASM |  |  |  |  |  | -0.24 | [ -2.28, 1.79] | 1.03 | -.01 | .815 |
| Car habits |  |  |  |  |  | 1.04 | [ -1.10, 3.19] | 1.09 | .05 | .340 |
| ASM habits |  |  |  |  |  | 0.89 | [ -0.68, 2.47] | 0.80 | .06 | .264 |
| Associated ASM habits |  |  |  |  |  | 8.29 | [5.28, 11.29] | 1.53 | .22^***^ | < .001 |
| Green identity |  |  |  |  |  | 1.33 | [ -1.50, 4.16] | 1.44 | .04 | .356 |
| **R²** | 0.47 |  |  |  |  | 0.53 |  |  |  |  |
| **Adjusted R²** | 0.44 |  |  |  |  | 0.49 |  |  |  |  |
| **Durbin–Watson** | 1.77 |  |  |  |  | 1.72 |  |  |  |  |

Note. * p < .05, ** p < .01, *** p < .001. b = unstandardized; β = standardized, CI = Confidence Interval; LL = Lower Limit; UL = Upper Limit..*N*_Model 1_= 426, *N*_Model 2_= 426. Dependent variable is percentage of active and sustainable mobility in a typical week. ASM = Active and sustainable mobility, PA = Physical activity. Women and work/study were used as reference dummy group for gender and principal motive. Values between brackets represent confidence intervals.

Supplementary Table S6

*Hierarchical regression models testing if the association between the mobility-related and sociodemographic factors and active and sustainable mobility is mediated by psychological factors (Hypothesis 2)*

|  | **Model 3a - Biking** | | | | | **Model 3b – Public Transport** | | | | | **Model 3.2 - Intention** | | | | | **Model 3.3 – Self efficacy** | | | | |
| --- | --- | --- | --- | --- | --- | --- | --- | --- | --- | --- | --- | --- | --- | --- | --- | --- | --- | --- | --- | --- |
|  | ***b*** | ***CI* [LL, UL]** | ***SE* b** | ***β*** | ***p*** | ***b*** | ***CI* [LL, UL]** | ***SE* b** | ***β*** | ***p*** | ***b*** | ***CI* [LL, UL]** | ***SE* b** | ***β*** | ***p*** | ***b*** | ***CI* [LL, UL]** | ***SE* b** | ***β*** | ***p*** |
| (Constant) | 18.43 | [-32.81, 69.66] | 26.06 |  | .480 | -12.94 | [-58.54, 32.65] | 23.19 |  | .577 | 0.68 | [-2.28, 3.63] | 1.50 |  | .654 | -0.09 | [-2.90, 2.72] | 1.43 |  | .950 |
| Age | 0.15 | [-0.19, 0.50] | 0.18 | .04 | .387 | 0.09 | [-0.22, 0.40] | 0.16 | .03 | .554 | 0.01 | [-0.01, 0.03] | 0.01 | .06 | .244 | 3.62e-03 | [-0.02, 0.02] | 9.73e-03 | .02 | .710 |
| Gender (man) | -0.63 | [-6.29, 5.03] | 2.88 | -.01 | .827 | 1.55 | [-3.49, 6.59] | 2.56 | .02 | .546 | -0.14 | [-0.47, 0.19] | 0.17 | -.03 | .399 | 0.28 | [-0.04, 0.59] | 0.16 | .06 | .082 |
| Educational attainment | 1.15 | [-2.05, 4.35] | 1.63 | .03 | .479 | -0.57 | [-3.41, 2.28] | 1.45 | -.02 | .696 | 0.14 | [-0.05, 0.32] | 0.09 | .06 | .143 | 0.08 | [-0.10, 0.25] | 0.09 | .03 | .398 |
| Level of incomes | -1.05 | [-3.72, 1.61] | 1.36 | -.03 | .438 | 0.39 | [-1.99, 2.76] | 1.21 | .02 | .749 | -0.05 | [-0.20, 0.11] | 0.08 | -.03 | .539 | 7.06e-03 | [-0.14, 0.15] | 0.07 | .00 | .925 |
| Work percentage | -0.11 | [-0.30, 0.09] | 0.10 | -.04 | .298 | -0.04 | [-0.22, 0.14] | 0.09 | -.02 | .664 | 1.18e-03 | [-0.01, 0.01] | 5.84e-03 | .01 | .839 | 1.59e-03 | [-0.01, 0.01] | 5.56e-03 | .01 | .775 |
| Number of persons in the household | 1.81 | [-2.89, 6.51] | 2.39 | .06 | .449 | -1.06 | [-5.24, 3.12] | 2.13 | -.04 | .618 | -0.07 | [-0.35, 0.20] | 0.14 | -.04 | .592 | -0.35 | [-0.61, -0.09] | 0.13 | -.19 | .008 |
| Number of children under 12 years | -3.88 | [-9.75, 2.00] | 2.99 | -.07 | .195 | 2.37 | [-2.86, 7.60] | 2.66 | .06 | .374 | 0.07 | [-0.27, 0.40] | 0.17 | .02 | .705 | 0.35 | [0.03, 0.67] | 0.16 | .12 | .035 |
| Number of children of 12 years and older | -4.87 | [-9.39, -0.34] | 2.30 | -.10 | .035 | 1.06 | [-2.96, 5.09] | 2.05 | .03 | .603 | 0.10 | [-0.16, 0.36] | 0.13 | .03 | .467 | 0.16 | [-0.09, 0.40] | 0.13 | .06 | .217 |
| Habitat surface | -0.01 | [-0.10, 0.07] | 0.04 | -.02 | .749 | -0.01 | [-0.09, 0.06] | 0.04 | -.02 | .715 | -4.99e-03 | [-0.01, 0.00] | 2.55e-03 | -.10 | .051 | 2.55e-03 | [0.00, 0.01] | 2.42e-03 | .05 | .293 |
| Possession of a bike | 2.13 | [0.04, 4.23] | 1.07 | .08 | .046 | -1.25 | [-3.11, 0.62] | 0.95 | -.06 | .189 | 0.14 | [0.02, 0.26] | 0.06 | .09 | .027 | -0.02 | [-0.13, 0.10] | 0.06 | -.01 | .779 |
| Possession of a transport pass | -7.83 | [-10.36, -5.31] | 1.28 | -.22 | <.001 | 15.29 | [13.04, 17.53] | 1.14 | .54 | <.001 | 0.15 | [0.00, 0.29] | 0.07 | .08 | .049 | 0.09 | [-0.05, 0.23] | 0.07 | .05 | .199 |
| Number of cars in the household | -0.72 | [-4.93, 3.50] | 2.14 | -.02 | .738 | 3.24 | [-0.51, 6.99] | 1.91 | .09 | .091 | 0.17 | [-0.07, 0.42] | 0.12 | .07 | .161 | 0.12 | [-0.11, 0.35] | 0.12 | .05 | .310 |
| Density of the domicile | 0 | [-0.00, 0.00] | 1.41e-04 | .03 | .521 | 0 | [-0.00, 0.00] | 0 | .05 | .264 | 0 | [0.00, 0.00] | 8.15e-06 | .05 | .195 | 0 | [0.00, 0.00] | 0 | .03 | .466 |
| Accessibility by car | 3.83 | [0.04, 7.62] | 1.93 | .07 | .048 | -2.45 | [-5.83, 0.92] | 1.72 | -.06 | .153 | 0.26 | [0.04, 0.48] | 0.11 | .09 | .020 | -0.11 | [-0.32, 0.10] | 0.11 | -.04 | .289 |
| Proximity to a public transport stop | 3.45 | [-1.43, 8.32] | 2.48 | .05 | .165 | -0.59 | [-4.93, 3.75] | 2.21 | -.01 | .789 | -0.06 | [-0.34, 0.23] | 0.14 | -.02 | .700 | 0.14 | [-0.12, 0.41] | 0.14 | .04 | .295 |
| Principal motive: purchases | -2.32 | [-17.84, 13.20] | 7.90 | -.01 | .769 | 10.42 | [-3.39, 24.24] | 7.03 | .05 | .139 | -0.14 | [-1.04, 0.76] | 0.46 | -.01 | .757 | -0.30 | [-1.15, 0.55] | 0.43 | -.02 | .493 |
| Principal motive: accompany someone | 20.15 | [-0.56, 40.85] | 10.53 | .06 | .056 | -5.38 | [-23.81, 13.05] | 9.37 | -.02 | .566 | 0.91 | [-0.29, 2.10] | 0.61 | .05 | .137 | 0.02 | [-1.12, 1.16] | 0.58 | .00 | .972 |
| Principal motive: « other » | -11.18 | [-24.83, 2.48] | 6.94 | -.05 | .108 | -7.99 | [-20.14, 4.16] | 6.18 | -.05 | .197 | -0.93 | [-1.72, -0.14] | 0.40 | -.08 | .021 | 0.30 | [-0.45, 1.05] | 0.38 | .03 | .428 |
| Frequency of simple trip chaining | 1.59 | [-2.88, 6.07] | 2.28 | .03 | .484 | -1.92 | [-5.91, 2.06] | 2.03 | -.05 | .344 | -0.05 | [-0.31, 0.21] | 0.13 | -.02 | .697 | -0.02 | [-0.27, 0.22] | 0.13 | -.01 | .860 |
| Frequency of medium trip chaining | -6.04 | [-11.35, -0.73] | 2.70 | -.12 | .026 | 5.11 | [0.38, 9.83] | 2.40 | .12 | .034 | 0.16 | [-0.15, 0.47] | 0.16 | .06 | .302 | 0.05 | [-0.25, 0.34] | 0.15 | .02 | .753 |
| Frequency of complex trip chaining | 6.59 | [0.49, 12.69] | 3.10 | .09 | .034 | -8.49 | [-13.92, -3.06] | 2.76 | -.15 | .002 | -0.28 | [-0.63, 0.07] | 0.18 | -.07 | .117 | -0.16 | [-0.49, 0.18] | 0.17 | -.04 | .355 |
| ASM during elementary school | -2.67 | [-5.94, 0.60] | 1.66 | -.06 | .109 | -0.37 | [-3.28, 2.54] | 1.48 | -.01 | .804 | 3.36e-03 | [-0.19, 0.19] | 0.10 | .00 | .972 | -0.15 | [-0.33, 0.03] | 0.09 | -.06 | .111 |
| ASM during high school | 1.59 | [-3.01, 6.20] | 2.34 | .02 | .496 | 1.39 | [-2.71, 5.49] | 2.08 | .03 | .506 | 0.11 | [-0.16, 0.37] | 0.14 | .03 | .432 | 0.15 | [-0.10, 0.40] | 0.13 | .04 | .240 |
| ASM during university | 0.28 | [-4.30, 4.86] | 2.33 | .004 | .905 | -0.48 | [-4.55, 3.60] | 2.07 | -.01 | .818 | 0.03 | [-0.23, 0.30] | 0.13 | .01 | .814 | -0.15 | [-0.41, 0.10] | 0.13 | -.04 | .229 |
| ASM during first job | -0.20 | [-3.94, 3.54] | 1.90 | -.004 | .916 | 4.05 | [0.72, 7.38] | 1.69 | .10 | .017 | 0.15 | [-0.06, 0.37] | 0.11 | .06 | .160 | 0.17 | [-0.04, 0.38] | 0.10 | .06 | .106 |
| Moderate-to-vigorous PA | 3.33e-04 | [-0.01, 0.01] | 2.81e-03 | .004 | .906 | -2.64e-03 | [-0.01, 0.00] | 2.50e-03 | -.04 | .291 | -1.93e-04 | [0.00, 0.00] | 1.62e-04 | -.04 | .234 | -1.73e-04 | [0.00, 0.00] | 1.54e-04 | -.04 | .263 |
| Perceived physical health | 10.20 | [1.58, 18.81] | 4.38 | .08 | .020 | -0.42 | [-8.08, 7.25] | 3.90 | -.004 | .915 | -0.17 | [-0.67, 0.33] | 0.25 | -.03 | .499 | 0.27 | [-0.20, 0.75] | 0.24 | .04 | .259 |
| Perceived risks of getting COVID-19 | 1.64 | [-0.73, 4.02] | 1.21 | .05 | .174 | -1.30 | [-3.41, 0.81] | 1.07 | -.05 | .227 | 0.07 | [-0.07, 0.21] | 0.07 | .04 | .303 | -0.03 | [-0.16, 0.10] | 0.07 | -.01 | .683 |
| Attitude toward car | -7.74 | [-10.13, -5.36] | 1.21 | -.35 | <.001 | -1.97 | [-4.09, 0.15] | 1.08 | -.11 | .069 | -0.15 | [-0.29, -0.01] | 0.07 | -.12 | .034 | -0.24 | [-0.37, -0.11] | 0.07 | -.19 | <.001 |
| Attitude toward ASM | 5.24 | [3.24, 7.25] | 1.02 | .23 | <.001 | -1.38 | [-3.17, 0.41] | 0.91 | -.08 | .130 | 0.59 | [0.48, 0.71] | 0.06 | .47 | <.001 | 0.63 | [0.52, 0.74] | 0.06 | .49 | <.001 |
| Social norms of ASM | 1.84 | [-0.46, 4.13] | 1.17 | .06 | .116 | -0.15 | [-2.19, 1.89] | 1.04 | -.01 | .884 | 0.05 | [-0.08, 0.19] | 0.07 | .03 | .431 | 0.04 | [-0.08, 0.17] | 0.06 | .02 | .483 |
| Car habits | -3.44 | [-5.86, -1.03] | 1.23 | -.14 | .005 | 0.90 | [-1.25, 3.05] | 1.09 | .05 | .412 | -0.09 | [-0.22, 0.05] | 0.07 | -.06 | .232 | -0.05 | [-0.18, 0.09] | 0.07 | -.03 | .490 |
| ASM habits | 2.11 | [0.36, 3.85] | 0.89 | .11 | .018 | 1.16 | [-0.39, 2.71] | 0.79 | .08 | .142 | 0.16 | [0.05, 0.26] | 0.05 | .15 | .003 | 0.13 | [0.04, 0.23] | 0.05 | .12 | .007 |
| Associated ASM habits | -8.35 | [-11.74, -4.97] | 1.72 | -.18 | <.001 | 8.49 | [5.47, 11.50] | 1.53 | .22 | <.001 | 0.12 | [-0.08, 0.31] | 0.10 | .05 | .230 | 3.46e-05 | [-0.19, 0.19] | 0.09 | .00 | > .999 |
| Green identity | -1.14 | [-4.30, 2.01] | 1.61 | -.03 | .477 | 0.96 | [-1.85, 3.77] | 1.43 | .03 | .501 | -0.22 | [-0.40, -0.04] | 0.09 | -.09 | .017 | 0.09 | [-0.08, 0.26] | 0.09 | .05 | .312 |
| ***R*^2^** | 0.61 |  |  |  |  | 0.52 |  |  |  |  | 0.57 |  |  |  |  | 0.63 |  |  |  |  |
| **Adjusted *R*^2^** | 0.58 |  |  |  |  | 0.48 |  |  |  |  | 0.53 |  |  |  |  | 0.59 |  |  |  |  |
| **Durbin Watson** | 1.88 |  |  |  |  | 1.72 |  |  |  |  | 1.96 |  |  |  |  | 2.03 |  |  |  |  |

Note: N_Model 3a_= 426, N_Model 3b_= 426, N_Model 3.2_= 426, N_Model 3.3_= 426. Dependent variable in model 3a was percentage of biking and walking, dependent variable in model 3b was using public transport, dependent variable in model 3.2 was intention, dependent variable in model 3.3 was self-efficacy. ASM = Active and sustainable mobility, PA = Physical activity. Women and work/study were used as reference dummy group for gender and principal motive. b = raw coefficient, SE b= Standard error of betas, β = Standardized betas, t represents p <.10, * p < .05, ** p < .01, *** p <.001. Values between brackets represent confidence intervals.

Supplementary Table S7

Indirect effects of mobility-related variables on using public transport through psychological variables (Hypothesis 2)

| **Indirect effects of possession of a bike through intention** | | | | |
| --- | --- | --- | --- | --- |
| **Path** | ***b*** | ***SE* b** | ***t*** | ***p*** |
| a | 0.40 | 0.065 | *t*(536) 6.15 | <.001^***^ |
| b | 3.99^***^ | 0.61 | *t*(535) 6.55 | <.001^***^ |
| c | -3.41^**^ | 0.95 | *t*(536) 3.59 | <.001^**^ |
| c’ | -5.01^***^ | 0.95 | *t*(535) 5.28 | <.001^***^ |
| Indirect Effect | 1.59 [0.92 ; 2.35] |  |  |  |
| **Indirect effects of possession of transport pass through intention** | | | | |
| **Path** | ***b*** | ***SE* b** | ***t*** | ***p*** |
| a | 0.39^***^ | 0.08 | *t*(536) 4.74 | <.001^***^ |
| b | 1.34^**^ | 0.48 | *t*(535) 2.82 | .005^**^ |
| c | 18.33^***^ | 0.92 | *t*(536) 20.02 | <.001^***^ |
| c’ | 17.81^**^ | 0.93 | *t*(535) 19.17 | <.001^***^ |
| Indirect Effect | 0.53 [0.15 ; 1.00] |  |  |  |
| **Indirect effects of accessibility by car through intention** | | | | |
| **Path** | ***b*** | ***SE* b** | ***t*** | ***p*** |
| a | -0.03 | 0.13 | *t*(507) 0.20 | .839 |
| b | 2.91^***^ | 0.61 | *t*(506) 4.81 | <.001^***^ |
| c | -6.21^***^ | 1.86 | *t*(507) 3.34 | <.001^***^ |
| c’ | -6.13^***^ | 1.82 | *t*(506) 3.36 | <.001^***^ |
| Indirect Effect | -0.08 [0.89 ; 0.70] |  |  |  |

*Note:* Coefficients are unstandardized regression coefficients. a=path between the predictor and the mediator, b= path between the mediator and the dependent variable, c= path between the predictor and the dependent variable, c’= mediated path between the predictor and the dependent variable, b= unstandardized regression coefficient, SE = standard error, t= t-values.

Supplementary Table S8

*Stepwise regression model testing if the association between the mobility-related and sociodemographic factors and active and biking/walking is moderated by psychological factors (Hypothesis 3)*

| **Model 4a: Stepwise regression for biking/walking** | | | | | | |
| --- | --- | --- | --- | --- | --- | --- |
|  | ***b*** | **LL** | **UL** | ***SE* b** | ***β*** | ***p*** |
| (Constant) | 49.47 | 45.48 | 53.47 | 2.03 |  | <.001 |
| Attitude toward car | -6.99 | -9.36 | -4.63 | 1.20 | -.0313 | <.001 |
| Possession of a transport pass | -6.71 | -9.31 | -4.11 | 1.32 | -.0192 | <.001 |
| Attitude toward ASM | 4.48 | 2.10 | 6.86 | 1.21 | .0195 | <.001 |
| Associated ASM habits | -7.78 | -11.12 | -4.45 | 1.70 | -.0164 | <.001 |
| Possession of a bike | 2.97 | 0.82 | 5.12 | 1.09 | .0107 | .0007 |
| Frequency of complex trip chaining | 6.32 | 0.38 | 12.26 | 3.02 | .0089 | .0037 |
| ASM habits | -3.24 | -5.62 | -0.86 | 1.21 | -.0131 | .0008 |
| ASM during high school | 0.74 | -3.77 | 5.25 | 2.29 | .0011 | .0747 |
| Self-efficacy towards ASM | 1.34 | -0.50 | 3.19 | 0.94 | .0075 | .0153 |
| Proximity to a public transport stop | 3.04 | -1.70 | 7.79 | 2.41 | .0045 | .0208 |
| Social norms of ASM | 1.41 | -0.83 | 3.66 | 1.14 | .0043 | .0217 |
| Age | 0.10 | -0.24 | 0.45 | 0.17 | .0027 | .0552 |
| ASM habits | 1.80 | 0.05 | 3.55 | 0.89 | .0093 | .0044 |
| Educational attainment | 0.48 | -2.70 | 3.65 | 1.62 | .001 | .0768 |
| Perceived risks of getting COVID-19 | 1.30 | -1.03 | 3.64 | 1.19 | .0037 | .0273 |
| Intention towards ASM | 1.11 | -1.07 | 3.29 | 1.11 | .0061 | .0318 |
| Frequency of simple trip chaining | 2.01 | -2.41 | 6.43 | 2.25 | .004 | .0372 |
| Work percentage | -0.06 | -0.25 | 0.14 | 0.10 | -.002 | .0566 |
| Number of cars in the household | -1.13 | -5.25 | 2.99 | 2.09 | -.0025 | .0590 |
| ASM during elementary school | -3.00 | -6.21 | 0.22 | 1.64 | -.0064 | .0068 |
| Green identity | -0.96 | -4.08 | 2.16 | 1.59 | -.0022 | .0546 |
| Gender (man) | 0.20 | -5.40 | 5.81 | 2.85 | .0002 | .0943 |
| Number of children under 12 years | -3.56 | -9.39 | 2.28 | 2.97 | -.0067 | .0232 |
| Number of persons in the household | 1.81 | -2.87 | 6.48 | 2.38 | .0054 | .0447 |
| Habitat surface | -0.01 | -0.09 | 0.08 | 0.04 | -.0007 | .0891 |
| Level of incomes | -1.15 | -3.81 | 1.51 | 1.35 | -.0038 | .0396 |
| Moderate-to-vigorous PA | 0.00 | -0.01 | 0.01 | 0.00 | .0002 | .0960 |
| Density of the domicile | 0.00 | 0.00 | 0.00 | 0.00 | .0012 | .0739 |
| Frequency of medium trip chaining | -5.03 | -10.27 | 0.22 | 2.67 | -.0098 | .0060 |
| ASM during university | 1.76 | -2.79 | 6.30 | 2.31 | .0028 | .0448 |
| Perceived physical health | 7.43 | -1.05 | 15.90 | 4.31 | .0059 | .0086 |
| Principal motive: purchases | 1.87 | -13.33 | 17.07 | 7.73 | .0008 | .0809 |
| Principal motive: accompany someone | 18.59 | -1.66 | 38.85 | 10.30 | .0059 | .0072 |
| Principal motive: « other » | -9.16 | -22.86 | 4.54 | 6.97 | -.0043 | .0189 |
| Number of children of 12 years and older | -4.39 | -8.84 | 0.05 | 2.26 | -.0086 | .0053 |
| ASM during first job | -0.63 | -4.30 | 3.04 | 1.87 | -.0013 | .0736 |
| Accessibility by car | 3.09 | -0.66 | 6.83 | 1.90 | .0057 | .0106 |
| Possession of a bike x Intention | 1.33 | 0.58 | 2.08 | 0.38 | .0123 | .0001 |
| Possession of a transport pass x Intention | -1.19 | -2.36 | -0.01 | 0.60 | -.0073 | .0048 |
| Principal motive: purchases x Intention | -6.50 | -13.13 | 0.13 | 3.37 | -.0065 | .0054 |
| Principal motive: accompany someone x Intention | -9.05 | -18.56 | 0.45 | 4.83 | -.006 | .0062 |
| Principal motive: « other » x Intention | -1.14 | -6.91 | 4.63 | 2.94 | -.0013 | .0699 |
| Number of cars in the household x Intention | 0.70 | -0.65 | 2.04 | 0.68 | .0037 | .0309 |
| ASM during elementary school x Intention | 0.90 | -0.34 | 2.15 | 0.63 | .0046 | .0155 |
| Gender (man) x Intention | -1.33 | -3.64 | 0.98 | 1.18 | -.0051 | .0257 |
| Level of incomes x Intention | 0.53 | -0.41 | 1.47 | 0.48 | .0039 | .0268 |
| **R^2^** | 0.64 |  |  |  |  |  |
| **Adjusted R^2^** | 0.60 |  |  |  |  |  |

Note: N_Model 4a_= 426. Dependent variable is percentage of active and sustainable mobility in a typical week. ASM = Active and sustainable mobility, PA = Physical activity. Women and work/study were used as reference dummy group for gender and principal motive. *b* = raw coefficient, *SE* *b*= Standard error of betas, *β* = Standardized betas, ^t^ represents p <.10, ^*^ p < .05, ^**^ p < .01, ^***^ p <.001. Values between brackets represent confidence interval.

Supplementary Table S9

*Stepwise regression model testing if the association between the mobility-related and sociodemographic factors and active and using public transport is moderated by psychological factors (Hypothesis 3)*

| **Model 4b: Stepwise regression for using public transport** | | | | | | |
| --- | --- | --- | --- | --- | --- | --- |
|  | ***b*** | **LL** | **UL** | ***SE* b** | ***β*** | ***p*** |
| (Constant) | 22.55 | 18.98 | 26.12 | 1.82 |  | .000 |
| Possession of a transport pass | 13.87 | 11.51 | 16.22 | 1.20 | .49 | .000 |
| Associated ASM habits | 7.77 | 4.79 | 10.75 | 1.52 | .20 | .000 |
| ASM during first job | 3.64 | 0.34 | 6.94 | 1.68 | .09 | .031 |
| Frequency of complex trip chaining | -8.07 | -13.44 | -2.69 | 2.73 | -.14 | .003 |
| Possession of a bike | -2.01 | -3.95 | -0.07 | 0.99 | -.09 | .043 |
| ASM during high school | 1.70 | -2.35 | 5.75 | 2.06 | .03 | .410 |
| Principal motive: purchases | 10.77 | -2.83 | 24.37 | 6.92 | .06 | .120 |
| Principal motive: accompany someone | -7.69 | -25.92 | 10.55 | 9.27 | -.03 | .408 |
| Principal motive: « other » | -8.46 | -20.56 | 3.65 | 6.16 | -.05 | .170 |
| Perceived physical health | 0.95 | -6.69 | 8.59 | 3.88 | .01 | .807 |
| Level of incomes | 0.32 | -2.02 | 2.67 | 1.19 | .01 | .788 |
| Age | 0.09 | -0.21 | 0.40 | 0.16 | .03 | .547 |
| Habitat surface | -0.01 | -0.08 | 0.07 | 0.04 | -.01 | .877 |
| Attitude toward ASM | -2.66 | -4.80 | -0.52 | 1.09 | -.14 | .015 |
| Intention towards ASM | 1.76 | 0.17 | 3.34 | 0.80 | .12 | .030 |
| Attitude toward car | -2.06 | -4.21 | 0.08 | 1.09 | -.12 | .059 |
| Car habits | 1.19 | -0.97 | 3.35 | 1.10 | .06 | .278 |
| Number of cars in the household | 3.16 | -0.55 | 6.87 | 1.89 | .09 | .094 |
| Work percentage | -0.06 | -0.23 | 0.12 | 0.09 | -.03 | .509 |
| Number of children of 12 years and older | 0.50 | -3.51 | 4.51 | 2.04 | .01 | .806 |
| Number of persons in the household | -0.67 | -4.86 | 3.51 | 2.13 | -.03 | .752 |
| Educational attainment | -1.15 | -4.02 | 1.72 | 1.46 | -.03 | .432 |
| Perceived risks of getting COVID-19 | -1.05 | -3.15 | 1.04 | 1.07 | -.04 | .323 |
| Proximity to a public transport stop | -0.82 | -5.11 | 3.47 | 2.18 | -.02 | .707 |
| Frequency of simple trip chaining | -2.11 | -6.05 | 1.82 | 2.00 | -.05 | .292 |
| ASM during university | -0.34 | -4.44 | 3.76 | 2.08 | -.01 | .870 |
| Social norms of ASM | -0.19 | -2.22 | 1.83 | 1.03 | -.01 | .851 |
| ASM habits | 0.77 | -0.79 | 2.34 | 0.80 | .05 | .332 |
| Self-efficacy towards ASM | 0.15 | -1.51 | 1.80 | 0.84 | .01 | .863 |
| Number of children under 12 years | 1.70 | -3.51 | 6.90 | 2.65 | .04 | .523 |
| Moderate-to-vigorous PA | 0.00 | -0.01 | 0.00 | 0.00 | -.03 | .396 |
| Gender (man) | 1.13 | -3.88 | 6.14 | 2.55 | .02 | .657 |
| Green identity | 1.10 | -1.69 | 3.90 | 1.42 | .03 | .439 |
| ASM during elementary school | -0.12 | -2.99 | 2.76 | 1.46 | .00 | .937 |
| Frequency of medium trip chaining | 4.47 | -0.21 | 9.16 | 2.38 | .11 | .061 |
| Density of the domicile | 0.00 | 0.00 | 0.00 | 0.00 | .09 | .074 |
| Accessibility by car | -2.36 | -5.74 | 1.02 | 1.72 | -.05 | .171 |
| Possession of a bike x Intention | -1.01 | -1.68 | -0.35 | 0.34 | -.12 | .003 |
| Possession of a transport pass x Intention | 0.90 | -0.15 | 1.94 | 0.53 | .07 | .092 |
| Density of the domicile x Intention | 0.00 | 0.00 | 0.00 | 0.00 | -.08 | .091 |
| Accessibility by car x Intention | -1.00 | -2.41 | 0.41 | 0.72 | -.05 | .162 |
| **R^2^** | 0.55 |  |  |  |  |  |
| **Adjusted R^2^** | 0.50 |  |  |  |  |  |

Note: N_Model 4a_= 426. Dependent variable is percentage of active and sustainable mobility in a typical week. ASM = Active and sustainable mobility, PA = Physical activity. Women and work/study were used as reference dummy group for gender and principal motive. *b* = raw coefficient, *SE* *b*= Standard error of betas, *β* = Standardized betas, ^t^ represents p <.10, ^*^ p < .05, ^**^ p < .01, ^***^ p <.001. Values between brackets represent confidence interval.

Supplementary Table S10

*Slope of the interaction between posssessing a bike x intention towards ASM on biking/walking.*

| **Slope of Intention on percentage of biking/walking** | | | | |
| --- | --- | --- | --- | --- |
| **Variable** | **Slope** | **SE** | **t** | **p** |
| - 1 SD | -0.03  [-2.39 ; 2.32] | 1.20 | -0.03 | .980 |
| Average | 3.09^**^  [0.92 ; 5.25] | 1.10 | 2.80 | .010 |
| + 1 SD | 6.21^***^  [3.04 ; 9.38] | 1.61 | 3.85 | <.001 |

*SE* = Standard error.^*^ indicates *p* < .05, ^***^ indicates *p* < .001

Supplementary Table S11

*Slopes of the interaction between possessing a public transport pass x intention towards ASM on biking/walking.*

| **Slope of Intention on percentage of biking/walking** | | | | |
| --- | --- | --- | --- | --- |
| **Variable** | **Slope** | **SE** | **t** | **p** |
| - 1 SD | -4.04  [-8.25 ; 0.17] | 2.14 | -1.89 | .060 |
| Average | -6.82^***^  [-9.39 ; -4.24] | 1.31 | -5.21 | <.001 |
| + 1 SD | -9.72^***^  [-12.87 ; -6.33] | 1.66 | -5.77 | <.001 |

*SE* = Standard error.^***^ indicates *p* < .001

Supplementary Table S12

*Slopes of the interaction between posssessing a bike x intention towards ASM using public transport.*

| **Slope of Intention on percentage of using public transport** | | | | |
| --- | --- | --- | --- | --- |
| **Variable** | **Slope** | **SE** | **t** | **p** |
| - 1 SD | 0.28  [-1.84 ; 2.39] | 1.07 | 0.26 | .800 |
| Average | -2.10^*^  [-4.06 ; -0.14] | 1.00 | -2.11 | .042 |
| + 1 SD | -4.47^***^  [-7.32 ; -1.63] | 1.456 | -3.09 | <.001 |

*SE* = Standard error.^*^ indicates *p* < .05, ^***^ indicates *p* < .001


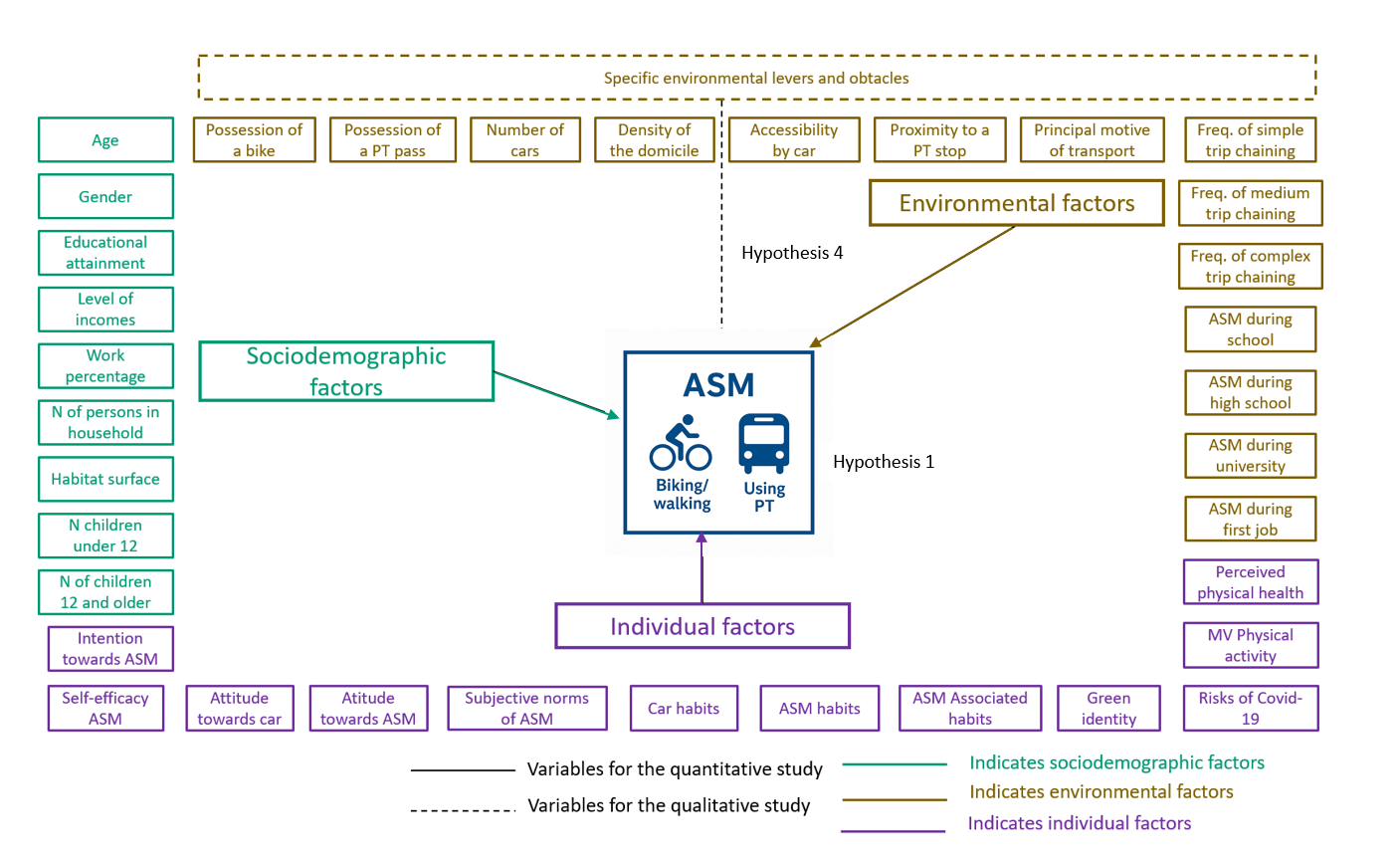


**Supplementary Figure 1.** Tested framework for Hypothesis 1 (Independent associations) and hypothesis 4 (Specific levers and obstacles).

Note. N= Number, ASM = Active and Sustainable mobility, PT = Public transport, Freq = Frequency. Icons were created with the assistance of OPENAI (ChatGPT).


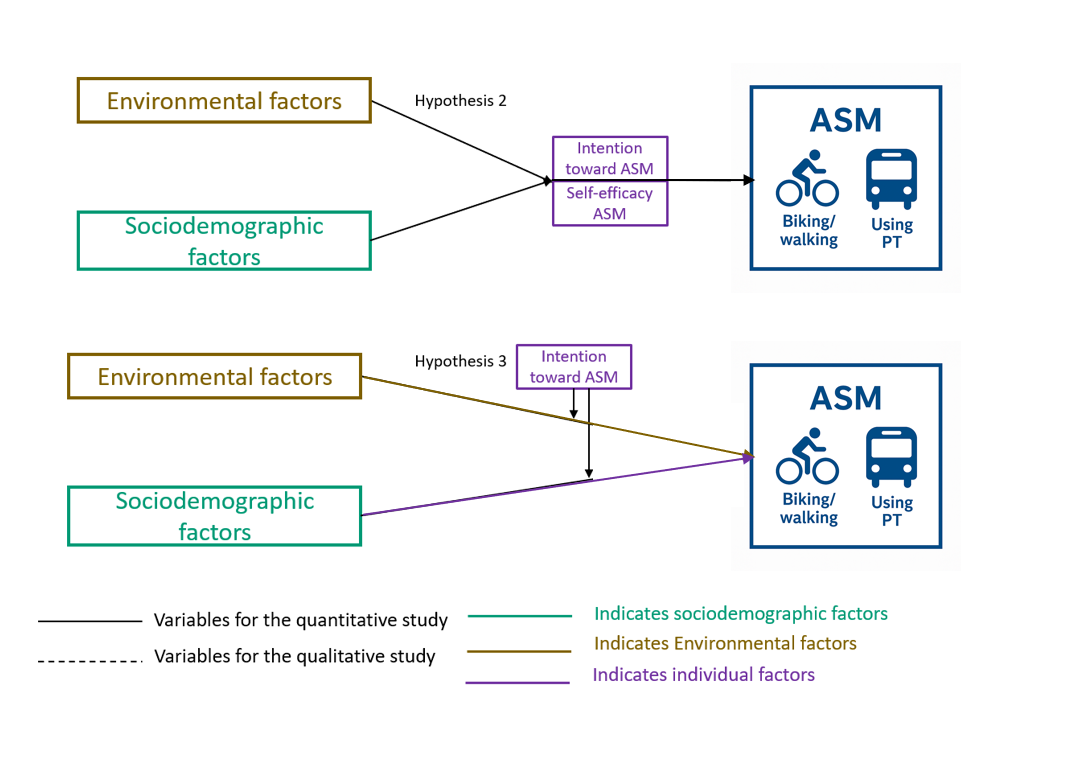


**Supplementary Figure 2.** Tested framework for Hypothesis 2 (Mediated associations) and Hypothesis 3 (Moderated associations). Icons were created with the assistance of OPENAI (ChatGPT).

Note. ASM = Active and Sustainable mobility.


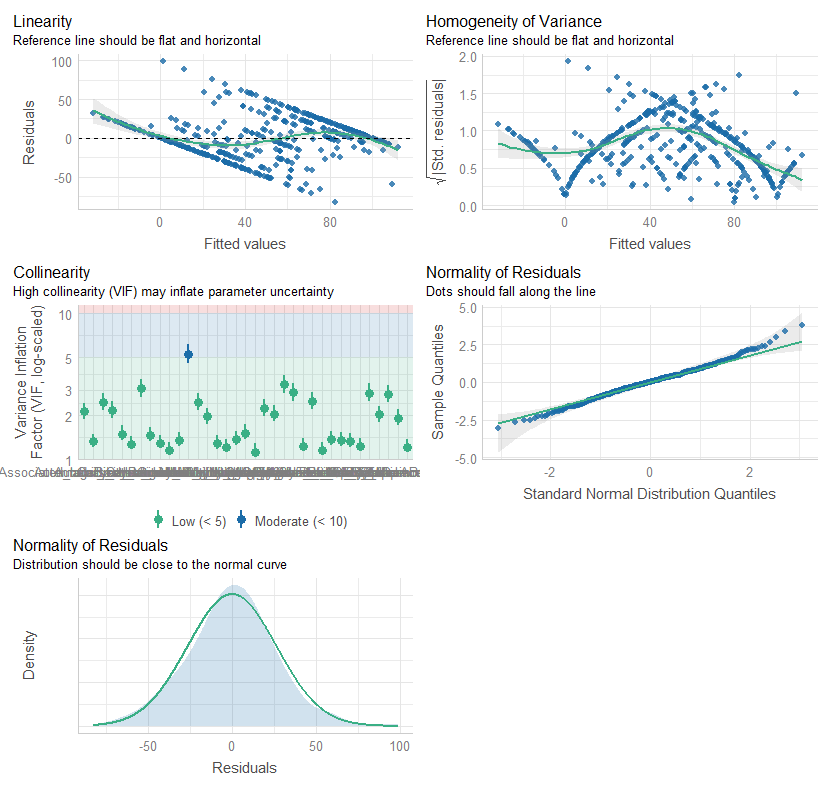


**Supplementary Figure 3.** Plot of model 1b (Model predicting biking/walking) assumptions (linearity, homogeneity of variance, collinearity, normality of residuals, and normality of random effects)**.** The blue dots represent the observations.


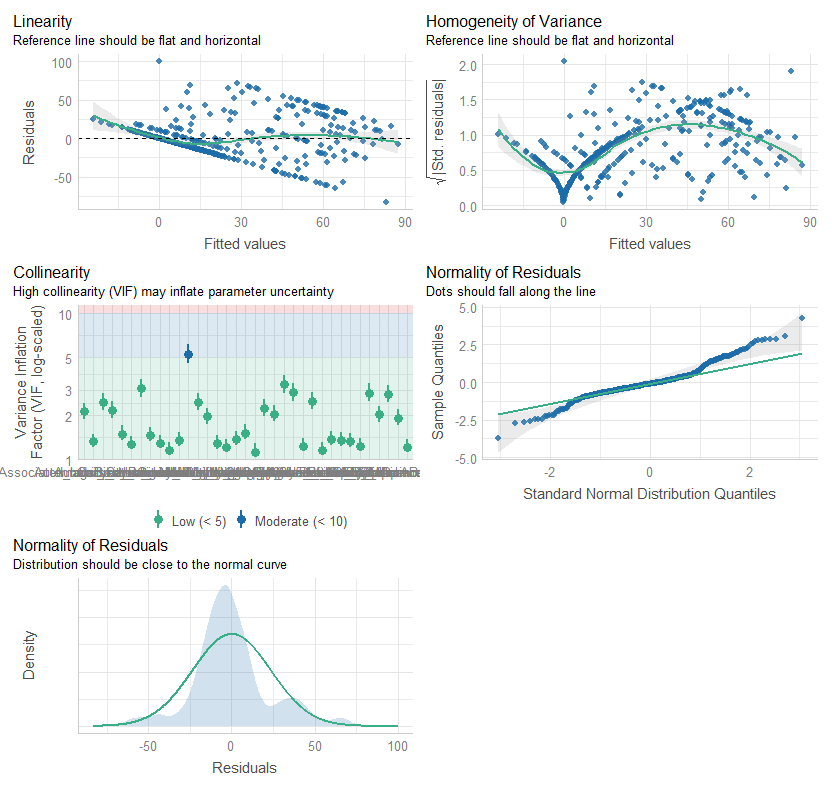


**Supplementary Figure 4.** Plot of model 2b (Model predicting using public transport) assumptions (linearity, homogeneity of variance, collinearity, normality of residuals, and normality of random effects)**.** The blue dots represent the observations.


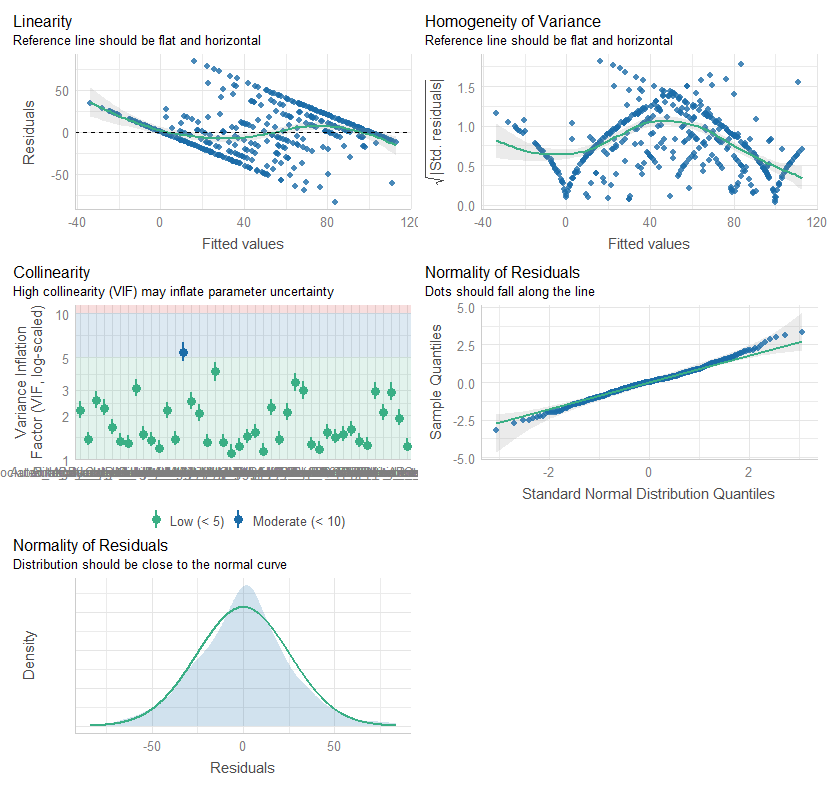


**Supplementary Figure 5.** Plot of model 4a (predicting biking/walking) assumptions (linearity, homogeneity of variance, collinearity, normality of residuals, and normality of random effects)**.** The blue dots represent the observations.


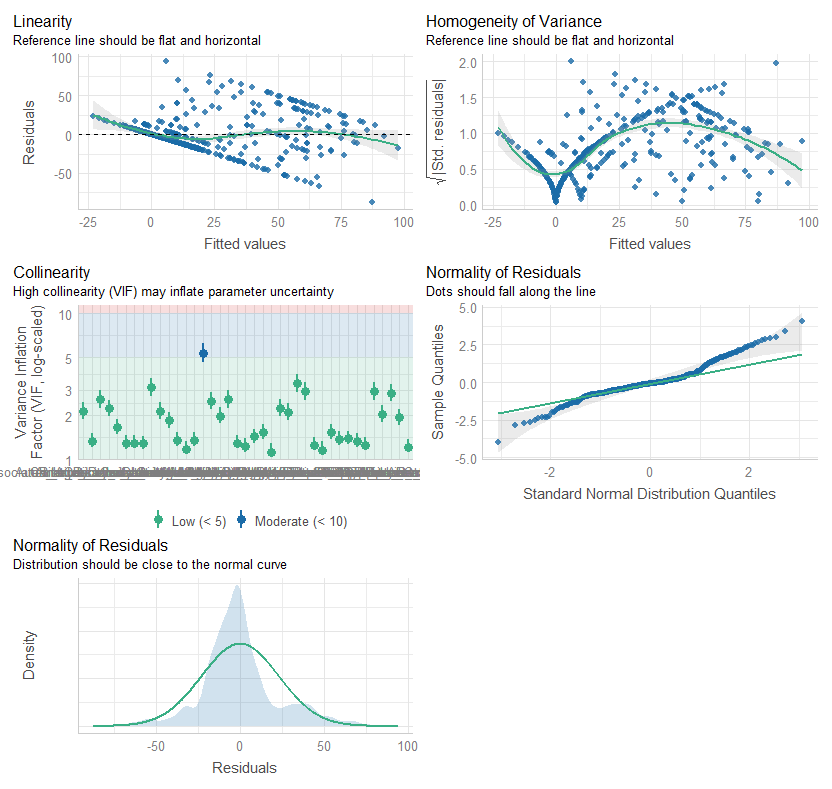


**Supplementary Figure 5.** Plot of model 4b (predicting using public transport) assumptions (linearity, homogeneity of variance, collinearity, normality of residuals, and normality of random effects)**.** The blue dots represent the observations.

Supplemental Material File 1: Presentation of the quantitative study and informed consent


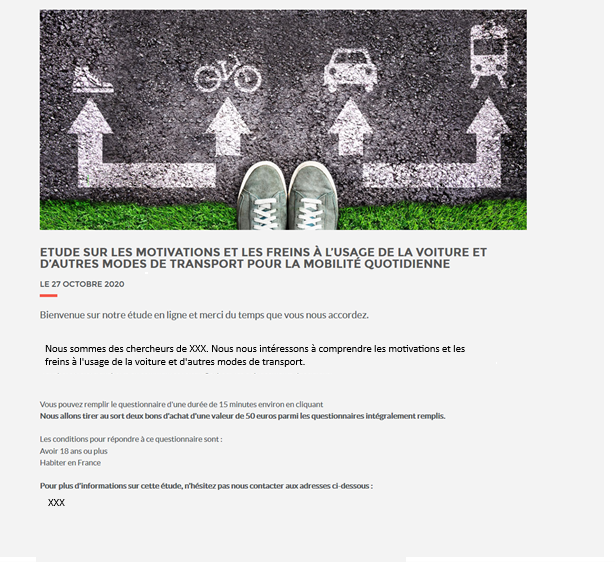

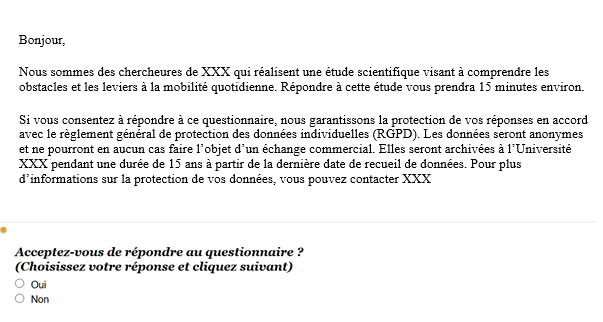


Supplemental Material File 2: Informed consent of the qualitative study

Names of those responsible for the study :

XXX

I agree to take part in this research after having read the following information:

1. The general objective of the focus group was explained to me before the interview. The more specific objectives and hypotheses will be explained at the end of the session.
2. My participation is voluntary. I am free to stop the experiment at any time. My decision will have no effect on my future relations with the laboratory and the university.
3. All information gathered during this interview will remain strictly confidential and will be used solely for research purposes.
4. My personal identity will be masked using an encrypted code, and my membership of an institution will be masked by a general description of my job (which does not include the name of my institution).
5. All data concerning me will be kept securely.
6. In accordance with the provisions of the French Data Protection Act and the General Data Protection Regulation (RGPD), I may request that my details and data be destroyed at any time.
7. In order to be able to analyse the discussion and interactions in their entirety, we would like to record this interview. The recording will only be accessible to the coordinator of this study (XXX), it will not be broadcast and it will be kept for 10 years on XXX server.
8. **I am aware of the health safety measures relating to COVID 19 in force at the research site and I undertake to comply with them.**

To be completed by the participant :

I'm over 18: ⬜Yes ⬜ No

I have read and understood the above information and willingly agree to take part in this research : ⬜Yes ⬜ No

Surname, First name - Date - Signature

**AUTHORISATION TO RECORD THE IMAGE**

**(Adult or emancipated person)**

The purpose of this application is to obtain the necessary consent and authorisations for the project specified below, on the understanding that the objectives of this project have been explained to the adult beforehand.

- **Project managers :**

**XXX**

- **Aim of the research project and methodology :**

This study looks at the obstacles and facilitators to the adoption of alternative mobility to the car as part of the InterMob study. You **will be filmed during the discussion.**

- **Mode of operation envisaged**

The images recorded will be used strictly for the purposes of this study and will be used to analyse the results. The recorded images will not be distributed and will be destroyed immediately at the oral or written request (please XXX the participant. The images will be stored for 10 years on the SENS laboratory server.

- **Authorisation of the adult or emancipated person**

Having regard to the Civil Code, in particular Article 9 on respect for privacy,

Having regard to the Intellectual Property Code,

This authorisation is subject to your signature, for the fixation of your image on an audiovisual medium as part of the research project described in paragraph 2 and for the modes of exploitation described in paragraph 3.

Your image/voice will be recorded under the authority of XXX.

Registration will take place on the dates, at the times and places indicated below:

**Date recorded :** During the interview

**Recording location:** XXX zoom platform

The beneficiary of the registration will exercise all the exploitation rights attached to this registration. The recording will remain its exclusive property. The beneficiary of the authorisation expressly undertakes not to transfer these authorisations to a third party.

It is also forbidden to use the recording of your image in any way that is illicit or not provided for above, in a way that is likely to harm your dignity, reputation or private life, and any other use that is prejudicial according to the laws and regulations in force.

In the context defined, the recording may not give rise to any remuneration or consideration in any form whatsoever. This express acceptance is final and excludes any subsequent request for remuneration.

**I declare that I am of legal age - an emancipated minor**  (delete as appropriate)

I acknowledge that I am fully vested with my personal rights.

**I acknowledge that I have read the above information and agree to the recording and use of my image exclusively for the above project:**  YES  NO

| **Full name: ............................................**  **Done at : ........................... Signature**  **On: ...............................** |
| --- |

Supplemental Material File 3: Physical activity and full scales of psychological constructs

Part 1

Age

| Age | How old are you (number) |
| --- | --- |

Type

| Sex | You are... (single choice)  Female / 2. male / 3. do not wish to reply |
| --- | --- |

Level of education

| Educationalattain | **What was the last qualification you obtained?** (single choice)  1. - No studies /2. BEP, CAP / 3. Bac / 4. Bac + 2 (BTS, DUT, DeUG, DUST) / 5. Bac + 3 (Licence) / 6. Bac + 5 (Master, diplôme grandes ecoles, diplôme detudes superieures specialisees)/ 7. Higher than Bac + 5 (doctorate) / 8. I do not wish to answer |
| --- | --- |

Revenue

| Income | **In which band does your household's total net monthly income currently fall?** (single choice)  By taking into account all your income, i.e. salaries, pensions, allowances and so on.  1. €1000 or less  2. Between €1001 and €1500  3. Between €1501 and €2000  4. Between 2001 and €3,000  5. Between €3001 and €4000  8. More than €4,000  9. Do not wish to reply |
| --- | --- |

Work situation

| Employmentstatus | What type of business are you in at the moment?  1. I work professionally (full-time, part-time, internship, apprenticeship, etc.) / 2. I work professionally (full-time, part-time, traineeship, apprenticeship, etc.) and I am studying. |
| --- | --- |
| If Employmentstatus = 1  Workpercentage | What is your percentage of work? (scale from 0 to 100) |
| If Employmentstatus = 1  Staggered_working_hours | Do you work staggered hours?  Example: Starting work very early in the morning (before 5am), very late in the evening (night work), in the afternoon or at weekends...  Never / 1. Rarely / 2. Occasionally / 3. Quite often / 4. Very often |
| If Employmentstatus = 1  Telework | Do you telework?  0. No, never / 1. Yes, a few times a month / 2. Yes, one day a week / 3. Yes, several days a week / 4. Yes, I always work from home |

Transport accessibility

| Numberofcar | **How many cars/motorbikes do you have in your household: __** (number) |
| --- | --- |
| If  Numberofcar  > 0  Drivingaccess | **How often do you drive your household's cars/motorbikes?** (single choice)  0 Never / 1. Once a week / 2. Several times a week / 3. Every day |
| Bikeaccess | **Do you have a bicycle (conventional or electric) / scooter / gyro-motor?** (single choice)  1. Yes, I own one and can use it whenever I want / 2. Yes, but I have to share it / 3. Yes, but it's broken down / unusable / 4. Yes, I have a subscription *(bike, scooter, etc) or another / 5*. No |
| Sharedtransportaccess | **Do you have a public transport/train/carpooling pass?** (single choice)  1. annual subscription / 2. monthly subscription / 3. weekly subscription / 4. No |

Part 2

Number of individuals in the household

| Household_number | Number of people in your household including yourself: __ (number)  **Help: a household is made up of** all the occupants of the same dwelling, who are not necessarily related to each other, except for those living with a partner. |
| --- | --- |

Number of children

| If Householdnumber >1  Numberofchildreunder12 | **Number of children under 12 in your household: __** (number) |
| --- | --- |
| If Householdnumber >1  Numberofchildreover12 | **Number of children aged 12 or over in your household: __** (number) |

Size of home

| Housing_size | **How many m2 do** you live in (number) |
| --- | --- |

Address/accessibility of accommodation

| Homeaddress | What is your home address?  Help: This question enables us to calculate the distances you travel. The questionnaire is anonymous, but if you do not wish to give your exact address. If the street is small, you can just give the address without the number. If it's a long street, you can give a number close to your own.   \| Homeaddress1**. Street number and name** \|  \| \| --- \| --- \| \| Homeaddress2**. Postcode / Name of municipality** \|  \| |
| --- | --- | --- | --- | --- | --- |
| If  Numberofcar  > 0  Caraccesshome | Do you have an **accessible parking** space (free or paid, including with a resident pass, etc.) near your home? (single choice)  1. no / 2. yes, more than 10 minutes' walk from home / 3. yes, less than 10 minutes' walk from home / 4. Don't know |
| Bikeaccesshome1 | Do you have bicycle parking (single choice)?  1. no / 2. yes, but it is not secure / 3. yes, it is secure / 4. I don't know |
| Ptaccesshome1 | Do you have a transport stop near your home?  1. no / 2. yes, more than 10 minutes' walk / 3. yes, less than 10 minutes' walk / 4. Don't know |

Change of situation following COVID-19

| Change. | **Have there been any changes affecting your movements since the containment and health situation due to COVID-19?** (multiple choice)  No  Yes |
| --- | --- |

Part 3

Current mobility behaviour

We're going to look at the **activities you do on a regular basis during a normal week** (excluding holidays, travel, unusual activities and situations).

| **Main activity** | |
| --- | --- |
| Principalactivity | **What is the main activity for which you travel most often during the week?** (single choice)  1. Work  2. Studies  3. Miscellaneous purchases and services (hairdresser, cobbler, bank, etc.)  4. Administrative procedures (Pôle Emploi, town hall, post office, CAF, etc.)  5. Health (medical and paramedical visits, health care, pharmacy)  6. Walk  7. Involvement in associations, charities, etc.  8. Sporting leisure activities  9. Cultural activities  10. Help for other people/relatives (friends, family, etc.)  11. Sociability and visits to friends/family  12. Accompanying/fetching someone  13. Other |

Principalactivitymob. In a typical week, what is the (approximate) percentage of your journeys **to your main activity**?

|  |  | 0%-10% | 11%-20% | 21%-30% | 31%-40% | 41%-50% | 51%-60% | 61%-70% | 71%-80% | 81%-90% | 91%-100% |
| --- | --- | --- | --- | --- | --- | --- | --- | --- | --- | --- | --- |
| Principalactivitymob1 | By car/motorbike/scooter |  |  |  |  |  |  |  |  |  |  |
| Principalactivitymob2 | By public transport (tram, bus, TER, TGV, Intercity)/Car (passenger or carpool) |  |  |  |  |  |  |  |  |  |  |
| Principalactivitymob3 | By bike (conventional or electric)/scooter/walking/other (rollerblades, gyroroue, etc). |  |  |  |  |  |  |  |  |  |  |

| **MAIN ACTIVITY 1** | |
| --- | --- |
| Fixedadressa1 | **Do you have one (or more) fixed address(es) for this activity?** (single choice)  **1.** Yes, I have a fixed address / 2. Yes, I have two or more fixed addresses / 3. Yes, I have one but I'm not at it very often (for example, I'm on business trips) */* 4. No |
| If FixedadressA1 = 1, 2  AddressActivity1 | Where do you most often go to carry out this activity? Help: This question enables us to calculate the distances you travel. The questionnaire is anonymous, but if you do not wish to give a precise address, you can do so. If the street is small, you can just give the address without the number. If you don't remember the precise address, you can mention the name of the company/association/store/school.   \| AddressActivity1a**. Street name and number**  Help: You can mention the name of the company if you can't remember the exact address. \|  \| \| --- \| --- \| \| AddressActivity1b**. Postcode /Name of municipality** \|  \| |
| AddressActivity1car | Do you have an **accessible parking** space (free or paid, with a season ticket, etc.) near this activity? (single choice)  1. no / 2. yes, more than 10 minutes' walk / 3. yes, less than 10 minutes' walk / 4. Don't know |
| AddressActivity1bike | Do you have bicycle parking near this activity? (single choice)  1. no / 2. yes, but it is not secure / 3. yes, it is secure / 4. I don't know |
| AddressActivity1pt | Do you have a transport stop near this activity?  1. no / 2. yes, more than 10 minutes' walk / 3. yes, less than 10 minutes' walk / 4. Don't know |

| **OTHER ACTIVITIES** | |
| --- | --- |
| Secondactivityyes | **Do you travel to another activity (work/study, shopping, socialising, community work, accompanying someone/fetching someone) several times a week?** (single choice)  0 No/ 1 Yes, 1 activity / 2. Yes, 2 activities / 3. Yes, 3 or more activities |

If Secondactivityyes = 1, 2 OR 3

| **Activity 2** | |
| --- | --- |
| Secondactivity | **What is the second activity for which you travel most often during the week?** (single choice)  1. Work  2. Studies  3. Miscellaneous purchases and services (hairdresser, cobbler, bank, etc.)  4. Administrative procedures (Pôle Emploi, town hall, post office, CAF, etc.)  5. Health (medical and paramedical visits, health care, pharmacy)  6. Walk  7. Involvement in associations, charities, etc.  8. Sporting leisure activities  9. Cultural activities  10. Help for other people/relatives (friends, family, etc.)  11. Sociability and visits to friends/family  12. Accompanying/getting someone  13. Other |

Secondactivitymob. In a typical week, what is the (approximate) percentage of your journeys **to your secondary activity?**

|  |  | 0%-10% | 11%-20% | 21%-30% | 31%-40% | 41%-50% | 51%-60% | 61%-70% | 71%-80% | 81%-90% | 91%-100% |
| --- | --- | --- | --- | --- | --- | --- | --- | --- | --- | --- | --- |
| Secondactivitymob1 | By car/motorbike/scooter |  |  |  |  |  |  |  |  |  |  |
| Secondactivitymob2 | By public transport (tram, bus, TER, TGV, Intercity)/Car (passenger or carpool) |  |  |  |  |  |  |  |  |  |  |
| Secondactivitymob3 | By bike (conventional or electric)/scooter/walking/other (rollerblades, gyroroue, etc). |  |  |  |  |  |  |  |  |  |  |

| **MAIN ACTIVITY 1** | |
| --- | --- |
|  | **How often do you use this activity to travel between your home and another activity (for example, going to [activity] on the way to and from work)?**  0.never /1. Rarely / 2. Often / 3. Always / 4. I don't know |
| Fixedadressa1 | **Do you have one (or more) fixed address(es) for this activity?** (single choice)  **1.** Yes, I have a fixed address / 2. Yes, I have two or more fixed addresses / 3. Yes, I have one but I'm not at it very often (for example, I'm on business trips) */* 4. No |
| If FixedadressA1 = 1, 2  AddressActivity1 | Where do you most often go to carry out this activity? Help: This question enables us to calculate the distances you travel. The questionnaire is anonymous, but if you do not wish to give your exact address. If the street is small, you can just give the address without the number. If it's a long street, you can give a number close to your own.   \| AddressActivity1a**. Street name and number**  Help: You can mention the name of the company if you don't remember the exact address. \|  \| \| --- \| --- \| \| AddressActivity1b**. Postcode / Name of municipality** \|  \| |
| AddressActivity1car | Do you have an **accessible parking** space (free or paid, with a season ticket, etc.) near this activity? (single choice)  1. no / 2. yes, more than 10 minutes' walk / 3. yes, less than 10 minutes' walk / 4. Don't know |
| AddressActivity1bike | Do you have bicycle parking near this activity? (single choice)  1. no / 2. yes, but it is not secure / 3. yes, it is secure / 4. I don't know |
| AddressActivity1pt | Do you have a transport stop near this activity?  1. no / 2. yes, more than 10 minutes' walk / 3. yes, less than 10 minutes' walk / 4. Don't know |

If Secondactivityyes = 2 OR 3

| **Activity 3** | |
| --- | --- |
| Thirdactivity | **What is the second activity for which you travel most often during the week?** (single choice)  1. Work  2. Studies  3. Miscellaneous purchases and services (hairdresser, cobbler, bank, etc.)  4. Administrative procedures (Pôle Emploi, town hall, post office, CAF, etc.)  5. Health (medical and paramedical visits, health care, pharmacy)  6. Walk  7. Involvement in associations, charities, etc.  8. Sporting leisure activities  9. Cultural activities  10. Help for other people/relatives (friends, family, etc.)  11. Sociability and visits to friends/family  12. Accompanying/getting someone  13. Other |

Thirdactivitymob. In a typical week, what is the (approximate) percentage of your journeys **to your secondary activity?**

|  |  | 0%-10% | 11%-20% | 21%-30% | 31%-40% | 41%-50% | 51%-60% | 61%-70% | 71%-80% | 81%-90% | 91%-100% |
| --- | --- | --- | --- | --- | --- | --- | --- | --- | --- | --- | --- |
| Thirdactivitymob1 | By car/motorbike/scooter |  |  |  |  |  |  |  |  |  |  |
| Thirdactivitymob2 | By public transport (tram, bus, TER, TGV, Intercity)/Car (passenger or carpool) |  |  |  |  |  |  |  |  |  |  |
| Thirdactivitymob3 | By bike (conventional or electric)/scooter/walking/other (rollerblades, gyroroue, etc). |  |  |  |  |  |  |  |  |  |  |

| **MAIN ACTIVITY 1** | |
| --- | --- |
|  | **How often do you use this activity to travel between your home and another activity (for example, going to [activity] on the way to and from work)?**  0.never /1. Rarely / 2. Often / 3. Always / 4. I don't know |
| FixedadressB1 | **Do you have one (or more) fixed address(es) for this activity?** (single choice)  **1.** Yes, I have a fixed address / 2. Yes, I have two or more fixed addresses / 3. Yes, I have one but I'm not at it very often (for example, I'm on business trips) */* 4. No |
| If FixedadressB1 = 1, 2  AddressActivity2 | Where do you most often go to carry out this activity? Help: This question enables us to calculate the distances you travel. The questionnaire is anonymous, but if you do not wish to give your exact address. If the street is small, you can just give the address without the number. If it's a long street, you can give a number close to your own.   \| AddressActivity2a**. Street name and number**  Help: You can mention the name of the company if you can't remember the exact address. \|  \| \| --- \| --- \| \| AddressActivity2b**. Postcode / Name of municipality** \|  \| |
| AddressActivity2car | Do you have an **accessible parking** space (free or paid, with a season ticket, etc.) near this activity? (single choice)  1. no / 2. yes, more than 10 minutes' walk / 3. yes, less than 10 minutes' walk / 4. Don't know |
| AddressActivity2bike | Do you have bicycle parking near this activity? (single choice)  1. no / 2. yes, but it is not secure / 3. yes, it is secure / 4. I don't know |
| AddressActivity2pt | Do you have a transport stop near this activity?  1. no / 2. yes, more than 10 minutes' walk / 3. yes, less than 10 minutes' walk / 4. Don't know |

Part 4

Travel chains

| Tripchain | Do you sometimes do several activities in different places that involve travelling between your home and your return home?  *For example, you leave home at 8am and go to work. You spend the day at work. At 5pm, before going home, you go shopping. Then you go home. In this example, you have carried out two activities (work, shopping) that require you to move around and that you follow on from each other before returning home. In the second example, you leave home to take your child to school (an activity that requires you to travel). You then go to the gym (a second activity requiring travel). Then you go shopping (a third activity requiring travel). Then you return home. You performed 3 activities between leaving home and returning home.*  0. Never, I always do an activity between leaving and returning home /1. Once or twice a week, I do several activities in different places between leaving and returning home/2. Three to five times a week / 3. Every day / 4. I don't know |
| --- | --- |
| TripchainA | **How often do you combine 2 activities/places that require you to travel between the time you leave and the time you return home?**  *For example, I leave my home to go to my place of study. I stop off at the gym before returning home.*  1. never / 2. A few times a month / 3. A few times a week / 4. Almost every day / 5. I don't know |
| TripchainB | **How often do you do 3 or 4 activities/places that require you to travel between the time you leave and the time you return home?**  *For example, I leave home to take my child to school. I go to work. I collect my child from school. We go shopping. You have performed 4 activities in different places between leaving home and returning home.*  0 Never / 1. A few times a month / 3. A few times a week / 4. Almost every day / 5. I don't know |
| TripchainC | **How often do you carry out 5 or more activities/places that require you to travel between the time you leave and the time you return home?**  *For example, I leave my home. I go to work. At lunchtime, I go and do some sport. Then I go to the bakery. Then I go back to work. After work, I'll meet some friends for a drink. Then we go to a show. You did more than 5 activities in different places between leaving home and returning home.*  0 Never / 1. A few times a month / 3. A few times a week / 4. Almost every day / 5. I don't know |

Mobility history

| Histomob | **Which mode.s of transport do you mainly use when :** |
| --- | --- |
| Histomob1 | **You were at primary school** (single choice)  1. By car (passenger) / 2. By public transport (tramway, bus, TER, TGV, Intercity) / 3. By bike (conventional or electric)/scooter/walking/Other (rollerblade, gyroroue, etc)/ 4. Not concerned |
| Histomob2 | **You were at college** (multiple choice)  1. By car (passenger) / 2. By public transport (tramway, bus, TER, TGV, Intercity) / 3. By bike (conventional or electric)/scooter/walking/Other (rollerblade, gyroroue, etc) / 4. Not concerned |
| Histomob3 | **You were at lycée** (multiple choice)  1. By car (passenger) / 2. By public transport (tramway, bus, TER, TGV, Intercity) / 3. By bike (conventional or electric)/scooter/walking/Other (rollerblade, gyroroue, etc) / 4. Not concerned |
| Histomob4 | **You were at university (or in training)** (multiple choice)  1. By car/motorbike/scooter / 2 By public transport (tram, bus, TER, TGV, Intercity)/Car (passenger or carpool) / 3. By bike (conventional or electric)/scooter/walking/Other (rollerblade, gyroroue, etc) / 4. Not concerned |
| Histomob5 | **You started your professional career** (multiple choice)  1. By car/motorbike/scooter / 2 By public transport (tram, bus, TER, TGV, Intercity)/Car (passenger or carpool) / 3. By bike (conventional or electric)/scooter/walking/Other (rollerblade, gyroroue, etc) / 4. Not concerned |

Part 5

Physical activity

**Physicalactivity.** We are interested in the different types of physical activity you do in your daily life. The following questions ask how much time you spend being physically active in **a typical week**. Answer each of these questions even if you don't consider yourself to be physically active. The questions concern the physical activities you do at work/university, when you're at home, when you're out and about, and in your free time. **In a typical week,** how much time do you spend doing the following?

**WalkingPA. Walking** (this includes walking to work/university and home, walking to get from one place to another, and any other type of walking you might have done in your free time for relaxation, sport or leisure).

___ minutes **per week** (number)

Help: 1h= 60 minutes, 2h = 120 minutes, 3h = 180 minutes, 4h = 240 minutes, 5h=300 minutes, 6h= 360 minutes, 7h = 420 minutes, 8h=480 minutes, 9h= 540 minutes, 10h = 600 minutes...

**ModeratePA.** Moderate **physical activity**

Moderate physical activity refers to activities that require you to **exert moderate physical effort and make it a little more difficult for you to breathe than normal** (for example, carrying light loads, vacuuming, leisurely cycling or playing volleyball).

___ minutes per **week** (number)

Help: 1h= 60 minutes, 2h = 120 minutes, 3h = 180 minutes, 4h = 240 minutes, 5h=300 minutes, 6h= 360 minutes, 7h = 420 minutes, 8h=480 minutes, 9h= 540 minutes, 10h = 600 minutes...

**VigorousPA.** Intense **physical activity**

Intense physical activity refers to activities that require a **great deal of physical effort and make it much more difficult for you to breathe than normal** (e.g. carrying heavy loads, digging, mountain biking or playing football).

___ minutes **per week** (number)

Help: Answer each of these questions even if you don't consider yourself to be physically active. The questions concern the physical activities you do at work/university, when you're at home, when you're out and about, and in your free time.

Perceived physical health

QV1. **Overall, do you think your health is:** 1. poor / 2. mediocre / 3. good / 4. Very good / 5. Excellent

**Because of your current state of health, are you limited in :**

QVQ2. **Moderate physical effort (moving a table, vacuuming, playing boules, etc.)?**

1. No, not at all limited / 2. Yes, somewhat limited / 3. Yes, very limited

QVQ3. **Climbing several flights of stairs**?

1. No, not at all limited / 2. Yes, somewhat limited / 3. Yes, very limited

**Over the last four weeks, and because of your physical condition :**

QVQ6: **Have you achieved less than you would have liked**?

1. Never / 2. Sometimes / 3. Often / 4. Most of the time / 5. Always

QVQ7. **Were you limited in what you could do**?

1. Never / 2. Sometimes / 3. Often / 4. Most of the time / 5. Always

3. Perceived risks

The following questions ask about your perceptions of covid-19

Perceived vulnerability

1. very much disagree / 2. Somewhat disagree / 3. Slightly disagree / 4. Neither disagree nor agree / 5. Slightly agree / 6. Somewhat agree / 7. Totally agree

PerceivedSuscep1. I have a high risk of catching coronavirus disease

PerceivedSuscep2. I'm concerned about the risk of catching the coronavirus

PerceivedSuscep13. I fall ill more easily than other people my age

Perceived seriousness

PerceivedSev1: Contracting the coronavirus could cause me serious health problems

PerceivedSev2. I'm afraid the Coronavirus will make me very ill

PerceivedSev3. I couldn't bear catching the Coronavirus because of my general state of health

Part 6

Intention

| Carintention | **In the coming month, do you intend to use a car/motorbike/scooter for most of your journeys?** (single choice)  1. no intention at all / 2. very little intention / 3. a little intention / 4. Moderately intentional / 5. Somewhat intentional / 6. Strongly intend / 7. Very strongly the intention |
| --- | --- |
| Othermodesintention | **In the coming month, do you intend to use a mode of transport other than the car/motorbike/scooter for most of your journeys? (**single choice)  1. no intention at all / 2. very little intention / 3. a little intention / 4. Moderately intentional / 5. Somewhat intentional / 6. Strongly intend / 7. Very strongly the intention |

Attitudes

| Carattitude1 | **For me, taking the car/motorbike/scooter for most of my journeys over the next month is...** (sole choice)  1. very optional / 2. Somewhat optional / 3. Slightly optional / 4. Neither optional nor essential / 5. Slightly essential / 6. Quite essential / 7. Very essential |
| --- | --- |
| Carattitude2 | **For me, taking the car/motorbike/scooter for most of my journeys over the next month is...** (unique choice)  1. very unpleasant / 2. Somewhat unpleasant / 3. Slightly unpleasant / 4. Neither unpleasant nor pleasant / 5. Slightly pleasant / 6. Quite pleasant / 7. Very pleasant |
| Othermodesattitude1 | **For me, taking a mode of transport other than the car/motorbike/scooter for most of my journeys over the next month is...** (sole choice)  1. very optional / 2. Somewhat optional / 3. Slightly optional / 4. Neither optional nor essential / 5. Slightly essential / 6. Quite essential / 7. Very essential |
| Othermodesattitude2 | **For me, taking a mode of transport other than the car/motorbike/scooter for most of my journeys over the next month is...** (sole choice)  1. very unpleasant / 2. Somewhat unpleasant / 3. Slightly unpleasant / 4. Neither unpleasant nor pleasant / 5. Slightly pleasant / 6. Quite pleasant / 7. Very pleasant |

Subjective norms

| Carnorms1 | **Most of the people who are important to me (family, friends, colleagues) encourage me to take the car/motorbike/scooter for most of my journeys...**  1. very much disagree / 2. Somewhat disagree / 3. Slightly disagree / 4. Neither disagree nor agree / 5. Slightly agree / 6. Somewhat agree / 7. Totally agree |
| --- | --- |
| Carnorms3 | **The proportion of people in my circle (friends, colleagues, family) who use cars/motorbikes/scooters for most of their journeys is**: (single choice)  1. No one / 2. One quarter (25%) / 3. One half / 4. Three quarters (75%) / 5. All the people |
| Othermodenorms1 | **Most of the people who are important to me (family, friends, colleagues) encourage me to use a mode of transport other than the car/motorbike/scooter for most of my journeys...** (unique choice)  1. very much disagree / 2. Somewhat disagree / 3. Slightly disagree / 4. Neither disagree nor agree / 5. Slightly agree / 6. Somewhat agree / 7. Totally agree |
| Othermodenorms3 | **The proportion of people in my circle (friends, colleagues, family) who use a mode of transport other than the car/motorbike/scooter to get around most of the time is**: (single choice)  1. No one / 2. One quarter (25%) / 3. One half / 4. Three quarters (75%) / 5. All the people |

Perception of control

| Selfefficacycar1 | **In the coming month, how confident are you in your ability to use the car/motorbike/scooter for most of your journeys?** (single choice)  1. not at all confident / 2. very little confident / 3. a little confident / 4. Moderately confident / 5. Somewhat confident / 6. Very confident / 7. Very strongly confident |
| --- | --- |
| Selfefficacyothermode1 | **In the coming month, how confident are you in your ability to use a mode of transport other than the car/motorbike/scooter for most of my journeys?** (single choice)  1. not at all confident / 2. very little confident / 3. a little confident / 4. Moderately confident / 5. Somewhat confident / 6. Very confident / 7. Very strongly confident |

Habits (automaticity)

| HabitscarA | Taking the **car/motorbike/scooter** to get around is something that : |
| --- | --- |
| HabitscarA1 | **I do it automatically** (single choice)  1. Very much disagree / 2. Somewhat disagree / 3. Slightly disagree / 4. Neither disagree nor agree / 5. Slightly agree / 6. Somewhat agree / 7. Totally agree |
| HabitscarA2 | **I do without thinking about it** (single choice)  1. Very much disagree / 2. Somewhat disagree / 3. Slightly disagree / 4. Neither disagree nor agree / 5. Slightly agree / 6. Somewhat agree / 7. Totally agree |
| HabitscarA3 | **I can do without paying attention** (single choice)  1. Very much disagree / 2. Somewhat disagree / 3. Slightly disagree / 4. Neither disagree nor agree / 5. Slightly agree / 6. Somewhat agree / 7. Totally agree |
| HabitscarA4 | **I'll start before I've even completed it** (single choice)  1. Very much disagree / 2. Somewhat disagree / 3. Slightly disagree / 4. Neither disagree nor agree / 5. Slightly agree / 6. Somewhat agree / 7. Totally agree |
| HabitsothermodeA | **Taking a mode of transport other than the car/motorbike/scooter to get around is something that :** |
| HabitsothermodeA1 | **I automatically** (single choice)  1. Very much disagree / 2. Somewhat disagree / 3. Slightly disagree / 4. Neither disagree nor agree / 5. Slightly agree / 6. Somewhat agree / 7. Totally agree |
| HabitsothermodeA2 | **I do without thinking about it** (single choice)  1. Very much disagree / 2. Somewhat disagree / 3. Slightly disagree / 4. Neither disagree nor agree / 5. Slightly agree / 6. Somewhat agree / 7. Totally agree |
| HabitsothermodeA3 | **I can do without paying attention** (single choice)  1. Very much disagree / 2. Somewhat disagree / 3. Slightly disagree / 4. Neither disagree nor agree / 5. Slightly agree / 6. Somewhat agree / 7. Totally agree |
| HabitsothermodeA4 | **I'll start before I've even completed it** (single choice)  1. Very much disagree / 2. Somewhat disagree / 3. Slightly disagree / 4. Neither disagree nor agree / 5. Slightly agree / 6. Somewhat agree / 7. Totally agree |

Habits (associated practices)

| HabitscarB | **The usual car/motorbike/scooter journeys can be an opportunity to carry out certain activities. When driving, how often do you...** |
| --- | --- |
| HabitscarB1 | **Listen to music / a radio programme / an audiobook / a podcast...** (single choice)  1. Never / 2. Sometimes / 3. Often / 4. Most of the time / 5. Always |
| HabitscarB2 | **Thinking about your organisation (work, studies, daily life)** (single choice)  1. Never / 2. Sometimes / 3. Often / 4. Most of the time / 5. Always |
| HabitscarB3 | **Read or reread documents / Write a message/SMS** (single choice)  1. Never / 2. Sometimes / 3. Often / 4. Most of the time / 5. Always |
| HabitscarB4 | **Call** (single choice)  1. Never / 2. Sometimes / 3. Often / 4. Most of the time / 5. Always |
| HabitscarB5 | **Other (Eating, drinking, smoking, hair or make-up, etc.)** (single choice)  1. Never / 2. Sometimes / 3. Often / 4. Most of the time / 5. Always |
| HabitsothermodeB | **The usual journeys using modes of transport other than the car/motorbike/scooter (cycling, public transport, walking, etc.) can provide an opportunity to carry out certain activities. When you're out and about, how often do you...** |
| HabitsothermodeB1 | **Listen to music / a radio programme / an audiobook / a podcast...** (single choice)  1. Never / 2. Sometimes / 3. Often / 4. Most of the time / 5. Always |
| HabitsothermodeB2 | **Thinking about your organisation (work, studies, daily life)** (single choice)  1. Never / 2. Sometimes / 3. Often / 4. Most of the time / 5. Always |
| HabitsothermodeB3 | **Read or reread documents / Write a message/SMS** (single choice)  1. Never / 2. Sometimes / 3. Often / 4. Most of the time / 5. Always |
| HabitsothermodeB4 | **Call** (single choice)  1. Never / 2. Sometimes / 3. Often / 4. Most of the time / 5. Always |
| HabitsothermodeB5 | **Other (Eating, drinking, smoking, hair or make-up, etc.)** (single choice)  1. Never / 2. Sometimes / 3. Often / 4. Most of the time / 5. Always |

Ecological identity

| Greenidentity | **Please rate the following statements using the following scale:** |
| --- | --- |
| Greenidentity1 | **I consider myself to be interested in environmental issues** (single choice)  1. Very much disagree / 2. Somewhat disagree / 3. Slightly disagree / 4. Neither disagree nor agree / 5. Slightly agree / 6. Somewhat agree / 7. Totally agree |
| Greenidentity2 | **I support sustainable development** (single choice)  1. Very much disagree / 2. Somewhat disagree / 3. Slightly disagree / 4. Neither disagree nor agree / 5. Slightly agree / 6. Somewhat agree / 7. Totally agree |
| Greenidentity3 | **I support renewable energy** (single choice)  1. Very much disagree / 2. Somewhat disagree / 3. Slightly disagree / 4. Neither disagree nor agree / 5. Slightly agree / 6. Somewhat agree / 7. Totally agree |
| Greenidentity4 | **I see myself as someone with an environmental conscience** (unique choice)  1. Very much disagree / 2. Somewhat disagree / 3. Slightly disagree / 4. Neither disagree nor agree / 5. Slightly agree / 6. Somewhat agree / 7. Totally agree |
| Greenidentity5 | **I consider myself "green"** (single choice)  1. Very much disagree / 2. Somewhat disagree / 3. Slightly disagree / 4. Neither disagree nor agree / 5. Slightly agree / 6. Somewhat agree / 7. Totally agree |

Supplemental Material File 4: Protocol of the qualitative study (experts)

**Introduction :**

I'd like to start by thanking you for coming to this focus group. By way of background, we are part of XXX, which is seeking to change the mobility behaviour of regular motorists living or working in Grenoble. The aim is to reduce air pollution in XXX and increase people's levels of physical activity. We've invited you to this discussion group because you work in or are involved with mobility in XXX. Your work experience and your associative/political experience as well as your opinions on the mobility change intervention that we are going to set up are very valuable to us. Feel free to express your opinions and experiences, you can agree or disagree with the other participants in this discussion, the most important thing is to discuss together.

Before starting the discussions, would you please sign the consent form and the image rights form? We would like to record these discussions so that we can transcribe all the exchanges and interactions.

**WHO ARE THEY (AS MOBILITY PROFESSIONALS) AND WHAT PREVIOUS EXPERIENCE HAVE THEY HAD IN THE FIELD OF MOBILITY?**

1. **To begin with, I'd like everyone to briefly introduce themselves (what is your job or profession, where do you currently work and what is your relationship with mobility?)**
2. **Have you had any experience of supporting or promoting alternative modes of transport to the car? If so, which ones?**

**Use this information to obtain a general perspective of the experience/profile of each expert, if the participants are not too talkative using this question.*

**Transition:** For the next few questions, we're going to focus mainly on the factors that enable/facilitate the choice of alternative modes of transport to the car.

**BASED ON THEIR EXPERIENCE, WHAT ARE THE LEVERS FOR CHANGING MOBILITY?**

1. **In your experience, what are the ideas or thoughts that make people want to use more alternatives to the car (cycling, walking, public transport, car pooling)?**

**Beware of the weather!*

1. **What are the resources (motivational, economic, physical, etc.) that enable a person to take more alternatives to the car (cycling, walking, public transport, carpooling)?**

**Beware of the weather!*

1. **If you had to choose one resource to provide to individuals looking to adopt an alternative mode of transport to the car, what resource would you choose?**

**This information should be used to consider factors not identified in the literature so that they can be added to the mobility questionnaire for the quantitative study. Observers tick the list of factors identified in the literature that are mentioned.*

**Transition:** For the next few questions, we're going to focus mainly on the factors that prevent people from using alternatives to the car.

**BASED ON THEIR EXPERIENCE, WHAT ARE THE OBSTACLES TO CHANGING MOBILITY?**

1. **In your experience, what are the ideas or thoughts that prevent/stop people from using alternative modes of transport (cycling, walking, public transport, carpooling) more than the car?**

**Beware of the weather!*

1. **What situations/contexts prevent people from using alternative modes of transport (cycling, walking, public transport, carpooling) more than the car?**

**Beware of the weather!*

**This information should be used to consider factors not identified in the literature so that they can be added to the mobility questionnaire for the quantitative study. Observers tick the list of factors identified in the literature that are mentioned.*

**FINAL EXPLANATION**

The objectives of this study are :

Compare the obstacles and levers identified by mobility experts and potential participants in the InterMob study.

Develop a tool to assess the skills and attitudes of a mobility coach (based on the opinions of experts and potential participants)

Identify the points to watch when implementing the InterMob intervention

Supplemental Material File 5: Protocol of the qualitative study (Car drivers aiming to reduce car use)

Introduction: I'd like to start by thanking you for attending this focus group. To give you a bit of context, we are part of XXX seeking to change the mobility behaviour of regular motorists living or working in XXX. We invited you to this focus group because you would like to/have recently started using the car less often for your journeys. Your past experiences with transport, your views on the obstacles and levers to taking up alternative modes of transport to the car (cycling, public transport, carpooling) and your expectations of the support we are going to provide are very valuable. Feel free to express your opinions and experiences, you can agree or disagree with the other participants in this discussion, the most important thing is to discuss together.

**WHAT ARE THEIR EXPERIENCES OF CHANGING MOBILITY?**

1. **To start with, I'd like everyone to introduce themselves (first name), and talk about their experiences with alternative modes of transport to the car (cycling, walking, public transport, carpooling)?**

**Use this information to obtain a general perspective of the experiences of potential participants in the focus group.*

Transition: For the next few questions, we're going to focus mainly on what, in your opinion, enables people to use alternative modes of transport to the car?

**BASED ON THEIR EXPERIENCE, WHAT ARE THE LEVERS FOR CHANGING MOBILITY?**

1. **In your experience, what are the ideas that make people want to use alternatives to the car (cycling, walking, public transport, car pooling)?**
2. **What resources (motivational, economic, physical, etc.) enable people to use alternatives to the car more often (cycling, walking, public transport, car pooling)**?

BASED ON **THEIR EXPERIENCE, WHAT ARE THE OBSTACLES TO CHANGING MOBILITY?**

1. **In your experience, what are the ideas or thoughts that prevent/stop a person from using alternative modes of transport (such as cycling, walking, public transport, car sharing) more than the car?**
2. **What situations/contexts prevent people from using alternative modes of transport (such as cycling, walking, public transport, carpooling) more than the car?**

**This information should be used to consider factors not identified in the literature so that they can be added to the mobility questionnaire for the quantitative study. Observers tick the list of factors identified in the literature that are mentioned.*
